# Synthesis and Antiproliferative Evaluation of 2-Deoxy-*N*-glycosylbenzotriazoles/imidazoles

**DOI:** 10.3390/molecules26123742

**Published:** 2021-06-19

**Authors:** Caleigh S. Garton, Noelle K. DeRose, Dylan Dominguez, Maria L. Turbi-Henderson, Ashley L. Lehr, Ashley D. Padilla, Scott D. Twining, Stephanie Casas, Chidozie O. Alozie, Azad L. Gucwa, Mohammed R. Elshaer, Michael De Castro

**Affiliations:** 1 Department of Chemistry, Farmingdale State College-SUNY, 2350 Broadhollow Rd, Farmingdale, NY 11735, USA; gartcs@farmingdale.edu (C.S.G.); derosenk@farmingdale.edu (N.K.D.); domida2@farmingdale.edu (D.D.); ashleylehr@yahoo.com (A.L.L.); decastmi@gmail.com (S.D.T.); casasl@farmingdale.edu (S.C.); chidoziealozie92@gmail.com (C.O.A.); 2 Department of Biology, Farmingdale State College-SUNY, 2350 Broadhollow Rd, Farmingdale, NY 11735, USA; turbihml@farmingdale.edu (M.L.T.-H.); padiad@farmingdale.edu (A.D.P.); gucwaal@farmingdale.edu (A.L.G.); 3 Department of Chemistry, Biochemistry and Physics, Fairleigh Dickinson University, Madison, NJ 07940, USA

**Keywords:** benzotriazole, D-glucal, cytotoxic activity, stereochemistry, heterocycles

## Abstract

A series of 2-deoxy-2-iodo-α-d-mannopyranosylbenzotriazoles was synthesized using the benzyl, 4,6-benzylidene and acetyl protected D-glucal in the presence of *N*-iodosuccinimide (NIS). Subsequent removal of the iodine at the C-2 position using tributyltin hydride under free radical conditions afforded the 2-deoxy-α-d-glucopyranosylbenzotriazoles in moderate to high yields. This method was extended to the preparation of substituted 2-deoxy-β-d-glucopyranosylimidazoles as well. The stereoselectivity of the addition reaction and the effect of the protecting group and temperature on anomer distribution of the benzotriazole series were also investigated. The anticancer properties of the newly synthesized compounds were evaluated in a series of viability studies using HeLa (human cervical adenocarcinoma), human breast and lung cancer cell lines. The *N*-[3,4,6-tri-*O*-benzyl-2-deoxy-α-d-glucopyranosyl]-1*H*-benzotriazole and the *N*-[3,4,6-tri-*O*-acetyl-2-deoxy-α-d-glucopyranosyl]-2*H*-benzotriazole were found to be the most potent cancer cell inhibitors at 20 µM concentrations across all four cell lines.

## 1. Introduction

Benzotriazoles are a versatile class of heterocycles with a wide range of applications and usages including antibacterial [1], antiviral [2] and antiparasitic [3], among others [4]. Compounds containing the benzotriazole moiety have been reported to possess powerful effects in inhibiting cell proliferation and arresting cancer development. For example, the [2-(4,5-dihydro-1*H*-imidazol-2-yl)-1*H*-benzotriazole]-dichlorocopper (II) complex has been reported to have very potent superoxide dismutase (SOD) activity inhibiting and preventing cancer cell growth [5]. Benzotriazole-containing alkanoic acids have also been demonstrated to be agonists of peroxisome proliferator-activated receptors (PPARs) and, as such, are capable of inducing apoptosis in multiple cell lines and in vivo tumors [6]. Carbohydrate-based benzotriazoles have also been found to inhibit solid tumor growth in mice. This includes the acetyl protected glucose and *N*-acetylglucosamine benzotriazole derivatives [7]. Substituted rybofuranosyl benzotriazoles and benzoimidazoles have also been reported to be powerful protein kinase CK2 inhibitors [8]. While the mechanism of action of the ribose derivatives is well-documented [9], little is known about the mode of action of *N*-glycosyl benzotriazoles.

Interest in the generation of 2-deoxy analogs of compound **I** arose from the abundance of reports on the biological activity displayed by 2-deoxy-d-glucose. This compound has been shown to significantly slow down cancer metabolism and induce cell death [10,11], yet cell viability studies using 2-deoxy-d-glucopyranosylbenzotriazoles cannot be found in the current literature. Various methods exist in the literature for the chemical synthesis of 2-deoxy sugars. Early work in this area focused on the chemical synthesis of 2-deoxy-β-glycosides [12,13]. Glycals have been used extensively as building blocks in the preparation of 2-deoxy sugars via their hydration or hydro-alkoxylation catalyzed by methanolic hydrogen halide [14,15,16]. Other methods include the use of methane sulfonic acid as catalyst [17], enzymes [16,18], alkoxymercuration of a glycal followed by reduction with sodium borohydride [19,20] and halohydration followed by dehydrogenation [21,22,23]. More closely related to this study is the method developed by Kashyap and co-workers via the reaction of various protected d-glucal with a mixture of tetra-*n*-butylammonium iodide with sodium periodate to afford 2-deoxyglycosides [24].

**Figure 1:**
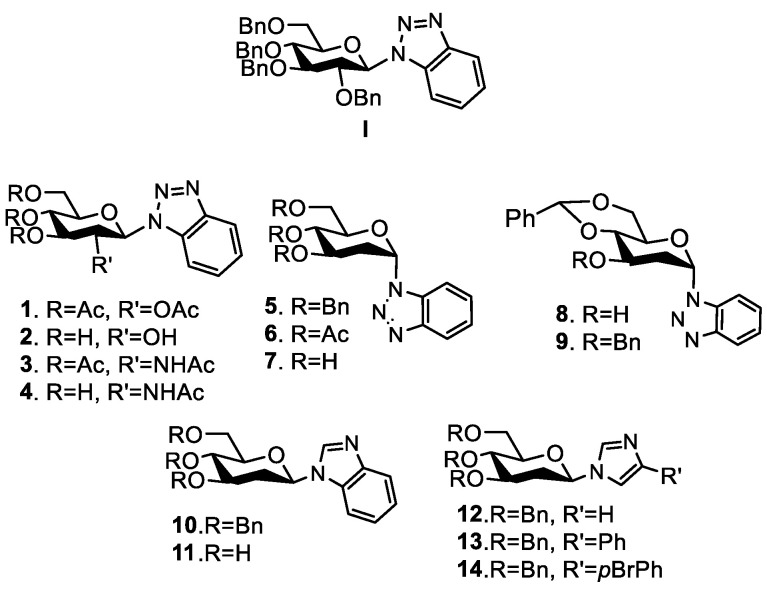
Targeted compounds.

We have previously reported the synthesis of the benzyl protected *N*-glycosyl benzotriazole **I** (Figure 1) using glycosyl halides and silver triflate (AgOTf) as starting materials. Compound **I** effectively inhibited growth of HeLa (cervical adenocarcinoma) cells (86%, 100 µM) [25]. The goal of this study was to chemically synthesize a series of analogs of compound **I** and evaluate their antiproliferative and cytotoxic effects using four different established human cancer cell lines (A549, HeLa, HCC827 and MDA-MB-231). Our initial efforts went into the generation of the known *N*-glycosyl benzotriazoles **1–4** (Figure 1) and the study of the effects of the protecting groups of the sugar on their viability as cancer drugs. Our laboratory also became interested in the chemical synthesis of various substituted 2-deoxy-d-glucopyranosylbenzotriazole/imidazoles **5–14** (Figure 1) and their antiproliferative properties were studied as well.

## 2. Results and Discussion

**Scheme 1:**
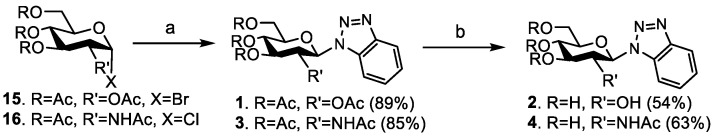
Addition of benzotriazoles to glycosyl halides. Reagent and conditions: (a) benzotriazole/AgOTf/ACN/rt/2 h; (b) NaOCH_3_/CH_3_OH.

Our work began with the synthesis of compounds **1–4** using an established procedure employing commercially available acetyl protected glycosyl halides **15** and **16** (Scheme 1) as starting materials [7].

Compounds **1–4** were initially tested using a range of concentrations (data not shown) in a series of viability studies using HeLa cell line. None of these compounds were capable of inhibiting cell growth, even at a concentration of 100 µM. We hypothesized that perhaps a lack of lipophilicity, especially for analogs **2** and **4** when compared to compound **I** (Figure 1), may have played a role in the uptake and crossing of the cell membrane in our cell assay giving rise to the poor results observed. Interestingly, this pattern was observed throughout our study, where the benzyl protected sugar outperformed the acetyl, benzylidene and fully deprotected constructs in subsequent viability studies.

**Scheme 2:**
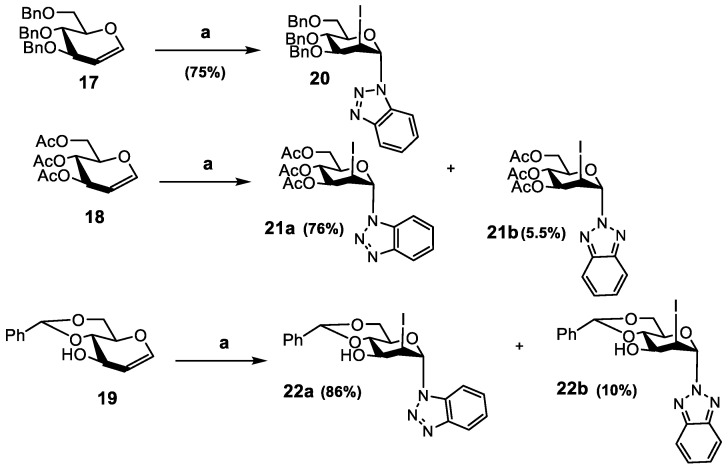
Addition of benzotriazole to glycal. Reagent and conditions: (a) NIS/benzotriazole/propionitrile/reflux/2 h. Yields were calculated as isolated yields.

In the next phase of our study, we proceeded to synthesize our 2-deoxy sugar analog series starting with the benzyl protected compound **5** (Figure 1). Our laboratory has extensive experience preparing 2-deoxy-*N*-glycosides and has previously reported the synthesis of compound **5** [25]. Access to compounds **5–10** (Figure 1) was achieved using 2-deoxy-2-iodo-α-mannopyranosylbenzotriazoles as precursors (Scheme 2). The benzyl protected d-glucal gave the α-mannose 1*H*-*N*-glycosylbenzotriazole as the single isomer **20** (*^3^J*_1-2_ = 4.0 Hz) [25], while a 13:1 (1*H*:2*H*-benzotriazole) ratio of the 2-deoxy-2-iodo-α-mannose isomer **21a** and **21b** was obtained for the acetyl protected sugar. This type of benzotriazole isomerization has been previously described in the literature [26]. In the case of the 4,6-benzylidene protected sugar, the 2-deoxy-2-iodo-α-mannose 1*H*-*N*-glycosylbenzotriazole isomer **22a** was obtained as the major product, while less than 10% of the 2-deoxy-2-iodo-α-mannose 2*H* isomer **22b** was isolated after column chromatography (Scheme 2). The addition reaction was repeated at 0 °C and room temperature and very similar product ratios were obtained for the acetyl and benzylidene protected sugar. The best yields were obtained when the reaction was refluxed for 2 h for the acetyl and 4,6-benzylidene protected d-glucal. Product ratios were determined by measuring the integration values of the anomeric protons of the crude mixture using ^1^H-NMR acetone-*d*_6_.

**Scheme 3:**
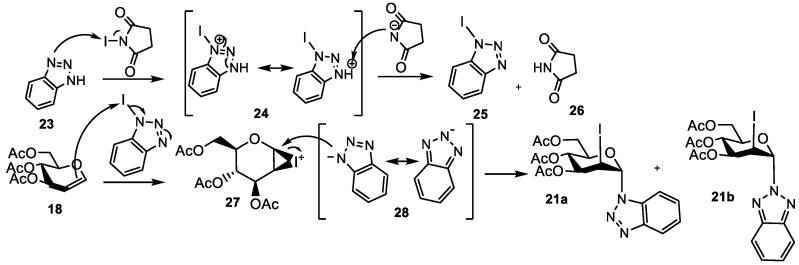
Formation of the 1*H* and 2*H*-glycosylbenzotriazole.

It is our hypothesis that the addition reaction of the benzotriazole to the acetyl and 4,6-benzylidene protected d-glucal may follow a mechanism very similar to the well documented addition of amides to glycals using NIS as the halogen source (Scheme 3) [27,28,29]. We envision the more nucleophilic benzotriazole **23** reacting with NIS generating intermediate **25**. The newly formed compound **25** delivers the iodine to the acetyl protected d-glucal **18**. Although the formation of compound **27** defies the majority rule [30,31], such an intermediate has been proposed in the isolation of the α-mannose isomer when alcohols are employed as the nucleophile in the presence of NIS [32,33,34]. Nucleophilic attacks by the benzotriazole anion from the bottom face of the three membered ring iodonium ion **27** give rise to the 2-deoxy-2-iodo-α-mannose isomers **21a** and **21b**.

**Scheme 4:**
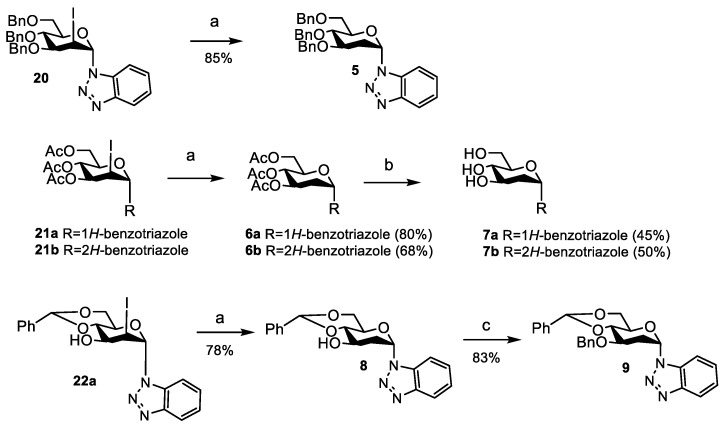
Preparation of 2-deoxy-α-d-glycosylbenzotriazole. Reagent and conditions: (a) Bu_3_SnH/AIBN/toluene/reflux/2 h; (b) NaOCH_3_/CH_3_OH; (c) NaH/BnBr/THF/rt.

Preparation of compounds **5–9** was accomplished via the removal of the iodine using tributyltin hydride (Bu_3_SnH) and 2,2′-azobis(2-methylpropionitrile) (AIBN) in toluene under refluxing conditions (Scheme 4).

**Scheme 5:**
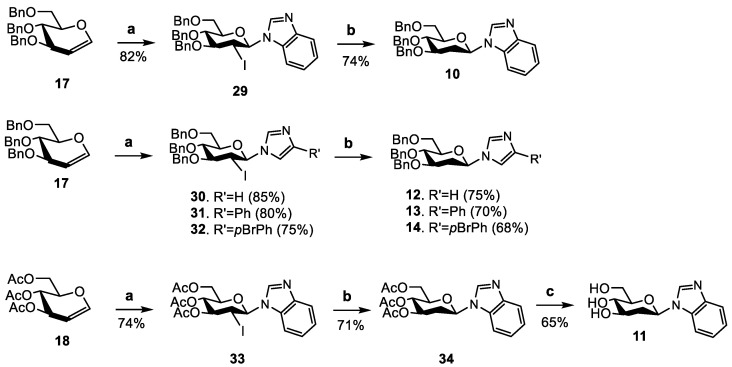
Preparation of 2-deoxy-β-d-glycosylimidazoles. Reagent and conditions: (a) NIS/imidazole/propionitrile/reflux/2 h; (b) Bu_3_SnH/AIBN/toluene/reflux/2 h; (c) NaOCH_3_/CH_3_OH.

Access to the benzyl protected 2-deoxy-β-d-glucopyranosylimidazoles **10–14** (Scheme 5) was achieved under free radical dehalogenation conditions using a procedure previously published by our laboratory [25]. We employed benzoimidazole and other commercially available substituted imidazoles. The addition reaction of the imidazole series resulted in the β-anomer **29–33** (Scheme 5) being the exclusive product in high to moderate yields. Such results can be explained based on the tendency of nitrogen glycosides to favor the equatorial and not axial position in virtue of the reverse anomeric effect [35].

**Figure 2:**
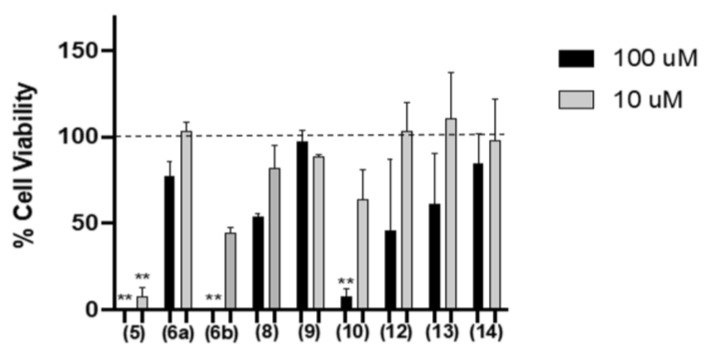
HeLa cell viability studies. Cells were treated with either a DMSO control (dashed line) or the synthesized compounds at a concentration of 100 µM (black bars) or 10 µM (grey bars).

The newly synthesized compounds **5–14** were initially tested for antiproliferative activity in a series of viability studies using HeLa cells. Those that demonstrated cytotoxicity are included in Figure 2. Cells were treated with the synthesized compounds at 10 µM (grey bars) and 100 µM (black bars) concentrations to determine which compounds exhibit cytotoxic effects before further evaluation.

Our results suggest that the benzyl protected 1*H*-2-deoxy-α-d-glucopyranosylbenzotriazole **5** was the most potent of all compounds and is capable of complete inhibition of cell viability at 100 µM and by 92% (8% survival) at 10 µM concentration. Our results also show a striking contrast in activity between the acetyl protected 1*H* (**6a**) and 2*H*-2-deoxy-α-d-glucopyranosylbenzotriazole **6b**. For compound **6b** (0%, 100µM) and (45%, 10 µM) cell survival was observed, while for compound **6a** (78%, 100 µM) and (100%, 10 µM), respectively. When comparing compound **5** and **6a** (acetyl protected analogue), we can observe a significant drop off in activity even at concentrations as high as 100 µM. As previously stated, we believe this may be attributed to the enhanced lipophilicity provided by the benzyl protecting group found in compound **5**, perhaps making it more effective at crossing cell membranes. More importantly, our results show that the isomer **6b** is as effective as compound **5** in killing our cancer cell lines at 100 µM, even with the acetyl protecting group present. We also observed moderate activity for the 4,6-benzylidene protected compound **8** (54%, 100 µM). Benzylation of the hydroxyl group at the C-3 position rendered compound **9** completely ineffective. From the glycoimidazole series, compound **10** showed the most promising results in terms of (8%, 100 µM) survival rate. The remaining substituted imidazoles gave very poor results at our tested concentrations. It should be worth mentioning that all the fully deprotected compounds showed no activity at either concentration (results not shown).

**Figure 3:**
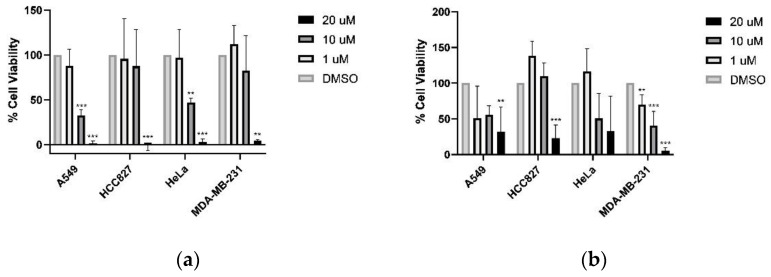
Viability studies using compound **5** (**a**) and compound **6b** (**b**).

Next, we set out to determine the cytotoxicity of compounds **5** and **6b** using established lung epithelial cancer cell lines A549 and HCC827 as well as the established metastatic breast cancer cell lines MDA-MB-231 and compared them to HeLa cells (Figure 3). Compounds **5** and **6b** proved to be excessively toxic at 100 µM concentrations, resulting with a zero percent survival rate. A dose–response analysis was performed to determine the appropriate concentration of compound required to inhibit the growth of cancer cells by 50% (IC_50_) in HeLa cells before proceeding. The IC_50_ was determined to be 2.908 µM for compound **5** and 9.940 µM for compound **6b**. DMSO was used as a control and the cells were treated similarly as in Figure 2, this time using 20 µM, 10 µM and 1 µM to determine their potency at lesser concentrations in other established cancer cell lines. On average, there was less than 5% survival rate across all cell lines tested at 20 µM for compound **5** and less than 24% for compound **6b**. The effectiveness of both compounds **5** and **6b** decreased as concentrations were lowered to 10 µM and 1 µM.

## 3. Materials and Methods

### 3.1. Chemistry

All ^1^H and ^13^C NMR spectra were recorded on an ECS 400 MHz JEOL spectrometer. ^1^H NMR spectra were recorded in acetone-*d*_6_ and are referenced to residual (CH_3_)_2_CO at *δ* = 2.04 ppm and the ^13^C NMR spectra are referenced to the peak at *δ* = 205 ppm. ^1^H NMR spectra recorded in CDCl_3_ are referenced to residual CHCl_3_ at *δ* = 7.24 ppm and ^13^C NMR spectra are referenced to the central peak of CDCl_3_ at *δ* = 77.0 ppm (See Supplementary Materials). Assignments were made by standard gCOSY and gHSQC experiments. HRMS data were obtained with a Bruker Ultraflex MALDI-TOF mass spectrometer. Tri-*O*-benzyl-d-glucal, tri-*O*-acetyl-d-glucal and *N*-iodosuccinimide (NIS) were purchased from Aldrich. Heterocycles were purchased from Aldrich Chemical Co. 4,6-*O*-Benzylidene-d-glucal was purchased from Carbosynth Ltd. All column chromatography was performed on silica gel 60 (EM Science, 70–230 mesh). Reactions were monitored by TLC on Kieselgel 60 F254 (EM Science) and the compounds were detected by examination under UV light and by charring with 10% sulfuric acid in methanol. Solvents were removed under reduced pressure at <40 °C. CH_3_CH_2_CN, CH_2_Cl_2_ was distilled from CaH_2_ and stored over molecular sieves (3 Å).

#### General Synthetic Procedures

Synthesis of compound **1–4** was performed as previously described [25]. Synthesis of the *N*-[2-deoxy-2-iodo-α-d-mannopyranosyl]-1*H*-benzotriazole series of compounds **20**, **21a**, **21b, 22a** and **22b** was performed as follows. d-glucal **17, 18** and **19** (1.83 mmol) was diluted in freshly distilled propionitrile (5 mL) followed by the addition of benzotriazole (3.67 mmol). NIS (2.74 mmol) was added to the reaction mixture and refluxed for 2 h. The reaction was cooled to room temperature and quenched with deionized water. The crude mixture was diluted in dichloromethane (50 mL) and washed with saturated Na_2_S_2_O_3_ solution (100 mL) and (3 × 100 mL) deionized water. The organic layer was dried over MgSO_4_, filtered and the solvent was removed under reduced pressure. The crude mixture was purified by flash column chromatography on silica gel (hexane/ethyl acetate eluent mixture).

Preparation of 2-deoxy-α-d-glycosylbenzotriazole series **5–9** was performed as follows. Compound **20**, **21a, 21b** and **22a** (1.38 mmol) was diluted in toluene (5 mL). Tributyltin hydride (2.76 mmol) was added dropwise followed by AIBN (1% by weight) and the resulting mixture was refluxed for 1 h. The reaction was allowed to cool down to room temperature and diluted in dichloromethane (50 mL). The organic layer was washed with deionized water (3 × 100 mL) and dried with MgSO_4_, followed by the subsequent removal of the solvent under reduced pressure. The crude mixture was purified by flash column chromatography on silica gel (hexane/ethyl acetate eluent mixture).

*N-[3,4,6-tri-O-benzyl-2-deoxy-α-d-glucopyranosyl]-1H-benzotriazole* (**5**), The crude mixture (0.51 g) was purified by flash column chromatography on silica gel (hexane/ethyl acetate, 5:1, *v/v*) to give a clear oil (0.40 g, 85%). ^1^H NMR (400 MHz, acetone-*d*_6_) *δ* 8.01 (d, 1H, *J* = 8.0 Hz, Ar), 7.88 (d, 1H, *J* = 8.4 Hz, Ar), 7.39–7.29 (m, 9H, Ar), 7.28–7.23 (m, 8H, Ar), 6.56 (d, 1H, *J*_1,2_ = 5.6 Hz, H-1), 4.76 (dd, 2H, *J* = 12.8 Hz, *J* = 10.8 Hz, C*H*_2_-Ph), 4.66 (d, 1H, *J* = 12.6 Hz, C*H*_2_-Ph), 4.52 (d, 1H, *J* = 10.8 Hz, C*H*_2_-Ph), 4.41–4.29 (m, 3H, H-3, C*H*_2_-Ph), 3.63-3.58 (m, 2H, H-4, H-6′), 3.47 (d, 1H, *J*_5,4_ = 10.8 Hz, H-5), 3.25 (dd, 1H, *J*_2,3_ = 8.0 Hz, *J*_2,1_ = 4.8 Hz, H-2), 3.12 (dd, 1H, *J*_6,5_ = 5.2 Hz, *J*_6,6′_ = 4.2 Hz, H-6), 2.21–2.16 (dd, 1H, *J*_2′,3_ = 8.5 Hz, *J*_2′,1_ = 4.4 Hz, H-2′) ppm. ^13^C NMR (125 MHz, acetone-*d*_6_) *δ* 146.27, 137.9, 137.6, 137.5, 133.4, 128.25, 127.82, 127.74, 127.8, 127.61, 126.2, 111.61, 82.11, 77.60, 77.47, 74.33, 70.45, 72.81, 71.33, 69.04, 32.28 ppm. HR-MALDI-Tof/MS: *m/z* calculated for C_33_H_33_N_3_NaO_4_ 558.2369; found 558.2365 [M + Na]^+^.

*N-[3,4,6-tri-O-acetyl-2-deoxy-α-d-glucopyranosyl]-1H-benzotriazole* (**6a**), The crude mixture (0.59 g) was purified by flash column chromatography on silica gel (hexane/ethyl acetate, 3:1, *v/v*) to give a white solid (0.43 g, 80%). ^1^H NMR (400 MHz, acetone-*d*_6_) *δ* 7.95 (d, 1H, *J* = 8.0 Hz, Ar), 7.75 (d, 1H, *J* = 8.1 Hz, Ar), 7.50 (t, 1H, *J* = 8.1 Hz, *J* = 7.9 Hz, Ar), 7.35 (t, 1H, *J* = 8.2 Hz, *J* = 7.9 Hz, Ar), 6.58 (d, 1H, *J*_1,2_ = 5.6 Hz, H-1), 5.81 (m, 1H, 3-H), 5.01 (dd, 1H, *J*_4,3_ = 9.6 Hz, *J*_4,5_ = 9.6 Hz, H-4), 4.10 (dd, 1H, *J*_6,5_ = 6.8 Hz, *J*_6,6′_ = 5.6 Hz, H-6), 3.79 (dd, 1H, *J*_6′,5_ = 6.5 Hz, *J*_6′,6_ = 5.8 Hz, H-6′), 3.33 (m, 1H, H-5), 3.12 (dd, 1H, *J*_2,1_ = 6.0 Hz, *J*_2,2′_ = 2.5 Hz, H-2), 2.42–2.39 (ddd, 1H, *J*_2′,3_ = 7.6 Hz, *J*_2′,1_ = 5.6 Hz, *J*_2′,2_ = 2.5 Hz, H-2′), 1.91–1.06 (s, 9H, C*H*_3_) ppm. ^13^C NMR (125 MHz, acetone-*d*_6_) *δ* 169.72, 169.45, 146.27, 132.96, 127.95, 124.55, 119.59, 111.20, 81.15, 70.41, 69.18, 68.97, 61.81, 32.04, 20.07, 19.85, 19.77 ppm. HR-MALDI-Tof/MS: *m/z* calculated for C_18_H_21_N_3_NaO_7_ 414.1277; found 414.1276 [M + Na]^+^.

*N-[3,4,6-tri-O-acetyl-2-deoxy-α-d-glucopyranosyl]-2H-benzotriazole* (**6b**), The crude mixture (0.062 g) was purified by flash column chromatography on silica gel (hexane/ethyl acetate, 3:1, *v/v*) to give a white solid (0.028 g, 68%). ^1^H NMR (400 MHz, acetone-*d*_6_) *δ* 7.81 (d, 2H, *J* = 8.0 Hz, Ar), 7.36 (dd, 2H, *J* = 7.9 Hz, Ar), 6.56 (d, 1H, *J*_1,2_ = 5.6 Hz, H-1), 5.97 (dd, 1H, *J*_3,4_ = 8.9 Hz, *J*_3,2_ = 7.0 Hz, H-3), 5.06 (dd, 1H, *J*_4,5_ = 9.4 Hz, *J*_4,3_ = 9.2 Hz, H-4), 4.11 (dd, 1H, *J*_6,5_ = 9.4 Hz, *J*_6,6′_ = 4.8 Hz, H-6), 3.84–3.80 (m, 2H, H-5, H-6′), 2.84 (dd, 1H, *J*_2,1_ = 5.0 Hz, *J*_2,2′_ = 2.4 Hz, H-2), 2.37 (ddd, 1H, J_2′,3_ = 7.0 Hz, *J*_2′,1_ = 6.0 Hz, *J*_2′,2_ = 3.0 Hz, H-2′), 1.89–1.79 (s, 9H, C*H*_3_) ppm. ^13^C NMR (125 MHz, acetone-*d*_6_) *δ* 169.24, 169.45, 145.00, 132.96, 127.95, 124.55, 119.59, 111.20, 81.15, 70.41, 69.18, 68.97, 61.81, 32.04, 29.63, 29.04, 28.85, 28.47, 20.07, 19.85, 19.77 ppm. HR-MALDI-Tof/MS: *m/z* calculated for C_18_H_21_N_3_NaO_7_ 414.1277; found 414.1276 [M + Na]^+^.

*N-[2-deoxy-α-d-glucopyranosyl]-2H-benzotriazole* (**7a**). The crude mixture (0.301 g) was purified by flash column chromatography on silica gel (CHCl_3_/CH_3_OH, 9:1, *v/v*) to give a white solid (0.13 g, 45%). ^1^H NMR (400 MHz, CD_3_OD) *δ* 7.90 (d, 1H, *J* = 8.0 Hz, Ar), 7.83 (t, 1H, *J* = 10.4 Hz, *J* = 8.1 Hz, Ar), 7.47 (t, 1H, *J* = 7.6 Hz, *J* = *7.2* Hz, Ar), 7.36 (t, 1H, *J* = 7.6 Hz, *J* = 7.6 Hz, Ar), 6.43 (d,1H, *J*_1,2_ = 5.2 Hz, H-1), 4.30–4.24 (m, 1H, H-3), 3.62–3.54 (m, 2H, H-6), 3.38 (dd, 1H, *J*_4,5_ = 10.0 Hz, *J*_4,3_ = 9.6 Hz, H-4), 3.05 (dd, 1H, *J*_2,1_ = 5.2 Hz, *J*_2,2′_ = 2.1 Hz, H-2), 2.82–2.79 (m, 1H, H-5), 2.19–2.11 (ddd, 1H, *J*_2′,3_ = 7.0 Hz, *J*_2′,2_ = 6.1 Hz, *J*_2′,1_ = 5.2 Hz, H-2′) ppm. ^13^C NMR (125 MHz, CD_3_OD) *δ* 145.69, 133.01, 127.78, 124.74, 118.51, 111.64, 82.88, 75.23, 71.16, 68.94, 60.93, 34.51 ppm. HR-MALDI-Tof/MS: *m/z* calculated for C_12_H_15_N_3_NaO_4_ 288.0960; found 288.0957 [M + Na]^+^.

*N-[2-deoxy-α-d-glucopyranosyl]-2H- benzotriazole* (**7b**). The crude mixture (0.041 g) was purified by flash column chromatography on silica gel (CHCl_3_/CH_3_OH, 9:1, *v/v*) to give a white solid (0.019 g, 50%). ^1^H NMR (400 MHz, CD_3_OD) *δ* 8.61 (dd, 2H, *J =* 3.2 Hz, *J =* 3.2 Hz, Ar), 8.18 (dd, 2H, *J =* 2.8 Hz, *J =* 2.8 Hz, Ar), 7.26 (d, 1H, *J*_1,2_ = 5.6 Hz, H-1), 5.28–5.24 (m, 1H, H-3), 4.49–4.44 (m, 2H, H-6), 4.24 (dd, *J*_4,3_ = 9.6 Hz, *J*_4,5_ = 9.2 Hz, H-4), 4.12–4.08 (m, 1H, H-5), 3.61 (dd, 1H, *J*_2,1_ = 5.2 Hz, *J*_2,2′_ = 2.8 Hz, H-2), 2.99–2.91 (ddd, 1H, *J*_2′,3_ = 7.6 Hz, *J*_2′,2_ = 6.0 Hz, *J*_2′,1_ = 5.1 Hz, H-2′) ppm. ^13^C NMR (125 MHz, CD_3_OD) *δ* 144.08, 126.91, 118.00, 88.37, 76.21, 71.06, 68.38, 60.94, 35.16 ppm. HR-MALDI-Tof/MS: *m/z* calculated for C_12_H_15_N_3_O_4_ 265.1063; found 288.0960 [M + Na]^+^.

*N-[4,6-O-benzylidene-3-hydroxyl-2-deoxy-α-d-glucopyranosyl]-1H-benzotriazole* (**8**). The crude mixture (0.41 g) was purified by flash column chromatography on silica gel (hexane/ethyl acetate, 2:1, *v/v*) to give a white solid (0.28 g, 78%). ^1^H NMR (400 MHz, acetone-*d*_6_) *δ* 7.93 (d, 1H, *J =* 7.6 Hz, Ar), 7.80 (d, 1H, *J =* 8.0 Hz, Ar), 7.40 (t, 1H, *J =* 8.2 Hz, *J =* 2.0 Hz, Ar) 7.27–7.25 (m, 6H, Ar), 6.54 (dd, 1H, *J*_1,2_ = 5.6 Hz, 1-H), 5.54 (s, 1H, C*H*-Ph), 4.71 (d, 1H, *J* = 4.0 Hz, OH), 4.58 (dd, 1H, *J*_3,4__=_ 7.2 Hz, *J*_3,2_ = 6.0 Hz, 3-H), 3.85 (d,1H, *J_4,3_ =* 6.8 Hz, *J*_4,5_ = 5.2 Hz, 4-H), 3.63 (m, 2H, 5-H, 6-H), 3.03 (dd,1H, *J*_2,1_ = 5.6 Hz, *J*_2,2_ = 3.8 Hz, 2-H), 2.29 (dd, 1H, *J*_2′,1_ = 5.5 Hz, *J*_2′,2_ = 4.0 Hz, H-2′) ppm. ^13^C NMR (125 MHz, acetone-*d*_6_) *δ* 146.23, 138.24, 133.03, 128.74, 127.44, 127.86, 126.45, 124.40, 119.54, 111.09, 101.58, 83.40, 82.37, 68.07, 65.88, 65.77, 65.28, 35.26 ppm. HR-MALDI-Tof/MS: *m/z* calculated for C_19_H_19_N_3_O_4_ 353.1376; found 354.1454 [M + H]^+^.

*N-[4,6-O-benzylidene-3-O-benzyl-2-deoxy-α-d-glucopyranosyl]-1H-benzotriazole* (**9**). The crude mixture (0.81 g) was purified by flash column chromatography on silica gel (hexane/ethyl acetate, 2:1, *v/v*) to give a white solid (0.58g, 83%). ^1^H NMR (400 MHz, acetone-*d*_6_) *δ* 7.94 (d, 1H, *J* = 8.0 Hz, *J* = 5.2 Hz, Ar), 7.72 (d, 1H, *J* = 8.4 Hz, Ar), 7.49–7.45 (m, 1H, Ar), 7.36–7.31 (m, 5H, Ar), 7.29–7.14 (m, 5H, Ar), 7.16 (t, 1H, *J* = 8.2 Hz, *J* = 6.1 Hz, Ar), 6.58 (d, 1H, *J*_1,2_ *=* 5.6 Hz, H-1), 5.62 (s, 1H, CHPh), 4.73 (dd, 2H, *J* = 12.6 Hz, *J* = 10.5 Hz, CH_2_Ph), 4.55–4.50 (m, 1H, H-3), 3.91–3.84 (m, 2H, H-4, H-5), 3.71 (t, 1H, *J*_6,5_ = 10.4 Hz, *J*_6,6′_ = 10.4 Hz, H-6), 3.19–3.08 (m, 2H, H-2, H-6), 2.36–2.29 (m, 1H, H-2′) ppm. ^13^C NMR (125 MHz, acetone-*d*_6_) *δ* 146.20, 138.15, 128.71, 128.20, 128.02, 127.92, 127.55, 127.34, 126.24, 124.45, 119.57, 111.02, 101.25, 82.89, 82.18, 73.75, 72.27, 68.12, 65.25, 33.71 ppm. HR-MALDI-Tof/MS: *m/z* calculated for C_26_H_25_N_3_O_4_ 443.1845; found 444.1923 [M + H]^+^.

*N-[3,4,6-tri-O-benzyl-2-deoxy-β-d-glucopyranosyl]-1H-benzoimidazole* (**10**). The crude mixture (0.62 g) was purified by flash column chromatography on silica gel (hexane/ethyl acetate, 2:1, *v/v*) to give a white solid (0.39g, 74%). ^1^H NMR (400 MHz, CDCl_3_) *δ* 7.95 (s, 1H, NC*H*N), 7.71 (d, 1H, *J* = 5.2 Hz, Ar), 7.41 (d, 1H, *J* = 4.0 Hz, Ar), 7.26–7.16 (m, 18H, Ar), 5.44 (d, 1H, *J*_1,2_ = 10.8 Hz, H-1), 4.87 (d, 1H, *J* = 10.8 Hz, C*H*_2_Ph), 4.61 (dd, 2H, *J* = 11.6 Hz, *J* = 10.2 Hz, C*H*_2_Ph), 4.49–4.39 (m, 3H, C*H*_2_Ph), 3.81–3.75 (m, 1H, H-3), 3.73–3.60 (m, H-4, H-5, H-6), 2.47 (dd, 1H, *J*_2,1_ = 9.6 Hz, *J*_2,2′_ = 2.8 Hz, H-2), 2.25 (dd, 1H, *J*_2,1_ = 11.6 Hz, *J*_2,2′_ = 3.8 Hz, H-2′) ppm. ^13^C NMR (125 MHz, CDCl_3_) *δ* 143.00, 139.46, 137.21, 137.12, 137.03, 131.96, 127.61, 127.51, 126.84, 122.51, 121.93, 119.61, 110.26, 80.43, 78.60, 77.02, 74.39, 72.61, 71.09, 67.88 ppm. HR-MALDI-Tof/MS: *m/z* calculated for C_34_H_34_N_2_O_4_ 534.2519; found 535.2597 [M + H]^+^.

*N-[2-deoxy-β-d-glucopyranosyl]-1H-benzoimidazole* (**11**). The crude mixture (0.75 g) was purified by flash column chromatography on silica gel (CHCl_3_/CH_3_OH, 9:1 *v/v*) to give a white solid (0.31g, 65%). ^1^H NMR (400 MHz, CD_3_OD) *δ* 8.28 (d, 1H, *J* = 7.2 Hz, NC*H*N), 7.75 (d, 1H, *J* = 8.4 Hz, Ar), 7.26–7.19 (m, 2H, Ar), 6.10 (d, 1H, J_1,2_ = 10.0 Hz, H-1), 4.00–3.91 (m, 1H, H-3), 3.67–3.59 (m, 2H, H-6), 3.38 (dd, 1H, *J*_4,3_ = 8.8 Hz, *J*_4,5_ = 8.8 Hz, H-4), 2.93–2.90 (m, 1H, H-5), 2.83 (dd, 1H, *J*_2,1_ = 9.0 Hz, *J*_2,2′_ 4.4 Hz, H-2), 2.18–2.13 (m, 1H, H-2′) ppm. ^13^C NMR (125 MHz, CD_3_OD) *δ* 142.90, 141.41, 133.43, 123.42, 122.82, 118.63, 112.75, 80.59, 74.64, 70.85, 68.77, 60.73, 34.06 ppm. HR-MALDI-Tof/MS: *m/z* calculated for C_13_H_16_N_2_O_4_ 264.1110; found 265.1188 [M + H]^+^.

*N-[3,4,6-tri-O-benzyl-2-deoxy-β-d-glucopyranosyl]-1H-4-phenylimidazole* (**13**). The crude mixture (0.51 g) was purified by flash column chromatography on silica gel (hexane/ethyl acetate, 3:1 *v/v*) to give a white oil (0.32 g, 70%). ^1^H NMR (400 MHz, CDCl_3_) *δ* 7.77 (d, 1H, *J* = 7.6 Hz, Ar), 7.68 (s, 1H, NC*H*N), 7.37–7.31 (m, 20H, Ar), 5.26 (1H, *J*_1,2_ = 10 Hz, H-1), 4.93 (d, 1H, J = 10.8 Hz, C*H*_2_Ph), 4.74–4.50 (m, 5H, C*H*_2_Ph), 3.81–3.75 (m, 3H, H-6, H-4, H-3), 3.68 (dd, 1H, *J*_5,4_ = 9.2 Hz, *J*_5,6_ = 8.8 Hz, H-5), 3.63–3.60 (m, 1H, H-6), 2.54 (dd, 1H, *J*_1,2_ = 7.6 Hz, *J*_2′,2_ = 6.2 Hz, H-2′), 2.13 (dd, 1H, *J*_1,2_ = 12.0 Hz, *J*_2,2′_ = 11.6 Hz, H-2) ppm. ^13^C NMR (125 MHz, CDCl_3_) *δ* 141, 136.7, 134.76, 127.4, 127.36, 127.27, 127.23, 126.89, 126.74, 126.70, 126.64, 126.54, 126.51, 123.70, 111.52, 80.82, 78.11, 76.61, 75.86, 74.03, 72.35, 70.72, 67.54, 35.80 ppm. HR-MALDI-Tof/MS: *m/z* calculated for C_36_H_36_N_2_O_4_ 560.2675; found 561.2753 [M + H]^+^.

*N-[3,4,6-tri-O-benzyl-2-deoxy-β-d-glucopyranosyl]-1H-5-(4-bromophenyl)-3H-imidazole* (**14**). The crude mixture (0.64 g) was purified by flash column chromatography on silica gel (hexane/ethyl acetate, 3:1 then 1:1 *v/v*) to give a yellowish oil (0.38 g, 68%). ^1^H NMR (400 MHz, CDCl_3_) *δ* 7.77 (d, 1H, *J* = 10.4 Hz, Ar), 7.37–7.20 (m, 20H, Ar), 5.25 (dd, 1H, *J*_1,2_ = 9.6 Hz, H-1) 4.93 (d, 1H, *J* = 10.0 Hz, C*H*_2_Ph), 4.69 (dd, 2H, *J* = 10.0 Hz, *J* = 9.6 Hz, C*H*_2_Ph), 4.62–4.5 (m, 3H, C*H*_2_Ph), 3.81–3.77 (m, 3H, H-5, H-4, H-3) 3.63–3.60 (m, 2H, H-6), 2.56–2.52 (m, 1H, H-2), 2.15–2.07 (m, 1H, H-2) ppm. ^13^C NMR (125 MHz, CDCl_3_) *δ* 141, 137.98, 136.07, 128.71, 128.67, 128.57, 128.53, 128.20, 128.04, 128.01, 127.96, 127.84, 127.81, 125.01, 112.84, 82.11, 79.39, 75.34, 73.64, 72.01, 68.81, 37.08 ppm. HR-MALDI-Tof/MS: *m/z* calculated for C_36_H_35_BrN_2_O_4_ 638.1780; found 639.1858 [M + H]^+^.

*N-[3,4,6-tri-O-acetyl-2-deoxy-2-iodo-α-d-mannopyranosyl]-1H-benzotriazole* (**21a**). The crude mixture (0.95 g) was purified by flash column chromatography on silica gel (hexane/ethyl acetate, 4:1, *v/v*) to give a clear oil (0.72 g, 76%). ^1^H NMR (400 MHz, acetone-*d*_6_) *δ* 7.97 (d, 1H, *J* = 8 Hz, Ar), 7.83 (d, 1H, *J* = 8.4 Hz, Ar), 7.53 (t, 1H, *J* = 8.3 Hz, *J* = 8.0 Hz, Ar), 7.37 (t, 1H, *J* = 8.1 Hz, *J* = 8.0 Hz, Ar), 6.73 (d, 1H, *J*_1,2_ = 4.4 Hz, H-1), 5.60 (dd, 1H, *J*_2,1_ = 4.4 Hz, *J*_2,3_ = 4.2 Hz, H-2), 5.29 (dd, 1H, *J*_3,2_ = 4.0 Hz, *J*_3,4_ = 3.6 Hz, H-3), 5.23 (dd, 1H, *J*_4,5_ = 7.0 Hz, *J*_4,3_ = 6.8 Hz, H-4), 4.42 (dd, 1H, *J*_6,5_ = 7.2 Hz, *J*_6,6_ = 5.6 Hz, H-6), 4.0 (dd, 1H, *J*_6,5_ = 10 Hz, *J*_6,6_ = 6.4 Hz, H-6), 3.81–3.76 (m, 1H, H-5), 2.04–1.83 (m, 9H, C*H*_3_) ppm. ^13^C NMR (125 MHz, acetone-*d*_6_) *δ* 169.91, 169.27, 168.86, 146.04, 132.70, 128.26, 124.74, 119.72, 111.04, 85.10, 73.40, 70.07, 67.01, 61.04, 25.86, 20.11, 19.92, 19.82 ppm. HR-MALDI-Tof/MS: *m/z* calculated for C_18_H_20_IN_3_O_7_ 517.0346; found 518.0424 [M + H]^+^.

*N-[3,4,6-tri-O-acetyl-2-deoxy-2-iodo-α-d-mannopyranosyl]-2H- benzotriazole* (**21b**). The crude mixture (0.95 g) was purified by flash column chromatography on silica gel (hexane/ethyl acetate, 4:1, *v/v*) to give a clear oil (0.055 g, 5.5%). ^1^H NMR (400 MHz, acetone-*d*_6_) *δ* 7.82 (d, 2H, *J* = 8.8 Hz, Ar), 7.39 (d, 2H, *J* = 9.6 Hz, Ar), 6.67 (d, 1H, *J*_1,2_ = 3.6 Hz, H-1), 5.37 (dd, 1H, *J*_2,3_ = 4.0 Hz, *J*_2,3_ = 3.6 Hz, H-2), 5.31 (dd, 1H, *J*_3,2_ = 4.0 Hz, *J*_3,4_ = 2.0 Hz, H-3), 4.27 (dd, 1H, *J*_6,5_ = 6.4 Hz, *J*_6,6_ = 6.0 Hz, H-6), 4.18 (dd, 1H, *J*_4,5_ = 2.4 Hz, *J*_4,3_ = 2.0 Hz, H-4), 4.07 (dd, 1H, *J*_6,6_ = 9.6Hz, *J*_6,5_ = 2.8 Hz, H-6), 3.91 (dd, 1H, *J*_5,6_ = 7.2 Hz, *J*_5,4_ = 7.2 Hz, H-5), 2.02–1.86 (m, 9H, C*H*_3_) ppm. ^13^C NMR (125 MHz, acetone-*d*_6_) *δ* 169.82, 169.21, 168.9, 144.28, 127.69, 118.58, 91.72, 73.58, 69.78, 67.17, 61.30, 25.35, 20.07, 19.94, 19.81 ppm. HR-MALDI-Tof/MS: *m/z* calculated for C_18_H_20_IN_3_O_7_ 517.0346; found 518.0424 [M + H]^+^.

*N-[4,6-O-benzylidine-3-hydroxyl-2-deoxy-2-iodo-α-d-mannopyranosyl]-1H-benzotriazole* (**22a**) The crude mixture (1.12 g) was purified by flash column chromatography on silica gel (hexane/ethyl acetate, 8:1, *v/v*) to give a white solid (0.87 g, 86%). ^1^H NMR (400 MHz, acetone-*d*_6_) *δ* 7.92 (d, 1H, *J* = 8.4 Hz, Ar), 7.67 (d, 1H, *J* = 8.4 Hz, Ar), 7.48 (t, 1H, *J* = 8.1 Hz, *J* = 7.2 Hz, Ar), 7.36 (t, 1H, *J* = 7.6 Hz, *J* = 7.2 Hz, Ar), 7.28–7.26 (m, 2H, Ar), 7.17–7.11 (m, 3H, Ar), 5.50 (s, 1H, C*H*Ph), 5.48 (dd, 1H, *J*_1,2_ = 4.8 Hz, H-1), 4.08 (dd, 1H, *J*_4,3_ = 9.6 Hz, *J*_4,5_ = 9.2 Hz, H-4), 3.98–3.92 (m, 1H, H-6), 3.88–3.85 (m, 2H, H-3, H-6), 3.78–3.73 (m, 2H, H-6′, H-2), 3.11–3.05 (m, 1H, H-5) ppm. ^13^C NMR (125 MHz, acetone-*d*_6_) *δ* 145.58, 137.43, 128.64, 128.40, 127.69, 126.15, 125.05, 119.03, 111.01, 101.97, 89.18, 80.31, 67.63, 67.04, 66.37, 60.23, 33.17 ppm. HR-MALDI-Tof/MS: *m/z* calculated for C_19_H_18_IN_3_O_4_ 479.0342; found 480.0420 [M + H]^+^.

*N-[4,6-O-benzylidine-3-hydroxyl-2-deoxy-2-iodo-α-d-mannopyranosyl]-2H-benzotriazole* (**22b**). The crude mixture (1.12 g) was purified by flash column chromatography on silica gel (hexane/ethyl acetate, 3:1, *v/v*) to give a yellowish oil (0.10 g, 10%). ^1^H NMR (400 MHz, acetone-*d*_6_) *δ* 7.95–7.93 (m, 2H, Ar), 7.50-7.44 (m, 2H, Ar), 7.43-7.32 (m, 5H, Ar), 5.72 (s, 1H, C*H*Ph), 5.41 (dd, 1H, *J*_1,2_ = 4.8 Hz, H-1), 4.15 (dd, 1H, *J*_4,3_ = 10.8 Hz, *J*_4,5_ = 9.6 Hz, H-4), 4.11–4.09 (m, 1H, H-6), 4.07–4.03 (m, 2H, H-6, H-2), 3.87 (dd, 1H, *J*_3,2_ = 10.0 Hz, *J*_3,4_ = 9.6 Hz, H-3), 3.77–3.75 (m, 1H, H-5) ppm. ^13^C NMR (125 MHz, acetone-*d*_6_) *δ* 145.98, 127.96, 126.47, 124.74, 119.64, 111.28, 101.74, 88.96, 80.79, 67.16, 67.09, 66.50, 59.75, 20.06, 13.70 ppm. HR-MALDI-Tof/MS: *m/z* calculated for C_19_H_18_IN_3_O_4_ 479.0342; found 480.0420 [M + H]^+^.

*N-[3,4,6-tri-O-benzyl-2-deoxy-2-iodo-β-d-glucopyranosyl]-1H-benzoimidazole* (**29**). The crude mixture (0.81 g) was purified by flash column chromatography on silica gel (hexane/ethyl acetate, 3:1, *v/v*) to give a white solid (0.65g, 82%). ^1^H NMR (400 MHz, CDCl_3_) *δ* 7.96 (s, 1H, NC*H*N), 7.75 (d, 1H, *J* = 7.2 Hz, Ar), 7.46 (d, 1H, *J* = 7.2 Hz, Ar), 7.34–7.16 (m, 17H, Ar), 5.63 (d, 1H, *J*_1,2_ = 10 Hz, H-1), 4.95–4.81 (m, 3H, C*H*_2_Ph), 4.63–4.40 (m, 3H, C*H*_2_Ph, H-2), 3.87 (dd, 1H, *J*_3,2_ = 3.6 Hz, *J*_3,4_ = 2.0 Hz, H-3), 3.77–3.65 (m, 4H, H-4, H-5, H-6) ppm. ^13^C NMR (125 MHz, CDCl_3_) *δ* 143.15, 140.85, 136.74, 136.45, 136.26, 127.57, 127.48, 127.38, 127.09, 127.05, 126.89, 126.70, 126.55, 110.52, 86.05, 84.88, 77.89, 77.24, 74.85, 74.25, 72.50, 67.02, 32.0 ppm. HR-MALDI-Tof/MS: *m/z* calculated for C_34_H_33_IN_2_O_4_ 660.1485; found 661.1563 [M + H]^+^.

*N-[3,4,6-tri-O-benzyl-2-deoxy-2-iodo-β-d-glucopyranosyl]-1H-4-phenylimidazole* (**31**). The crude mixture (0.70 g) was purified by flash column chromatography on silica gel (hexane/ethyl acetate, 8:1 *v/v*) to give a white solid (0.56 g, 80%). ^1^H NMR (400 MHz, CDCl_3_) *δ* 7.96 (s, 1H, NC*H*N), 7.75 (d, 1H, *J* = 7.2 Hz, Ar), 7.46 (d, 1H, *J* = 7.2 Hz, Ar), 7.34–7.16 (m, 17H, Ar), 5.63 (d, 1H, *J*_1,2_ = 10 Hz, H-1), 4.95–4.81 (m, 3H, C*H*_2_Ph), 4.63–4.40 (m, 3H, C*H*_2_Ph, H-2), 3.87 (dd, 1H, *J*_3,2_ = 3.6 Hz, *J*_3,4_ = 2.0 Hz, H-3), 3.77–3.65 (m, 4H, H-4, H-5, H-6) ppm. ^13^C NMR (125 MHz, CDCl_3_) *δ* 143.15, 140.85, 136.74, 136.45, 136.26, 127.57, 127.48, 127.38, 127.09, 127.05, 126.89, 126.70, 126.55, 110.52, 86.05, 84.88, 77.89, 77.24, 74.85, 74.25, 72.50, 67.02, 32.0 ppm. HR-MALDI-Tof/MS: *m/z* calculated for C_36_H_35_IN_2_O_4_ 686.1642; found 687.1720 [M + H]^+^.

*N-[3,4,6-tri-O-benzyl-2-deoxy-2-iodo-β-d-glucopyranosyl]-1H-5-(4-bromophenyl)-3H-imidazole* (**32**). The crude mixture (1.01 g) was purified by flash column chromatography on silica gel (hexane/ethyl acetate, 6:1 then 3:1 *v/v*) to give a yellowish oil (0.68 g, 75%). ^1^H NMR (400 MHz, CDCl_3_) *δ* 7.64 (d, 1H, *J* = 8.4 Hz, Ar), 7.42–7.12 (m, 20H, Ar), 5.30 (d, 1H, *J =* 10.4, H-1), 4.92 (d, 1H, *J* = 10.4 Hz, C*H*_2_Ph), 4.84 (d, 1H, *J* = 10.0 Hz, C*H*_2_Ph), 4.75 (d, 1H, *J* = 10.4 Hz, C*H*_2_Ph), 4.54 (d, 1H, *J* = 9.6 Hz, C*H*_2_Ph), 4.45 (dd, 2H, *J* = 10.4 Hz, *J* = 10.0 Hz, C*H*_2_Ph), 4.17 (dd, 1H, *J*_1,2_ = 10.4 Hz, *J*_2,2_ = 10.0 Hz, H-2), 3.80–3.69 (m, 3H, H-3, H-4, H-5), 3.66–3.62 (m, 2H, H-6) ppm. ^13^C NMR (125 MHz, CDCl_3_) *δ* 141.0, 137.20, 131.77, 128.70, 128.61, 128.57, 128.23, 128.11, 128.03, 127.97, 127.91, 126.67, 120.91, 112.15, 87.41, 85.89, 78.80, 78.27, 75.89, 75.32, 73.72, 68.17, 32.0 ppm. HR-MALDI-Tof/MS: *m/z* calculated for C_36_H_34_BrIN_2_O_4_ 764.0747; found 765.0825 [M + H]^+^.

*N-[3,4,6-tri-O-acetyl-2-deoxy-2-iodo-β-d-glucopyranosyl]-1H-benzoimidazole* (**33**). The crude mixture (1.01 g) was purified by flash column chromatography on silica gel (hexane/ethyl acetate, 2:1 *v/v*) to give a clear oil (0.71 g, 74%). ^1^H NMR (400 MHz, CDCl_3_) *δ* 8.15 (s, 1H, NC*H*N), 7.82 (t, 1H, *J* = 5.6 Hz, *J* = 3.2 Hz, Ar), 7.62 (t, 1H, *J* = 6.0 Hz, *J* = 4.1 Hz, Ar), 7.33-7.25 (m, 2H, Ar), 6.21 (d, 1H, *J*_1,2_ = 7.6 Hz, H-1), 5.11 (d, 1H, *J*_2,1_ = 6.6 Hz, H-2), 4.82 (t, 1H, *J*_3,2_ = 8.4 Hz, *J*_3,4_ = 3.6 Hz, H-3), 4.14–4.13 (m, 1H, H-6), 4.04 (dd, 1H, *J*_4,3_ = 7.6 Hz, *J*_4,5_ = 3.2 Hz, H-4), 3.68–3.66 (m, 1H, H-5), 2.25–2.00 (s, 12H, CH_3_) ppm. ^13^C NMR (125 MHz, CDCl_3_) *δ* 170.84, 170.60, 169.61, 169.55, 169.28, 144.26, 144.18, 141.79, 141.63, 132.16, 123.84, 123.35, 120.90, 111.69, 111.14, 86.71, 70.83, 66.69,61.78, 60.51, 59.91, 32.0, 26.97, 24.67, 21.09, 20.83, 20.70, 14.29 ppm. HR-MALDI-Tof/MS: *m/z* calculated for C_19_H_21_IN_2_O_7_ 516.0393; found 517.0472 [M + H]^+^.

*N-[3,4,6-tri-O-acetyl-2-deoxy-β-d-glucopyranosyl]-1H-benzoimidazole* (**34**). The crude mixture (0.81 g) was purified by flash column chromatography on silica gel (hexane/ethyl acetate, 2:1 *v/v*) to give a white solid (0.69 g, 71%). ^1^H NMR (400 MHz, CDCl_3_) *δ* 8.23 (s, 1H, NC*H*N), 7.77 (t, 1H, *J* = 6.4 Hz, *J* = 2.4 Hz, Ar), 7.60 (t, 1H, *J* = 6.4 Hz, *J* = 3.0 Hz, Ar), 7.28–7.20 (m, 2H, Ar), 6.0 (d, 1H, *J*_1,2_ = 4.8 Hz, H-1), 5.23 (m, 2H, H-3, H-5), 4.23 (dd, 1H, *J*_6_ = 9.2 Hz, *J*_6,6′_ = 5.2 Hz, H-6), 3.91 (dd, 1H, *J*_6,5_ = 10.4 Hz, *J*_6,6′_ = 2.0 Hz, H-6′), 3.44–3.45 (m, 1H, H-4), 3.03 (dd, 1H, *J*_2,1_ = 4.0 Hz, *J*_2,2′_ = 3.2 Hz, H-2), 2.34–2.28 (m, 1H, H-2′), 2.06–1.96 (s, 9H, C*H*_3_) ppm. ^13^C NMR (125 MHz, CDCl_3_) *δ* 170.68, 170.63, 169.70, 144.26, 140.94, 133.35, 123.86, 123.31, 120.61, 112.11, 79.71, 70.12, 69.52, 67.66, 61.60, 31.75, 21.01, 20.82, 20.77 ppm. HR-MALDI-Tof/MS: *m/z* calculated for C_19_H_22_N_2_O_7_ 390.1427; found 391.1505 [M + H]^+^.

### 3.2. Biology

HeLa cells were seeded in 96-well plates and left to grow overnight to approximately 80% confluence. The following day, cells were treated with either the DMSO control (represented as a black dashed line in Figure 2 or light grey bars in Figure 3) or the synthesized compounds at a concentration of 100 µM (black bars) or 10 µM (grey bars). Cell viability was determined relative to the DMSO control and is represented by the dashed black line (100% cell viability).

Cell Viability Assays were conducted as follows. All cells were seeded in 96-well plates and were grown to approximately 80% confluence before adding the compounds tested. Compounds were solubilized and compared to dimethyl sulfoxide (DMSO; Sigma Aldrich, St. Louis, MO). Viability assays were performed in duplicate wells using 3-(4,5-dimethylthiazol-2-Yl)-2,5-diphenyltetrazolium bromide (MTT) assay (Thermo Fisher Scientific, Waltham, MA). A 5 mg/mL of MTT solution was made in phosphate buffered saline (PBS), filter sterilized and stored at −20 °C until used. On the day of assay, 20 µL of MTT stock solution was added to cells growing in 100 µL of pre-existing media and allowed to incubate for 2 h to allow for the uptake of the MTT dye. Following this, the media containing MTT was removed and the cells were gently washed once with 100 µL pre-warmed PBS. In order to release the internalized dye, 50 µL of DMSO was added to each well and incubated for 10 min on an orbital shaker for 10 min. Absorbance of the solubilized dye was measured and recorded at 540 nm using a multiplate reader.

All statistical analyses were performed using Graphpad Prism 9.1.1. The significance of difference (*p* value) of pooled results was determined by one-way analysis of variance (ANOVA) followed by the post hoc Dunnett’s tests after three independent trials. Significance was defined according to the scale ** = *p* < 0.05 or *** = *p* < 0.001.

## 4. Conclusions

In summary, we report the synthesis of a series of 2-deoxy-2-iodo-α-d-mannopyranosylbenzotriazoles using the benzyl, 4,6-benzylidene and acetyl protected d-glucal in the presence of *N*-iodosuccinimide (NIS). The best yields were obtained when the reaction was refluxed for 2 h. During the addition reaction, the benzyl protected d-glucal produced the single 1*H*-benzotriazole isomer while a mixture of the 1*H* and 2*H*-benzotriazole isomers was obtained for the acetyl and 4,6-benzylidine protected d-glucal. Removal of the iodine at the C-2 position under free radical conditions afforded the 2-deoxy-d-glucopyranosylbenzotriazoles. The addition of various substituted imidazoles to the benzyl protected d-glucal in the presence of NIS was also accomplished, resulting in the chemical synthesis of a series of 2-deoxy-2-iodo-β-d-glucopyranosylimidazoles. Access to the 2-deoxy-d-glucopyranosylimidazoles was similarly achieved relative to the benzotriazole series. All prepared 2-deoxy sugars were screened for their anticancer activity using four established human cell lines. Our results strongly suggest that both 2-deoxy-α-d-glucopyranosylbenzotriazoles, **5** and **6b**, are cytotoxic to the cervical, lung and breast cancer cell lines tested against in this study. Compound **5** showed an average of less than 5% survival rate at 20 µM concentrations, while the acetyl protected compound **6b** was less than 24% for all four cell lines. The IC_50_ was found to be 2.908 µM and 9.940 µM for compounds **5** and **6b**, respectively. When examining the nature of the different protecting groups for the 1*H*-2-deoxy-d-glucopyranosylbenzotriazole series, the more lipophilic benzyl ethers outperformed the acetyl and 4,6-benzylidene counterparts in cell viability studies. This pattern was also observed for the glucose derivatives **1–4** when compared to compound **I**. For the 2-deoxy-*N*-glycoimidazole series, only the benzimidazole containing compound **10** showed potency at 100 µM concentration in our cell viability studies. The cytotoxic screening of compounds **5** and **6b** using a broader cell panel is currently underway, as is their optimization and an investigation into their mode of action, with results forthcoming in future publications.

## Supplementary Materials

The following are available online. Copies of ^1^H and ^13^C NMR spectra and HRMS spectra of all reported compounds.
