# Supplementary material for: Synthesis and Antiproliferative Evaluation of 2-Deoxy-N-glycosylbenzotriazoles/imidazoles
Source: Molecules. 2021 Jun 19;26(12):3742. doi: 10.3390/molecules26123742 (PMC8234545; doi:10.3390/molecules26123742)
Supplement: Supplementary file 1 [file molecules-26-03742-s001.zip › molecules-1247464-supplementary.pdf]

Synthesis and Anti-Proliferative Evaluation of 2-deoxy-*N*-glycosylbenzotriazoles/imidazoles

Caleigh S. Garton [1], Noelle K. DeRose [1], Dylan Dominguez [1], Maria L. Turbi-Henderson [2], Ashley L. Lehr [1], Ashley D. Padilla [2], Scott D. Twining [1], Stephanie Casas [1], Chidozie O. Alozie [1], Azad L. Gucwa [2], Mohammed R. Elshaer [3,*], Michael De Castro [1,*]

1. Department of Chemistry, Farmingdale State College-SUNY, 2350 Broadhollow Rd, Farmingdale, NY 11735, USA; decastm@farmingdale.edu (M.D.C); derosenk@farmingdale.edu (N.K.D.); domida2@farmingdale.edu(D.D.); ashleylehr@yahoo.com (A.L.L.); decastmi@gmail.com (S.D.T.); casasl@farmingdale.edu (S.C.); chidoziealozie92@gmail.com (C.O.A.)
2. Department of Biology, Farmingdale State College-SUNY, 2350 Broadhollow Rd, Farmingdale, NY 11735, USA; gucwaal@farmingdale.edu (A.L.G.); turbihml@farmingdale.edu (M.T.H.); padiad@farmingdale.edu (A.D.P.)
3. Department of Chemistry, Biochemistry and Physics, Fairleigh Dickinson University, Madison, NJ 07940, USA; melshaer@fdu.edu (M.R.E.)

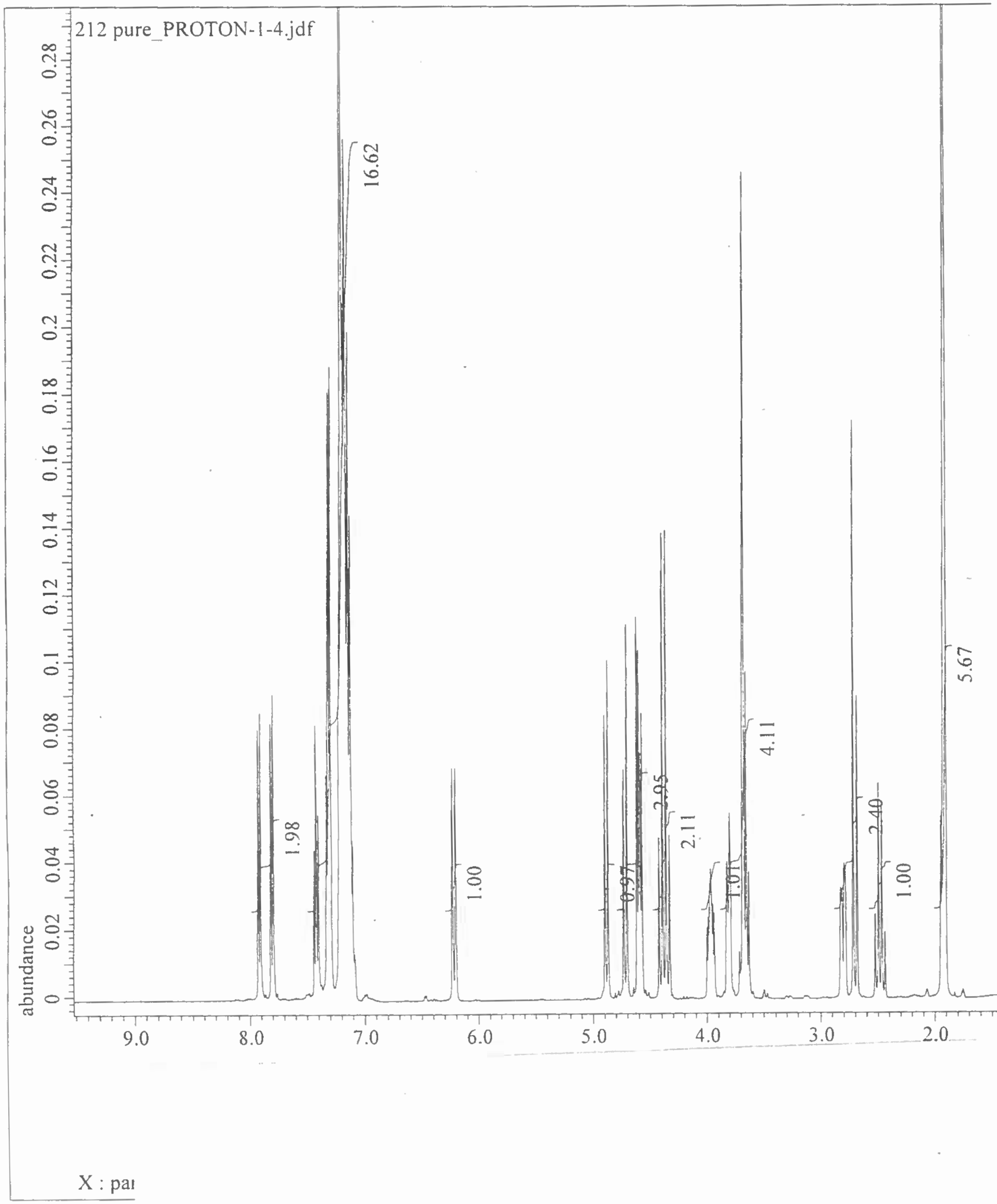

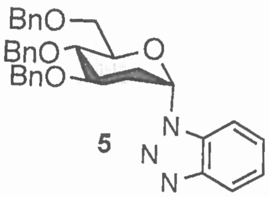

5

```
'ROCESSING PARAMETERS ----
 0.2[Hz], 0.0[s] )
:oid3( 0[%], 80[%], 100[%] )
.11( 1 )
fft( 1, TRUE, TRUE )
machinephase
ppm
thresh( 2[%], 1 )
peak_pick( 0[Hz], 0.1[ppm], Both, 0[Hz] )
norm_smallest_int( 1.0, 0[Hz], 25[Hz] )

Derived from: 212 pure_PROTON-1-1.jdf
```

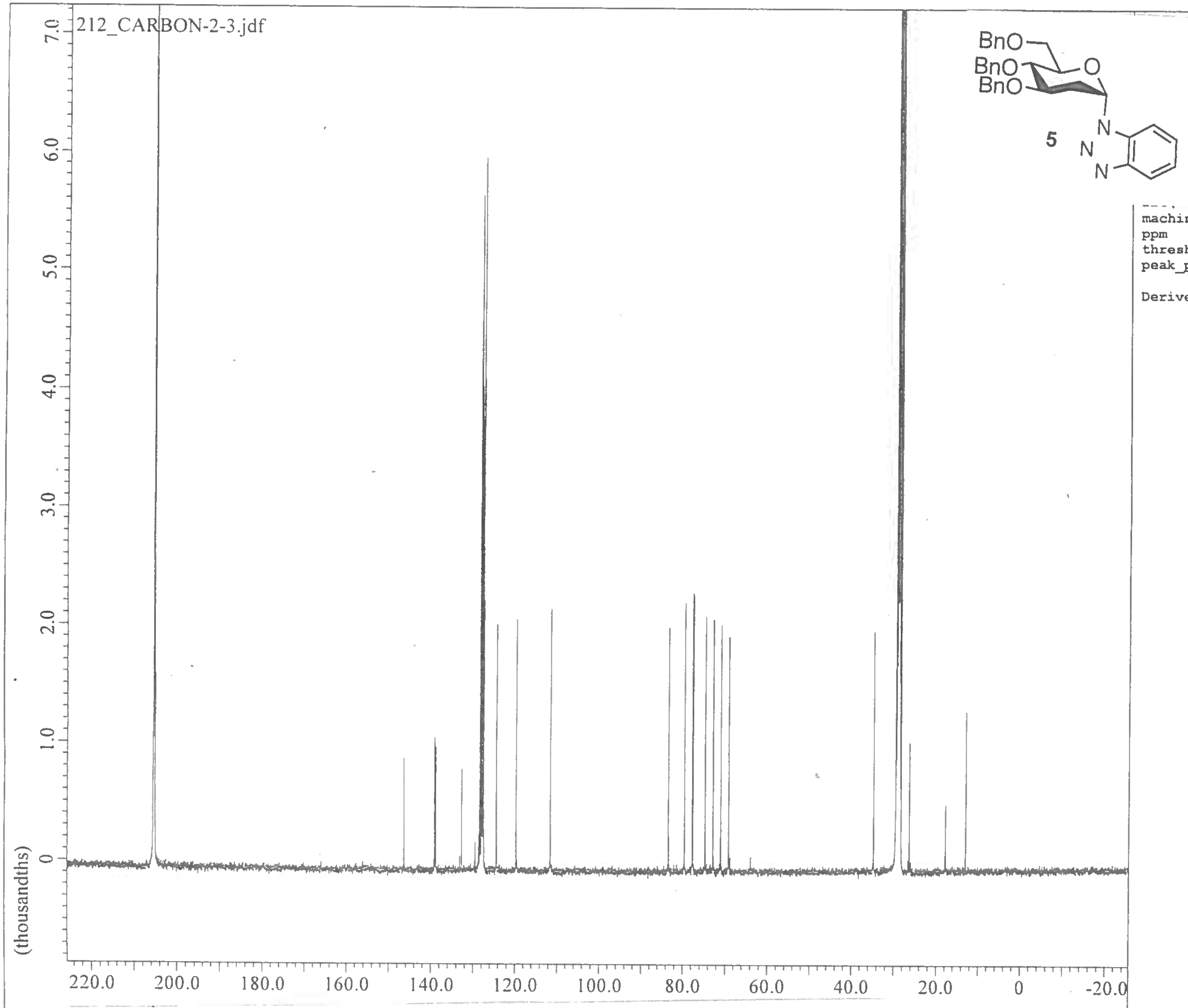

JEOL

CESSING PARAMETERS ----
0[Hz], 0.0[s])
d3(0[%], 80[%], 100[%])
(1)
TRUE, TRUE)
machinephase
ppm
thresh(5[%], 1)
peak_pick(0[Hz], 0.1[ppm], Peaks, 0[Hz])

Derived from: 212_CARBON-2-1.jdf

212

Qtof_68481a 60 (2.285) AM (Cen,3, 80.00, Ar,14000.0,558.36,0.70,LS 5); Sm (SG, 2x3.00); Cm (60:66)

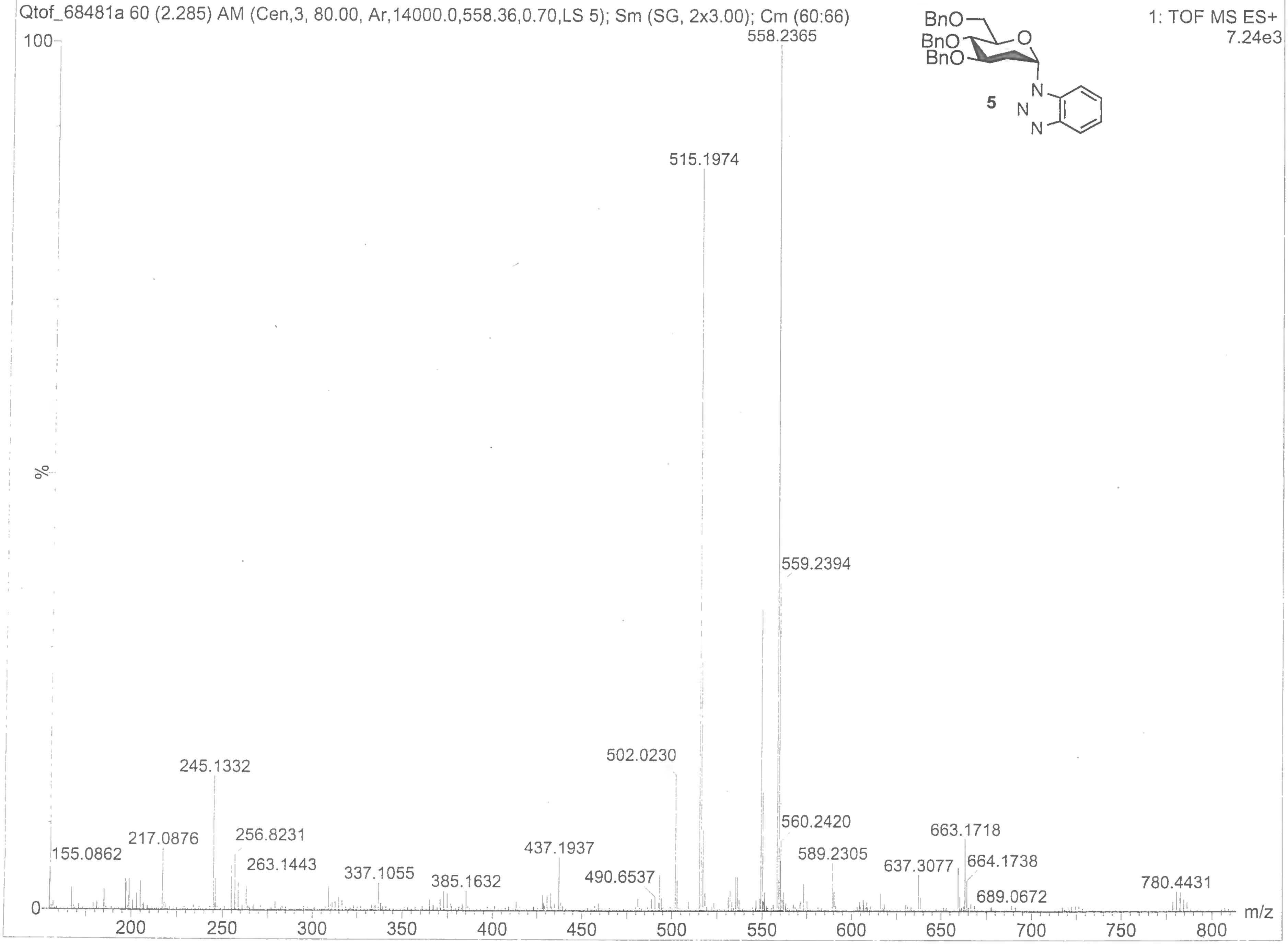

Elemental Composition Report

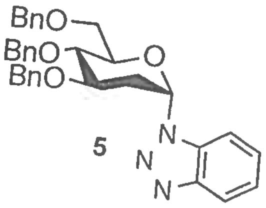

5

Single Mass Analysis

Tolerance = 5.0 PPM / DBE: min = -1.5, max = 100.0
Element prediction: Off
Number of isotope peaks used for i-FIT = 3

Monoisotopic Mass, Even Electron Ions
989 formula(e) evaluated with 5 results within limits (up to 50 closest results for each mass)
Elements Used:
C: 0-200 H: 0-200 N: 0-5 O: 0-6 Na: 0-1 I: 0-1

212
Qtof_68481a 60 (2.285) AM (Cen,3, 80.00, Ar,14000.0,558.36,0.70,LS 5); Sm (SG, 2x3.00); Cm (60:66)

1: TOF MS ES+
7.24e+003

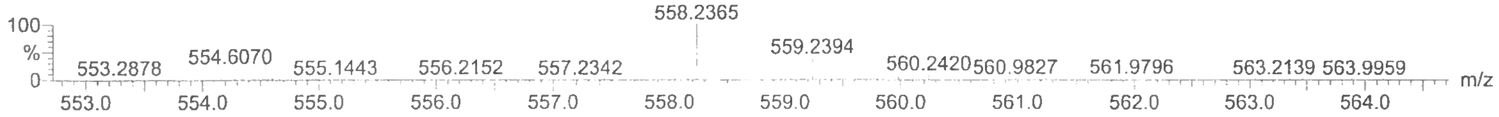

Minimum:				-1.5		
Maximum:		5.0	5.0	100.0		

Mass	Calc. Mass	mDa	PPM	DBE	i-FIT	Formula
558.2365	558.2369	-0.4	-0.7	18.5	0.6	C33 H33 N3 O4 Na
	558.2353	1.2	2.1	17.5	3.4	C30 H32 N5 O6
	558.2380	-1.5	-2.7	-1.5	219.3	C20 H46 N3 O5 Na I
	558.2345	2.0	3.6	10.5	34.2	C29 H41 N3 I
	558.2393	-2.8	-5.0	21.5	8.5	C35 H32 N3 O4

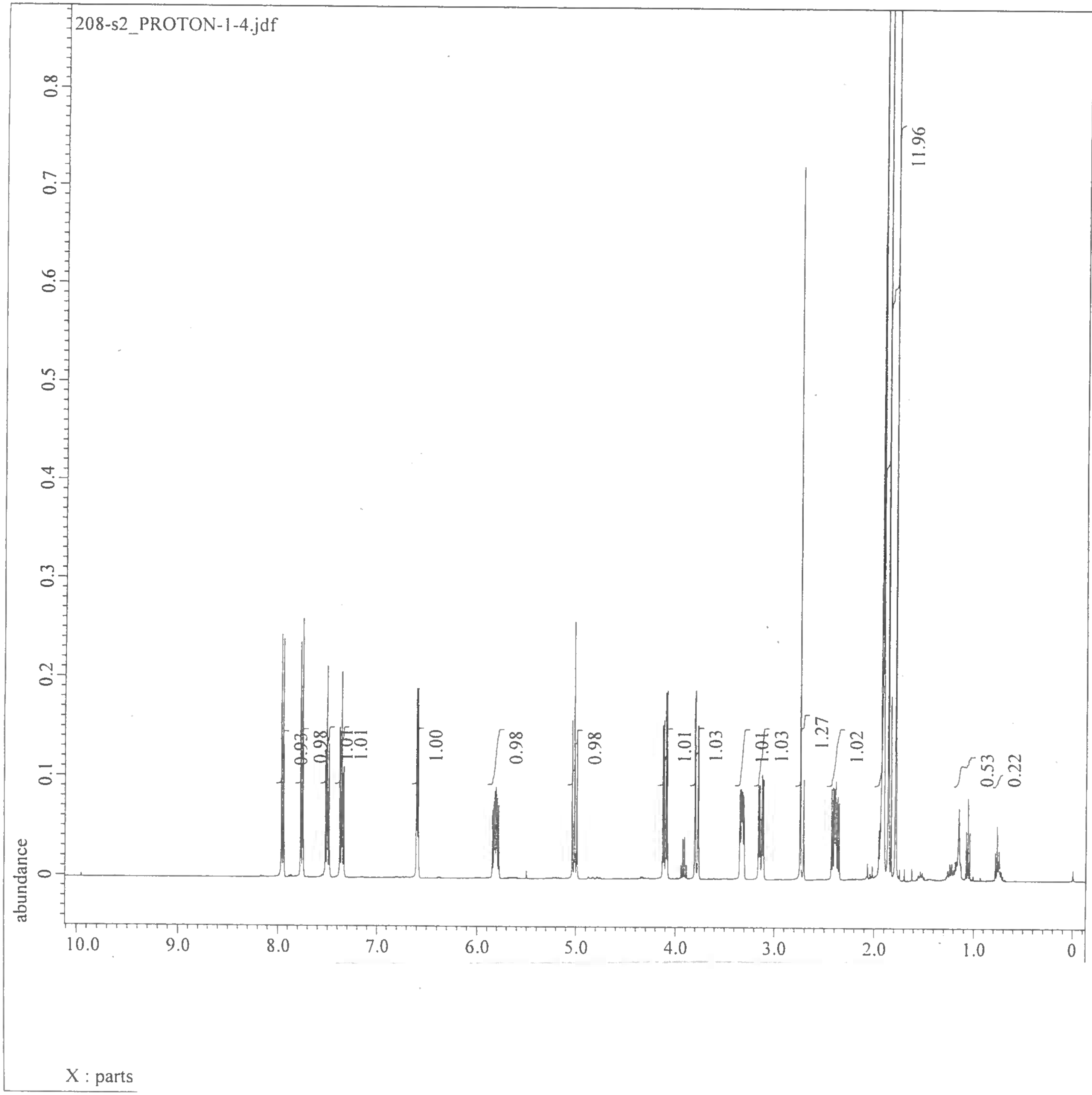

---- PROCESSING PARAMETERS ----
sexp(0.2[Hz], 0.0[s])
trapezoid3(0[%], 80[%], 100[%])
zerofill(1)
fft(1, TRUE, TRUE)
machinephase
ppm
thresh(2[%], 1)
peak_pick(0[Hz], 0.1[ppm], Both, 0[Hz])
norm_smallest_int(1.0, 0[Hz], 25[Hz])

Derived from: 208-s2_PROTON-1-1.jdf

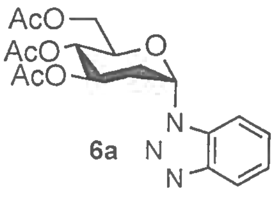

6a

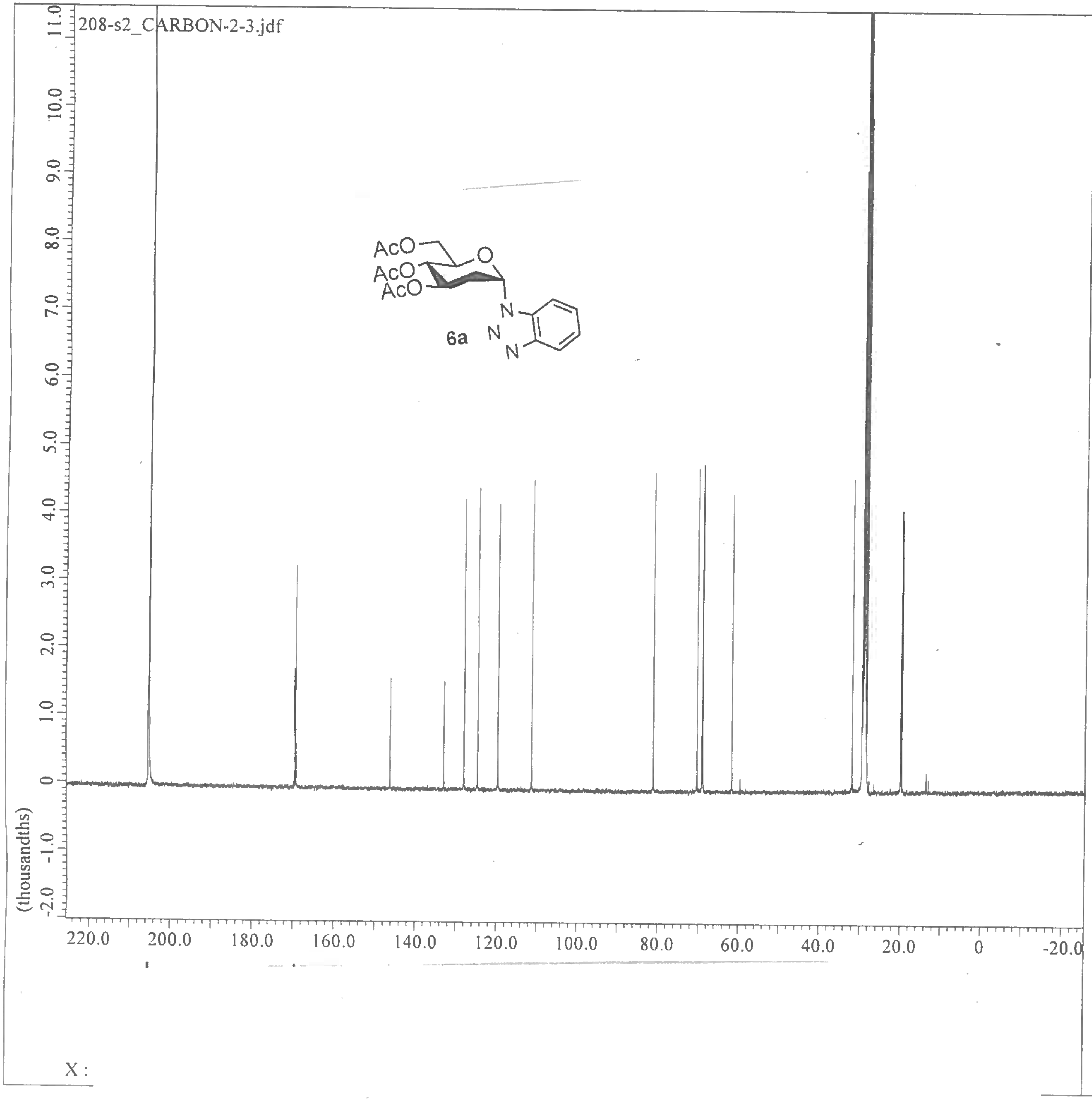

JEOL

```
---- PROCESSING PARAMETERS ----
sexp( 2.0[Hz], 0.0[s] )
trapezoid3( 0[%], 80[%], 100[%] )
zerofill( 1 )
fft( 1, TRUE, TRUE )
machinephase
ppm
thresh( 5[%], 1 )
peak_pick( 0[Hz], 0.1[ppm], Peaks, 0[Hz] )

Derived from: 208-s2_CARBON-2-1.jdf
```

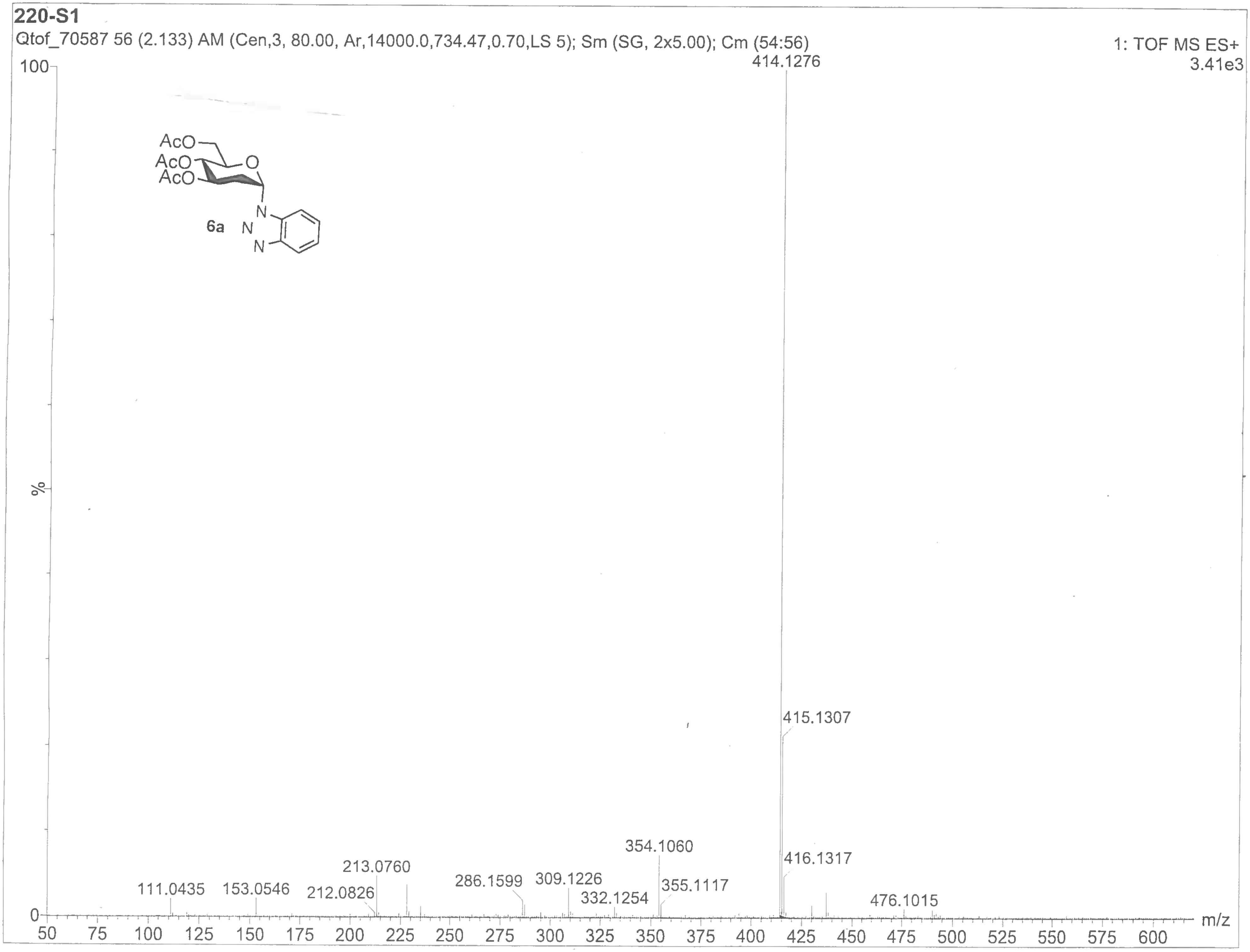
220-S1
Qtof_70587 56 (2.133) AM (Cen,3, 80.00, Ar,14000.0,734.47,0.70,LS 5); Sm (SG, 2x5.00); Cm (54:56)
1: TOF MS ES+
3.41e3
AcO
AcO
AcO
O
N
N
N
6a
414.1276
415.1307
416.1317
476.1015
354.1060
355.1117
332.1254
309.1226
286.1599
213.0760
212.0826
153.0546
111.0435
%
100
0
m/z
50
75
100
125
150
175
200
225
250
275
300
325
350
375
400
425
450
475
500
525
550
575
600

Elemental Composition Report

Single Mass Analysis

Tolerance = 5.0 PPM / DBE: min = -1.5, max = 100.0
Element prediction: Off
Number of isotope peaks used for i-FIT = 3

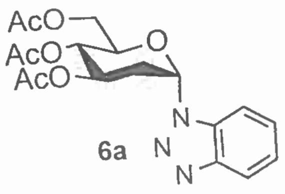

6a

Monoisotopic Mass, Even Electron Ions
310 formula(e) evaluated with 2 results within limits (up to 50 closest results for each mass)
Elements Used:
C: 0-200 H: 0-200 N: 0-5 O: 4-9 Na: 0-1
220-S1
Qtof_70587 56 (2.133) AM (Cen,3, 80.00, Ar,14000.0,734.47,0.70,LS 5); Sm (SG, 2x5.00); Cm (54:56)

1: TOF MS ES+
3.41e+003

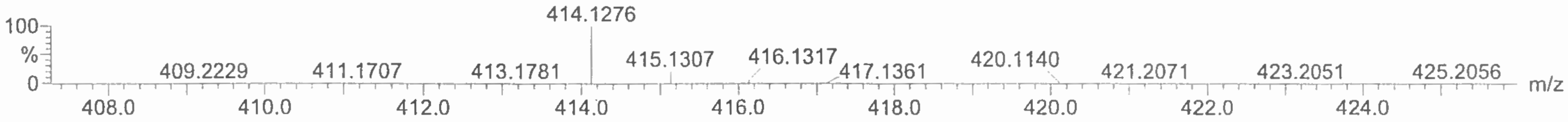

Minimum:				-1.5		
Maximum:		5.0	5.0	100.0		
Mass	Calc. Mass	mDa	PPM	DBE	i-FIT	Formula
414.1276	414.1277	-0.1	-0.2	9.5	4.6	C18 H21 N3 O7 Na
	414.1261	1.5	3.6	8.5	7.9	C15 H20 N5 O9

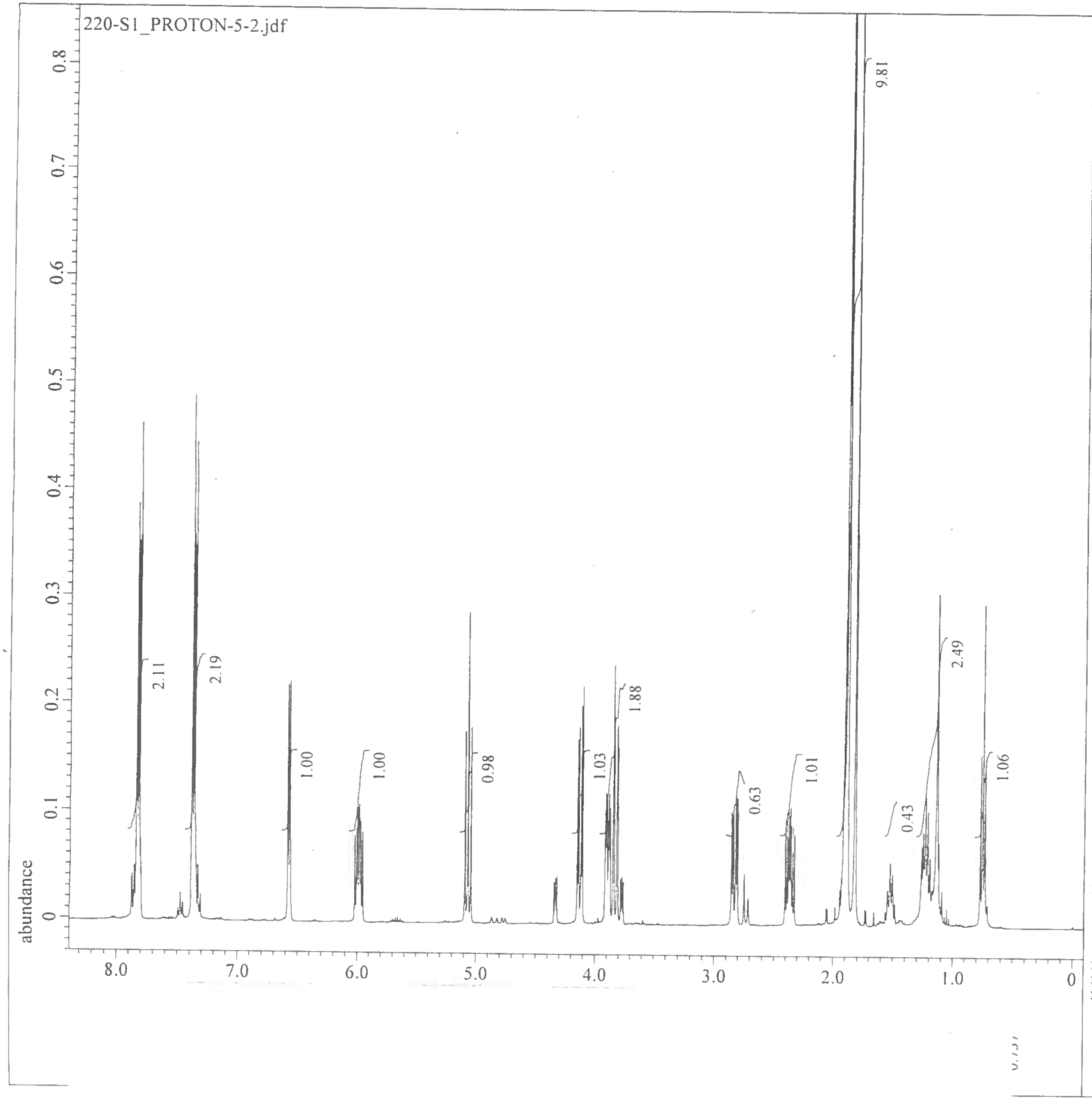

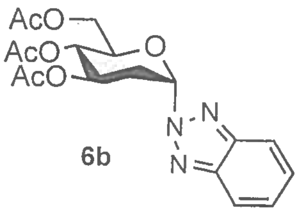

6b

Filename	= 220-S1_PROTON-5-2.j
Author	= decastm
Experiment	= proton.jxp
Sample_Id	= 220-S1
Solvent	= ACETONE-D6
Actual_Start_Time	= 14-NOV-2018 19:05:3
Revision_Time	= 4-JUN-2019 15:56:5
Data_Format	= 1D COMPLEX
Dim_Size	= 13107
X_Domain	= Proton
Dim_Title	= Proton
Dim_Units	= [ppm]
Dimensions	= X
Site	= Farmingdale State C
Spectrometer	= JNM-ECZ400S/L1
Field_Strength	= 9.389766[T] (400[MH
X_Acq_Duration	= 2.18628096[s]
X_Domain	= 1H
X_Freq	= 399.78219838[MHz]
X_Offset	= 5[ppm]
X_Points	= 16384
X_Prescans	= 1
X_Resolution	= 0.45739775[Hz]
X_Sweep	= 7.4940048[kHz]
X_Sweep_Clipped	= 5.99520384[kHz]
Irr_Domain	= Proton
Irr_Freq	= 399.78219838[MHz]
Irr_Offset	= 5[ppm]
Tri_Domain	= Proton
Tri_Freq	= 399.78219838[MHz]
Tri_Offset	= 5[ppm]
Blanking	= 2[us]
Clipped	= FALSE
Scans	= 16
Total_Scans	= 16
Relaxation_Delay	= 4[s]
Recvr_Gain	= 36
Temp_Get	= 18.9[dC]
X_90_Width	= 5.894[us]
X_Acq_Time	= 2.18628096[s]
X_Angle	= 45[deg]
X_Atn	= 1.5[dB]
X_Pulse	= 2.947[us]
Irr_Mode	= Off
Tri_Mode	= Off
Dante_Loop	= 400
Dante_Presat	= FALSE
Decimation_Rate	= 0
Initial_Wait	= 1[s]
Phase	= {0, 90, 270, 180, 1
Presat_Time	= 4[s]
Presat_Time_Flag	= FALSE
Relaxation_Delay_Calc	= 0[s]
Relaxation_Delay_Temp	= 4[s]
Repetition_Time	= 6.18628096[s]

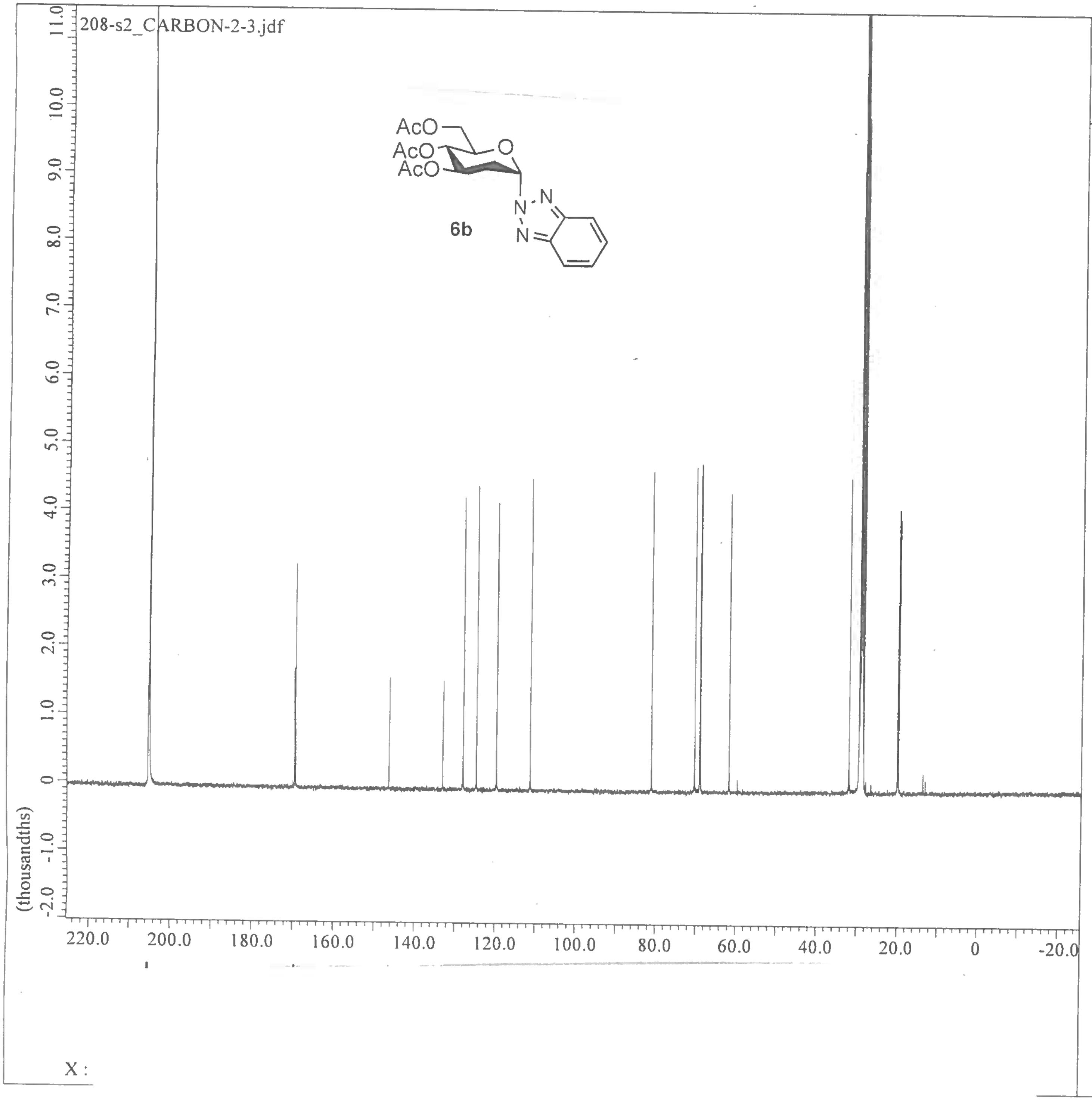

JEOL

```
---- PROCESSING PARAMETERS ----
sexp( 2.0[Hz], 0.0[s] )
trapezoid3( 0[%], 80[%], 100[%] )
zerofill( 1 )
fft( 1, TRUE, TRUE )
machinephase
ppm
thresh( 5[%], 1 )
peak_pick( 0[Hz], 0.1[ppm], Peaks, 0[Hz] )

Derived from: 208-s2_CARBON-2-1.jdf
```

220-S1

Qtof_70587 56 (2.133) AM (Cen,3, 80.00, Ar,14000.0,734.47,0.70,LS 5); Sm (SG, 2x5.00); Cm (54:56)

1: TOF MS ES+
3.41e3

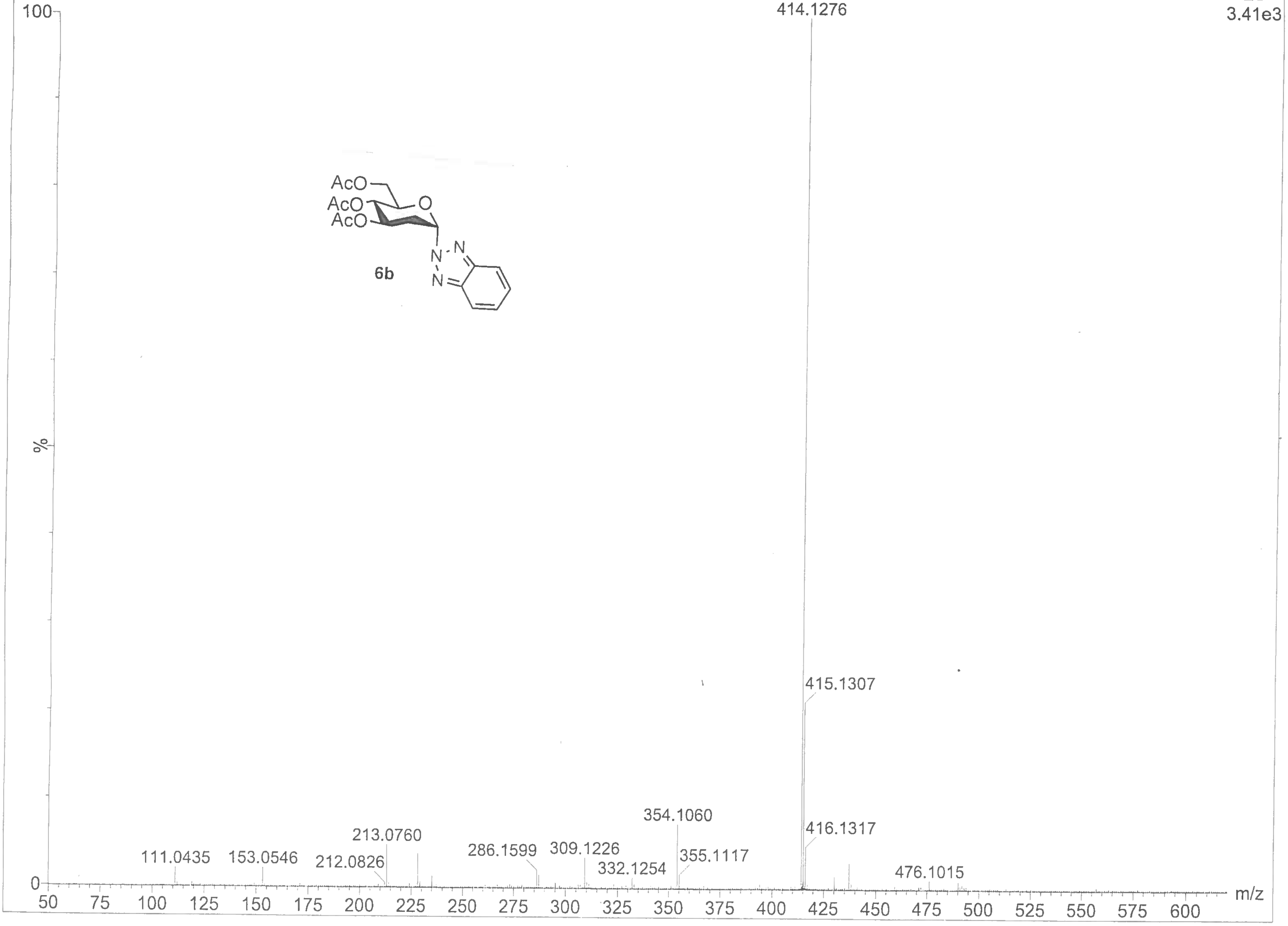

Elemental Composition Report

Single Mass Analysis

Tolerance = 5.0 PPM / DBE: min = -1.5, max = 100.0
Element prediction: Off
Number of isotope peaks used for i-FIT = 3

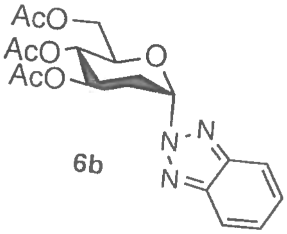

6b

Monoisotopic Mass, Even Electron Ions
310 formula(e) evaluated with 2 results within limits (up to 50 closest results for each mass)
Elements Used:
C: 0-200 H: 0-200 N: 0-5 O: 4-9 Na: 0-1
220-S1
Qtof_70587 56 (2.133) AM (Cen,3, 80.00, Ar,14000.0,734.47,0.70,LS 5); Sm (SG, 2x5.00); Cm (54:56)

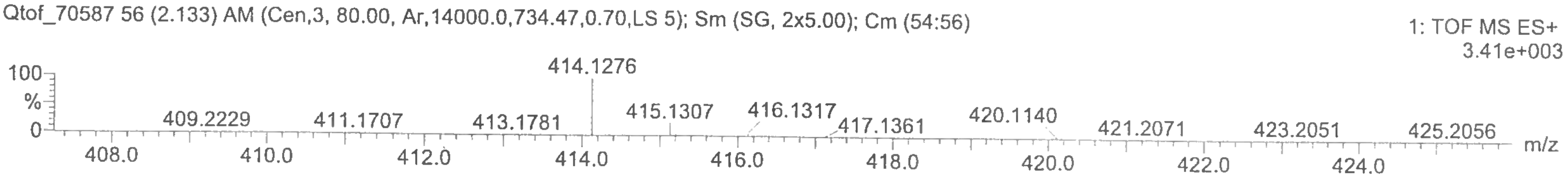

Minimum:				-1.5		
Maximum:		5.0	5.0	100.0		
Mass	Calc. Mass	mDa	PPM	DBE	i-FIT	Formula
414.1276	414.1277	-0.1	-0.2	9.5	4.6	C18 H21 N3 O7 Na
	414.1261	1.5	3.6	8.5	7.9	C15 H20 N5 O9

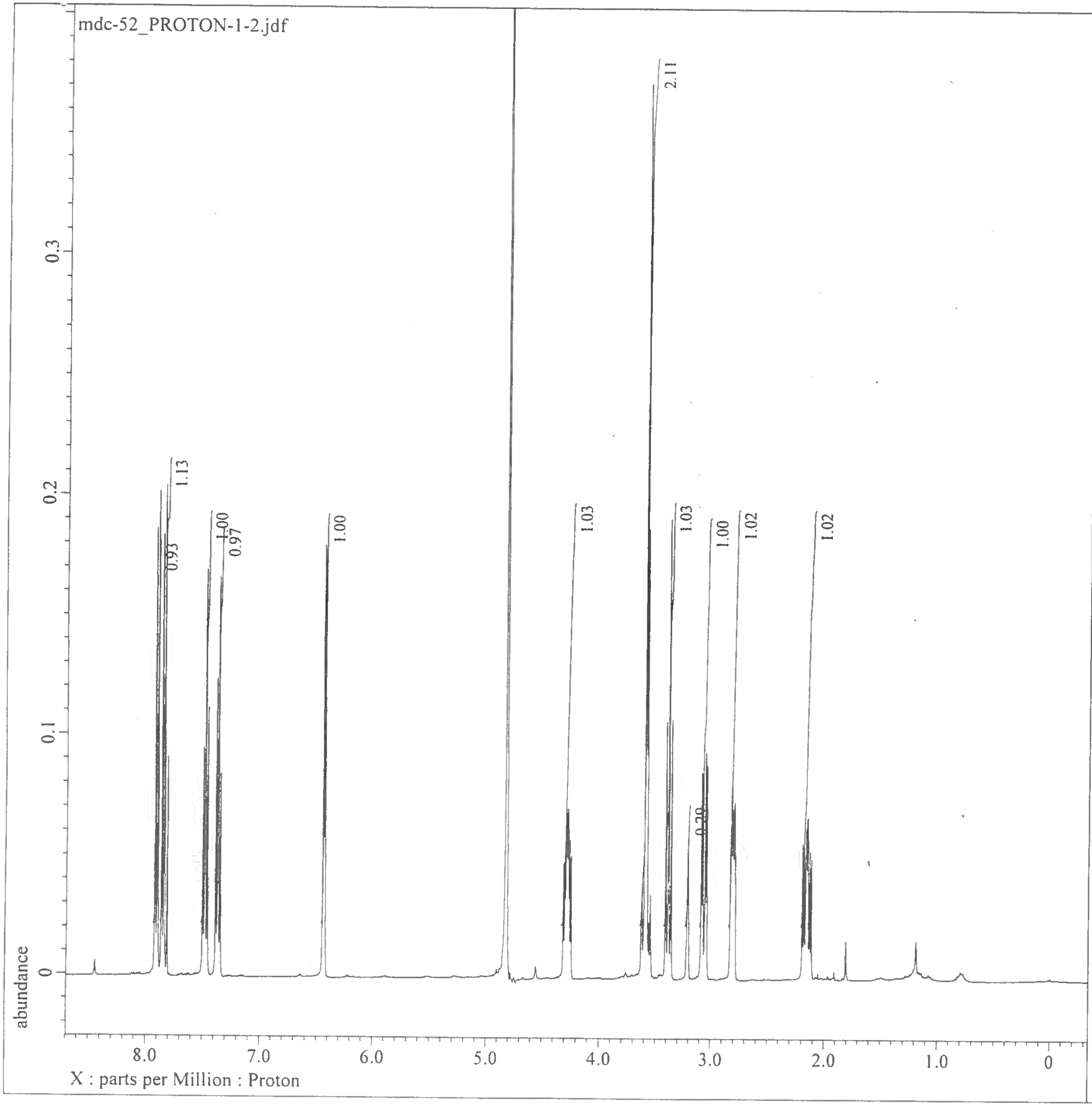

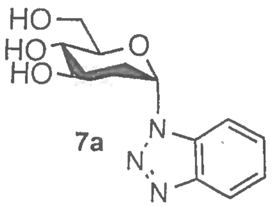

7a

Filename	= mdc-52_PROTON-1-2.j
Author	= decastm
Experiment	= proton.jxp
Sample_Id	= mdc-52
Solvent	= METHANOL-D4
Actual_Start_Time	= 9-JUL-2019 15:19:0
Revision_Time	= 9-JUL-2019 15:21:5
Data_Format	= 1D COMPLEX
Dim_Size	= 26214
X_Domain	= Proton
Dim_Title	= Proton
Dim_Units	= [ppm]
Dimensions	= X
Site	= Farmingdale State C
Spectrometer	= JNM-ECZ400S/L1
Field_Strength	= 9.389766[T] (400[MH
X_Acq_Duration	= 4.37256192[s]
X_Domain	= Proton
X_Freq	= 399.78219838[MHz]
X_Offset	= 5[ppm]
X_Points	= 32768
X_Prescans	= 0
X_Resolution	= 0.22869888[Hz]
X_Sweep	= 7.4940048[kHz]
X_Sweep_Clipped	= 5.99520384[kHz]
Irr_Domain	= Proton
Irr_Freq	= 399.78219838[MHz]
Irr_Offset	= 5[ppm]
Tri_Domain	= Proton
Tri_Freq	= 399.78219838[MHz]
Tri_Offset	= 5[ppm]
Blanking	= 2[us]
Clipped	= FALSE
Scans	= 16
Total_Scans	= 16
Relaxation_Delay	= 4[s]
Recvr_Gain	= 52
Temp_Get	= 17.2[dC]
X_90_Width	= 5.85[us]
X_Acq_Time	= 4.37256192[s]
X_Angle	= 45[deg]
X_Atn	= 1.3[dB]
X_Pulse	= 2.925[us]
Irr_Mode	= Off
Tri_Mode	= Off
Dante_Loop	= 400
Dante_Presat	= FALSE
Decimation_Rate	= 0
Experiment_Path	= c:\Program Files\JE
Initial_Wait	= 1[s]
Phase	= {0, 90, 270, 180, 1
Presat_Time	= 4[s]
Presat_Time_Flag	= FALSE
Relaxation_Delay_Calc	= 0[s]
Relaxation_Delay_Temp	= 4[s]
Repetition_Time	= 8.37256192[s]

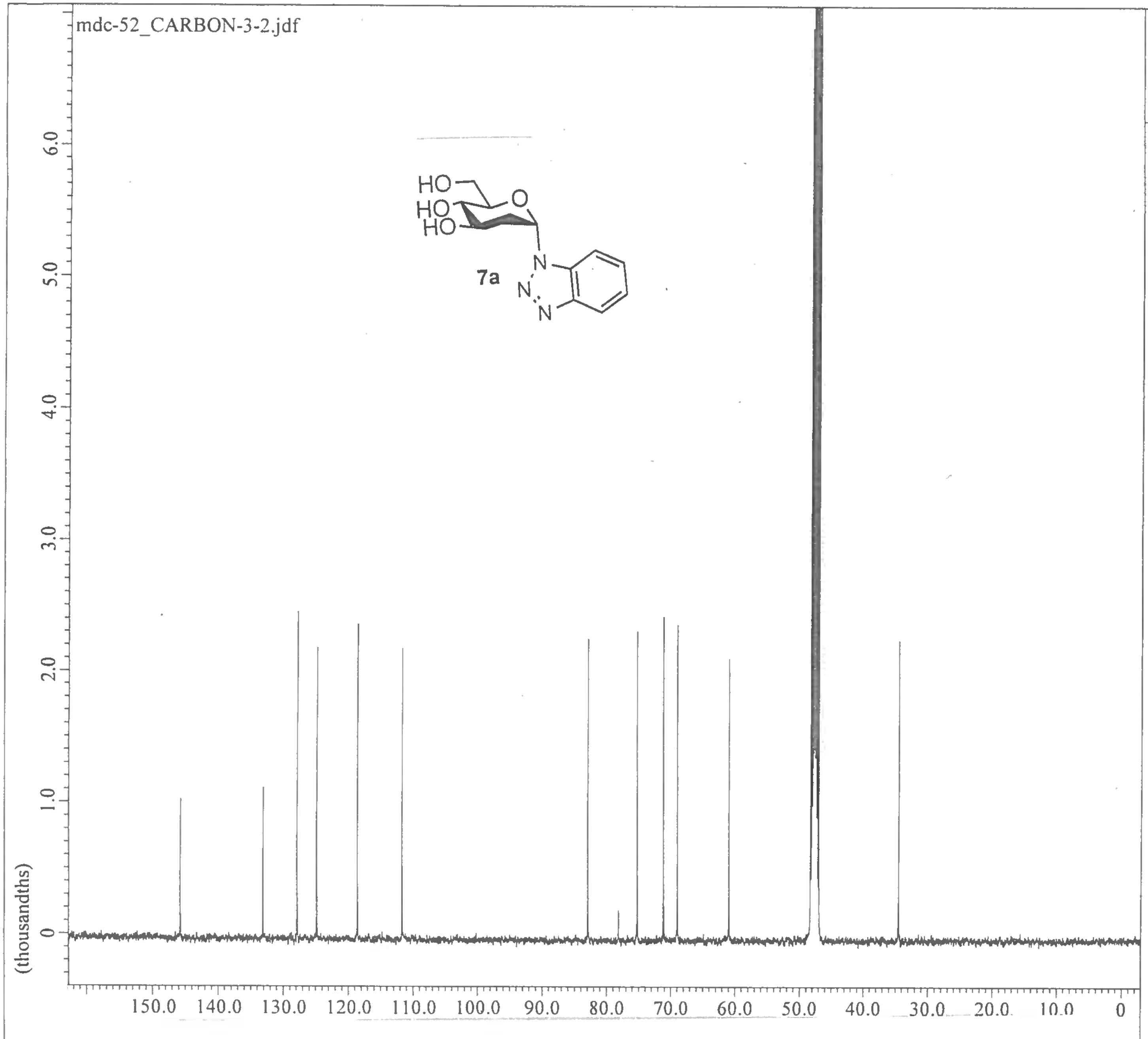

JEOL

Filename	= mdc-52_CARBON-3-
Author	= decastm
Experiment	= carbon.jxp
Sample_Id	= mdc-52
Solvent	= METHANOL-D4
Actual_Start_Time	= 9-JUL-2019 15:2
Revision_Time	= 10-JUL-2019 17:2
Data_Format	= 1D COMPLEX
Dim_Size	= 26214
X_Domain	= Carbon13
Dim_Title	= Carbon13
Dim_Units	= [ppm]
Dimensions	= X
Site	= Farmingdale Stat
Spectrometer	= JNM-ECZ400S/L1
Field_Strength	= 9.389766[T] (400
X_Acq_Duration	= 1.03809024[s]
X_Domain	= Carbon13
X_Freq	= 100.52530333[MHz
X_Offset	= 100[ppm]
X_Points	= 32768
X_Prescans	= 4
X_Resolution	= 0.96330739[Hz]
X_Sweep	= 31.56565657[kHz]
X_Sweep_Clipped	= 25.25252525[kHz]
Irr_Domain	= Proton
Irr_Freq	= 399.78219838[MHz
Irr_Offset	= 5[ppm]
Blanking	= 5[us]
Clipped	= FALSE
Scans	= 5000
Total_Scans	= 5000
Relaxation_Delay	= 2[s]
Recvr_Gain	= 52
Temp_Get	= 18[dC]
X_90_Width	= 11.73[us]
X_Acq_Time	= 1.03809024[s]
X_Angle	= 30[deg]
X_Atn	= 7.9[dB]
X_Pulse	= 3.91[us]
Irr_Atn_Dec	= 27[dB]
Irr_Atn_Dec_Calc	= 27[dB]
Irr_Atn_Dec_Default_Calc	= 27[dB]
Irr_Atn_Noe	= 27[dB]
Irr_Dec_Bandwidth_Hz	= 4.7826087[kHz]
Irr_Dec_Bandwidth_Ppm	= 11.96303566[ppm]
Irr_Dec_Freq	= 399.78219838[MHz
Irr_Dec_Merit_Factor	= 2.2
Irr_Decoupling	= TRUE
Irr_Noe	= TRUE
Irr_Noise	= WALTZ
Irr_Offset_Default	= 5[ppm]
Irr_Pwidth	= 0.115[ms]
Irr_Pwidth_Default	= 0.115[ms]
Irr_Pwidth_Default_Calc	= 0.115[ms]
Irr_Pwidth_Templ	= 0.115[ms]
Irr_Wurst	= FALSE
Decimation_Rate	= 0
Experiment_Path	= c:\Program Files
Initial_Wait	= 1[s]
Noe_Time	= 2[s]

Michael De Castro 209**

Synapt_18959 34 (0.691)

SYNAPTG2-Si#UGA305
13:04:56
1: TOF MS ES+
2.92e5

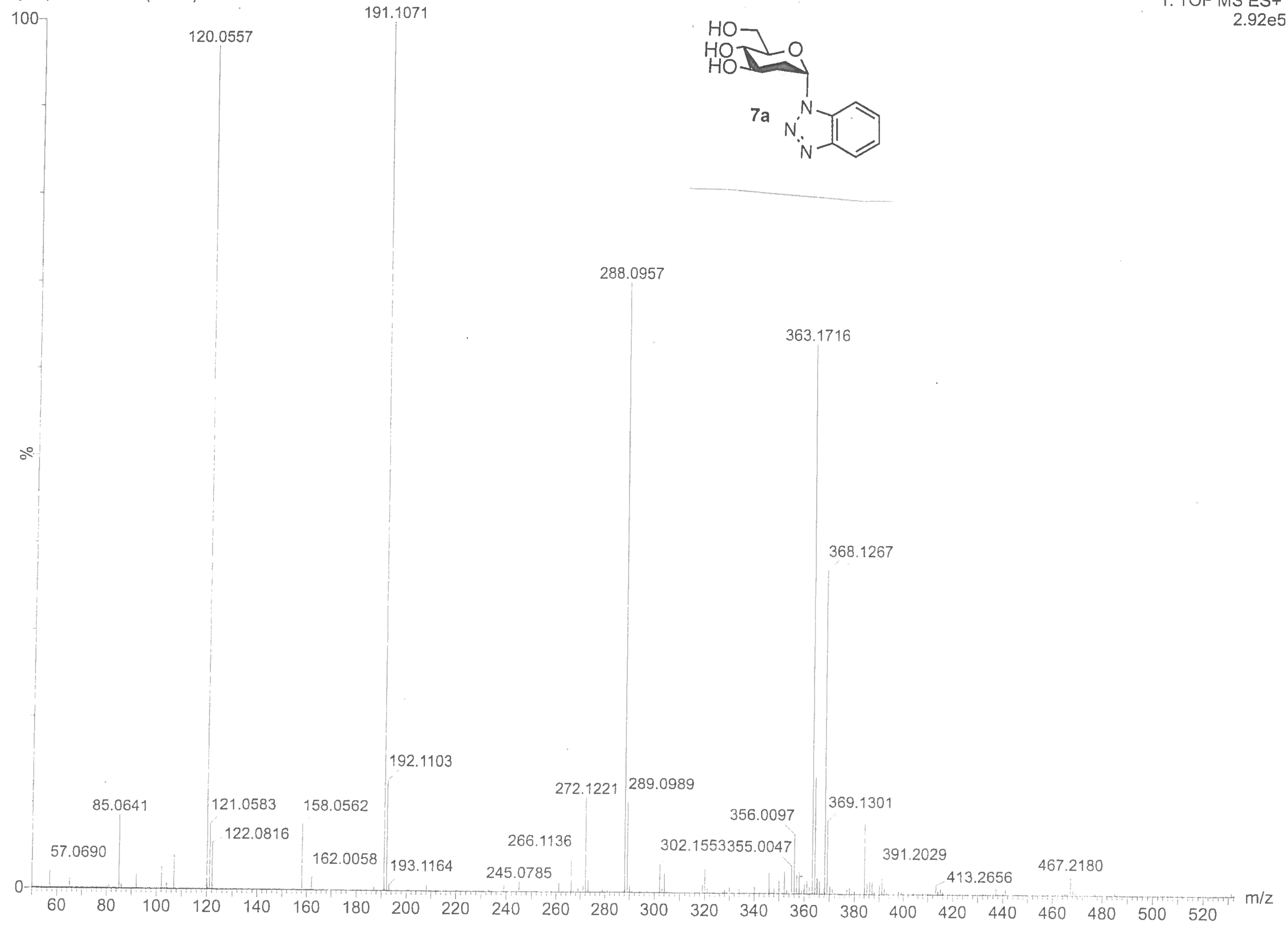

Elemental Composition Report

Single Mass Analysis

Tolerance = 5.0 PPM / DBE: min = -50.0, max = 100.0
Element prediction: Off
Number of isotope peaks used for i-FIT = 9

Monoisotopic Mass, Even Electron Ions
182 formula(e) evaluated with 1 results within limits (up to 10 best isotopic matches for each mass)
Elements Used:
C: 0-90 H: 0-130 N: 2-4 O: 3-5 Na: 0-1

Mass	Calc. Mass	mDa	PPM	DBE	i-FIT	Norm	Conf(%)	Formula
Minimum:				-50.0				
Maximum:		5.0	5.0	100.0				
288.0957	288.0960	-0.3	-1.0	6.5	159.2	n/a	n/a	C12 H15 N3 O4 Na

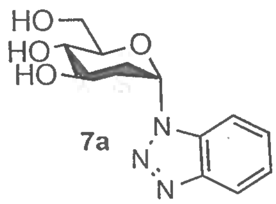

7a

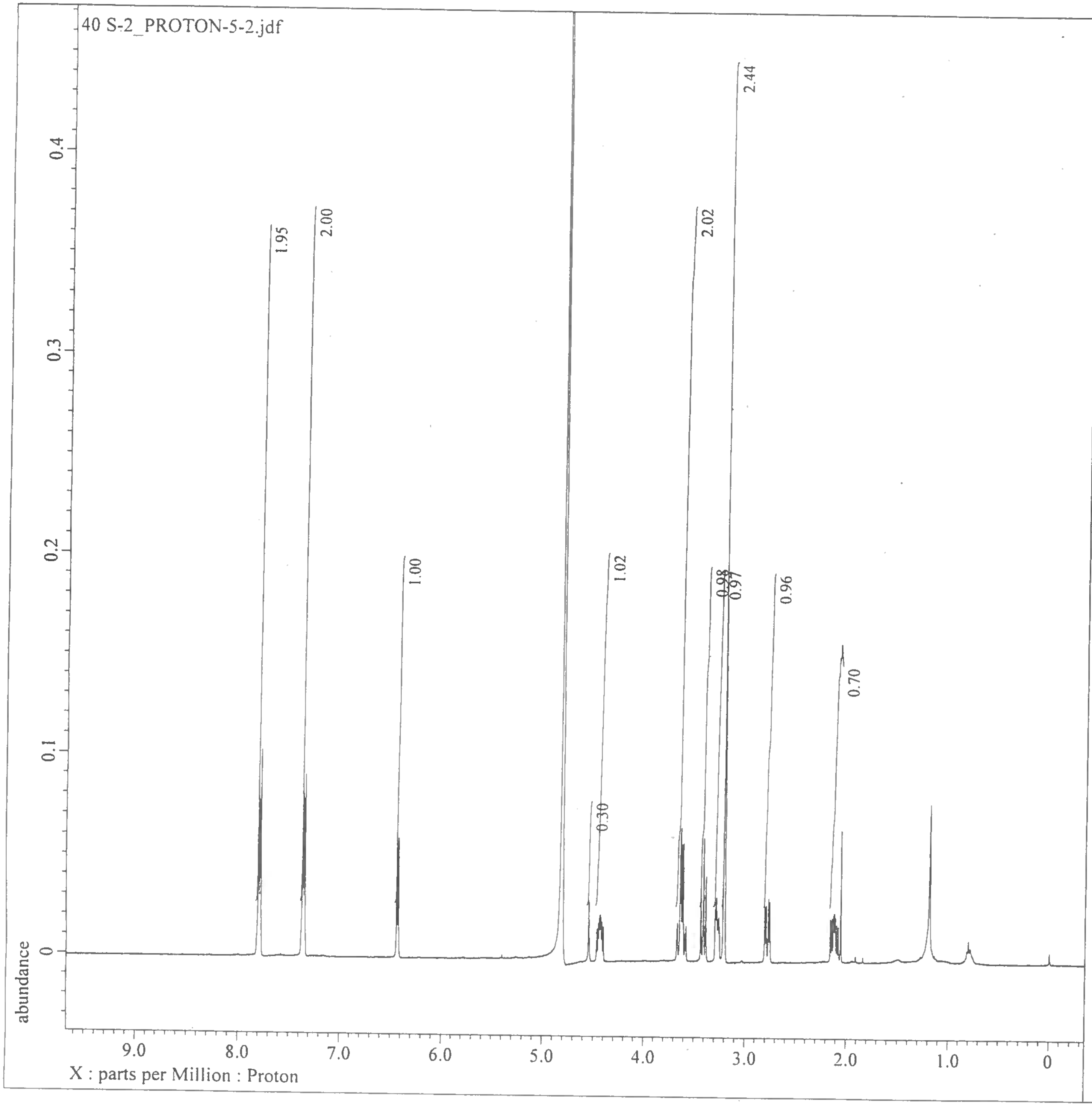

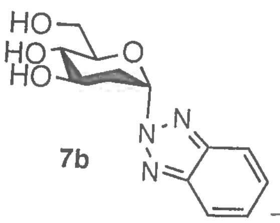

7b

Filename	[illegible]N-5-2.j
Author	
Experiment	= proton.jxp
Sample_Id	= 40 S-2
Solvent	= METHANOL-D4
Actual_Start_Time	= 11-JUN-2019 15:31:4
Revision_Time	= 11-JUN-2019 16:48:3
Data_Format	= 1D COMPLEX
Dim_Size	= 26214
X_Domain	= Proton
Dim_Title	= Proton
Dim_Units	= [ppm]
Dimensions	= X
Site	= Farmingdale State C
Spectrometer	= JNM-ECZ400S/L1
Field_Strength	= 9.389766[T] (400[MH
X_Acq_Duration	= 4.37256192[s]
X_Domain	= Proton
X_Freq	= 399.78219838[MHz]
X_Offset	= 5[ppm]
X_Points	= 32768
X_Prescans	= 0
X_Resolution	= 0.22869888[Hz]
X_Sweep	= 7.4940048[kHz]
X_Sweep_Clipped	= 5.99520384[kHz]
Irr_Domain	= Proton
Irr_Freq	= 399.78219838[MHz]
Irr_Offset	= 5[ppm]
Tri_Domain	= Proton
Tri_Freq	= 399.78219838[MHz]
Tri_Offset	= 5[ppm]
Blanking	= 2[us]
Clipped	= FALSE
Scans	= 16
Total_Scans	= 16
Relaxation_Delay	= 4[s]
Recvr_Gain	= 62
Temp_Get	= 17.5[dC]
X_90_Width	= 5.85[us]
X_Acq_Time	= 4.37256192[s]
X_Angle	= 45[deg]
X_Atn	= 1.3[dB]
X_Pulse	= 2.925[us]
Irr_Mode	= Off
Tri_Mode	= Off
Dante_Loop	= 400
Dante_Presat	= FALSE
Decimation_Rate	= 0
Experiment_Path	= c:\Program Files\JE
Initial_Wait	= 1[s]
Phase	= {0, 90, 270, 180, 1
Presat_Time	= 4[s]
Presat_Time_Flag	= FALSE
Relaxation_Delay_Calc	= 0[s]
Relaxation_Delay_Temp	= 4[s]
Repetition_Time	= 8.37256192[s]

mdc-52_CARBON-3-2.jdf

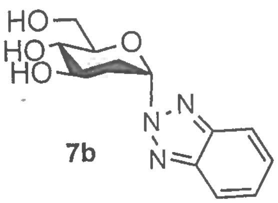

7b

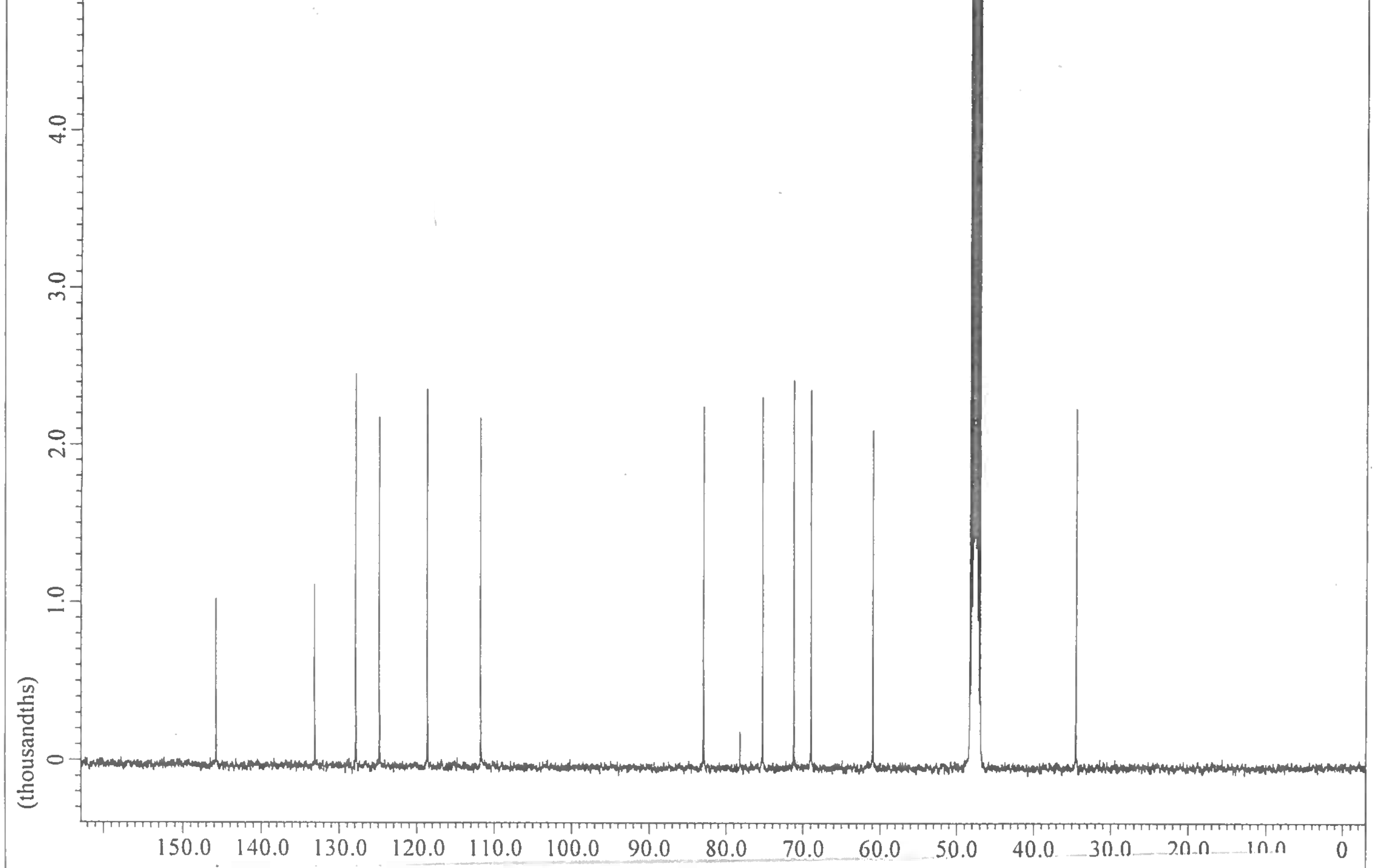

JEOL

Filename	= mdc-52_CARBON-3-
Author	= decastm
Experiment	= carbon.jxp
Sample_Id	= mdc-52
Solvent	= METHANOL-D4
Actual_Start_Time	= 9-JUL-2019 15:2
Revision_Time	= 10-JUL-2019 17:2
Data_Format	= 1D COMPLEX
Dim_Size	= 26214
X_Domain	= Carbon13
Dim_Title	= Carbon13
Dim_Units	= [ppm]
Dimensions	= X
Site	= Farmingdale Stat
Spectrometer	= JNM-ECZ400S/L1
Field_Strength	= 9.389766[T] (400
X_Acq_Duration	= 1.03809024[s]
X_Domain	= Carbon13
X_Freq	= 100.52530333[MHz
X_Offset	= 100[ppm]
X_Points	= 32768
X_Prescans	= 4
X_Resolution	= 0.96330739[Hz]
X_Sweep	= 31.56565657[kHz]
X_Sweep_Clipped	= 25.25252525[kHz]
Irr_Domain	= Proton
Irr_Freq	= 399.78219838[MHz
Irr_Offset	= 5[ppm]
Blanking	= 5[us]
Clipped	= FALSE
Scans	= 5000
Total_Scans	= 5000
Relaxation_Delay	= 2[s]
Recvr_Gain	= 52
Temp_Get	= 18[dC]
X_90_Width	= 11.73[us]
X_Acq_Time	= 1.03809024[s]
X_Angle	= 30[deg]
X_Atn	= 7.9[dB]
X_Pulse	= 3.91[us]
Irr_Atn_Dec	= 27[dB]
Irr_Atn_Dec_Calc	= 27[dB]
Irr_Atn_Dec_Default_Calc	= 27[dB]
Irr_Atn_Noe	= 27[dB]
Irr_Dec_Bandwidth_Hz	= 4.7826087[kHz]
Irr_Dec_Bandwidth_Ppm	= 11.96303566[ppm]
Irr_Dec_Freq	= 399.78219838[MHz
Irr_Dec_Merit_Factor	= 2.2
Irr_Decoupling	= TRUE
Irr_Noe	= TRUE
Irr_Noise	= WALTZ
Irr_Offset_Default	= 5[ppm]
Irr_Pwidth	= 0.115[ms]
Irr_Pwidth_Default	= 0.115[ms]
Irr_Pwidth_Default_Calc	= 0.115[ms]
Irr_Pwidth_Temp1	= 0.115[ms]
Irr_Wurst	= FALSE
Decimation_Rate	= 0
Experiment_Path	= c:\Program Files
Initial_Wait	= 1[s]
Noe_Time	= 2[s]

Michael De Castro 209**

Synapt_18959 34 (0.691)

SYNAPTG2-Si#UGA305
13:04:56
1: TOF MS ES+
2.92e5

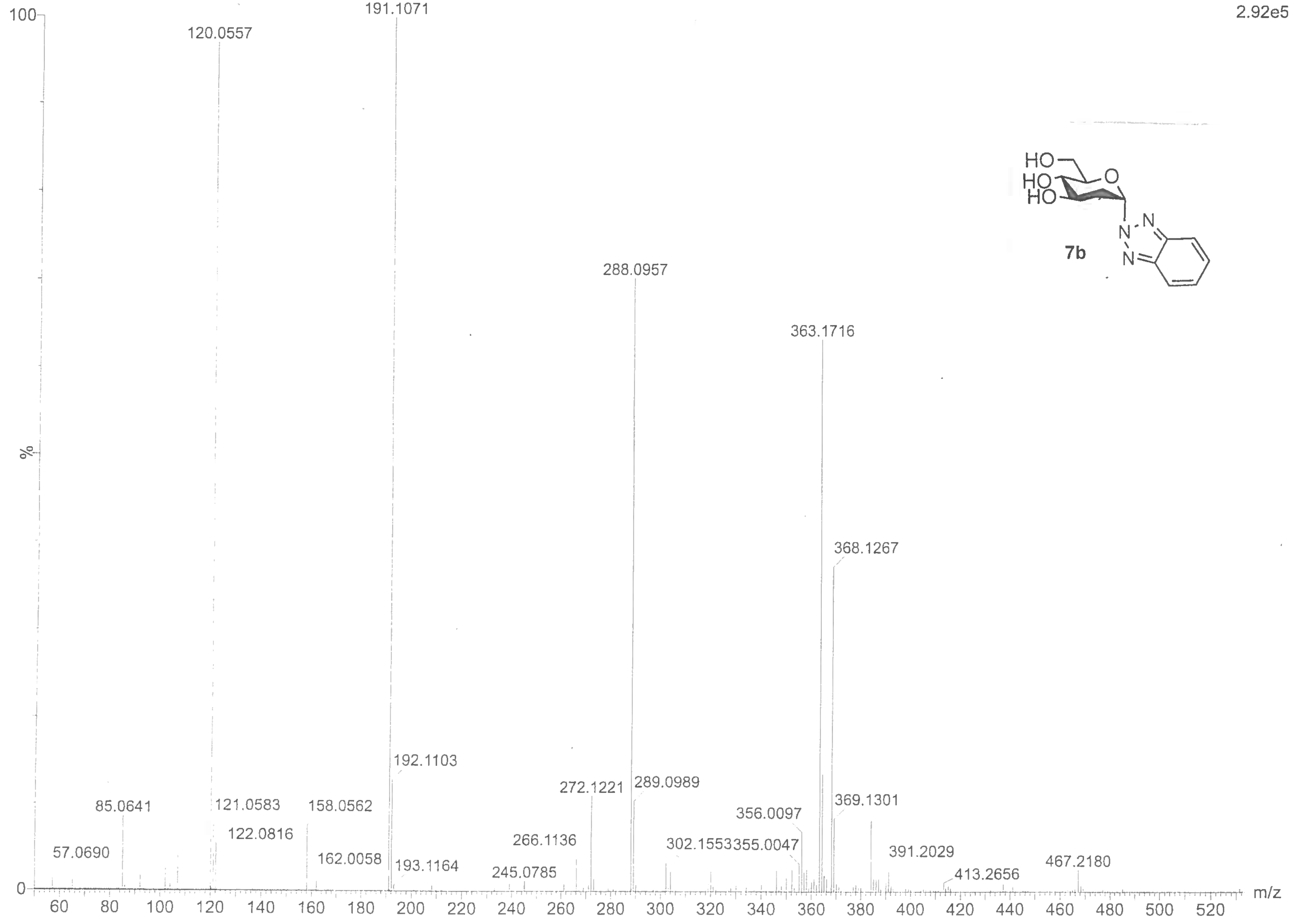

7b

Elemental Composition Report

Single Mass Analysis

Tolerance = 5.0 PPM / DBE: min = -50.0, max = 100.0
Element prediction: Off
Number of isotope peaks used for i-FIT = 9

Monoisotopic Mass, Even Electron Ions
182 formula(e) evaluated with 1 results within limits (up to 10 best isotopic matches for each mass)
Elements Used:
C: 0-90 H: 0-130 N: 2-4 O: 3-5 Na: 0-1

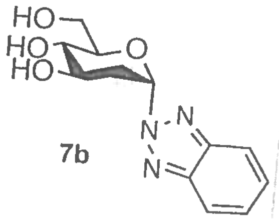

7b

Minimum:				-50.0				
Maximum:		5.0	5.0	100.0				
Mass	Calc. Mass	mDa	PPM	DBE	i-FIT	Norm	Conf(%)	Formula
288.0957	288.0960	-0.3	-1.0	6.5	159.2	n/a	n/a	C12 H15 N3 O4 Na

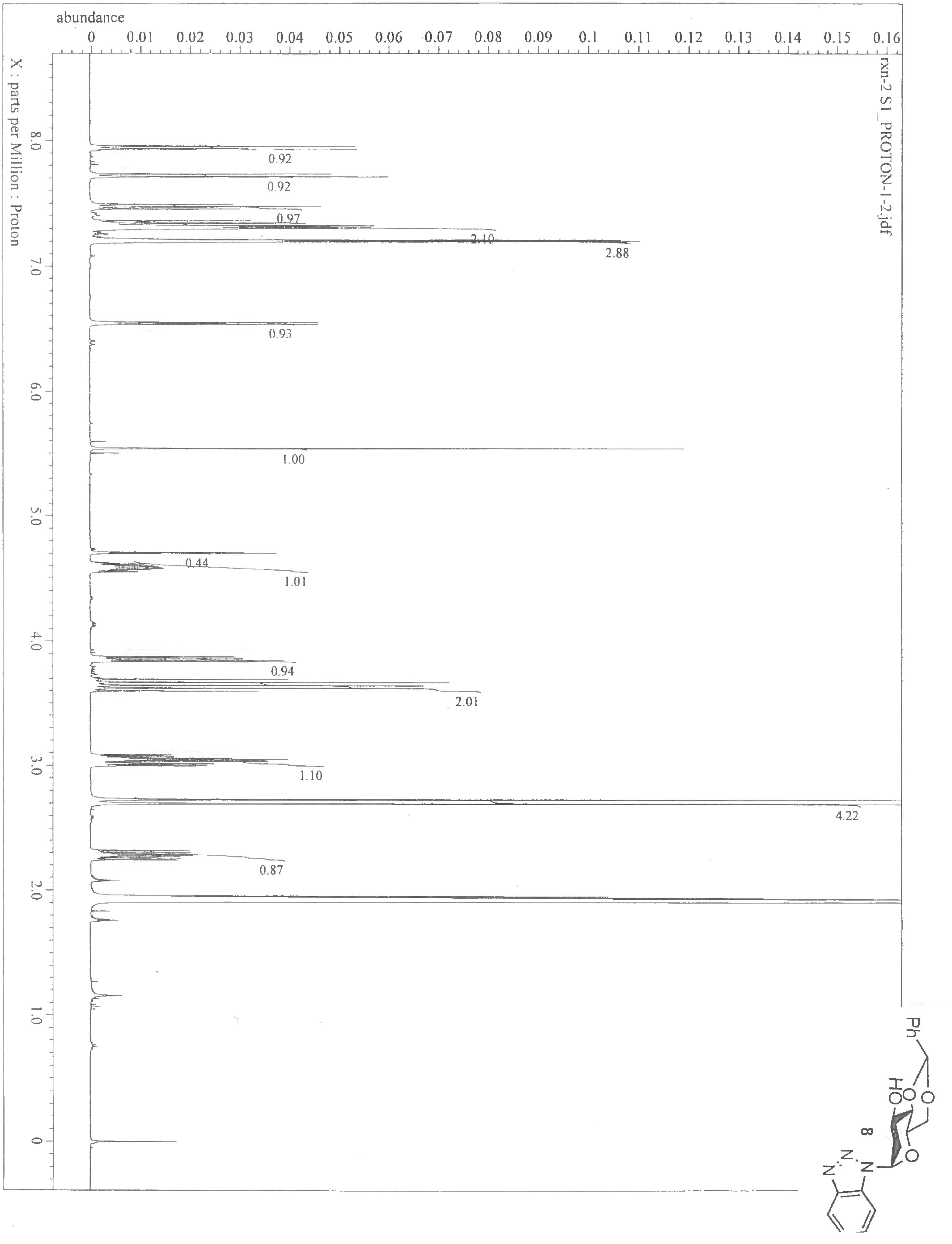

abundance
0 0.01 0.02 0.03 0.04 0.05 0.06 0.07 0.08 0.09 0.1 0.11 0.12 0.13 0.14 0.15 0.16
X : parts per Million : Proton
8.0 7.0 6.0 5.0 4.0 3.0 2.0 1.0 0
rxn-2 S1_PROTON-1-2.jdf
0.92
0.92
0.97
2.10
2.88
0.93
1.00
0.44
1.01
0.94
2.01
1.10
4.22
0.87
Ph
HO
O
N
8

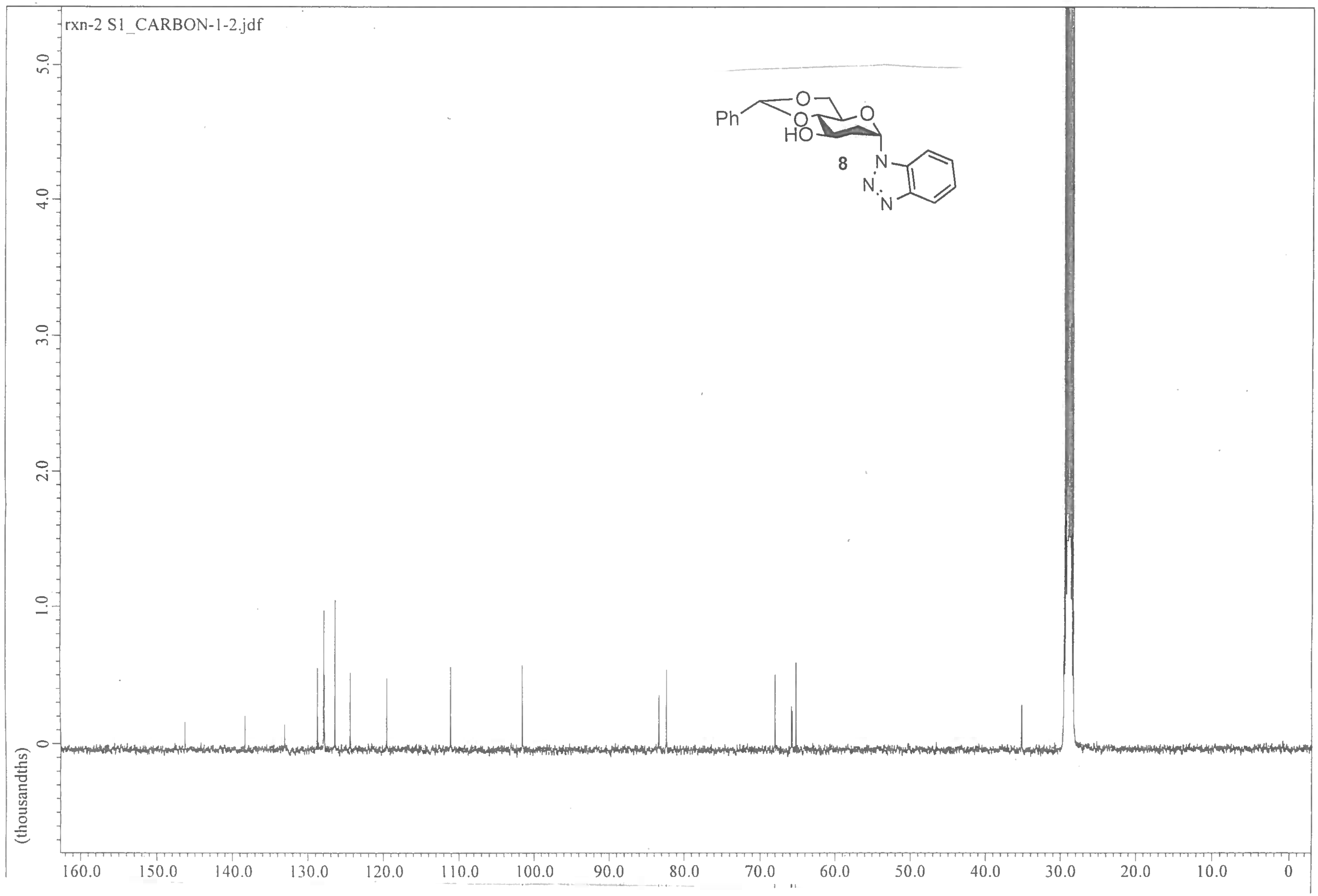
rxn-2 S1_CARBON-1-2.jdf
Ph
O
O
O
HO
N
N
N
8
(thousandths)
0
1.0
2.0
3.0
4.0
5.0
160.0
150.0
140.0
130.0
120.0
110.0
100.0
90.0
80.0
70.0
60.0
50.0
40.0
30.0
20.0
10.0
0

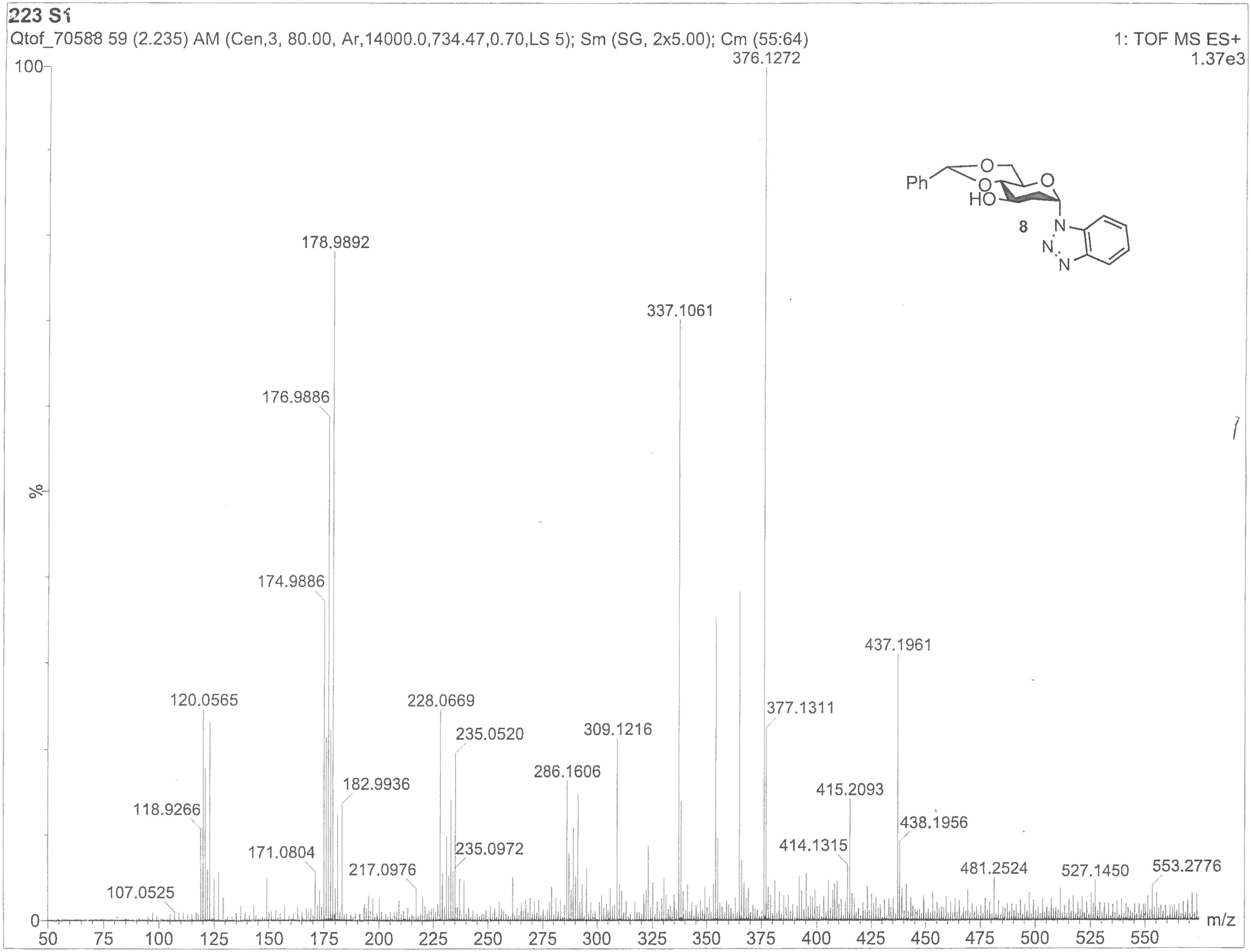
223 S1
Qtof_70588 59 (2.235) AM (Cen,3, 80.00, Ar,14000.0,734.47,0.70,LS 5); Sm (SG, 2x5.00); Cm (55:64)
1: TOF MS ES+
1.37e3
100
%
0
376.1272
178.9892
337.1061
176.9886
174.9886
437.1961
120.0565
228.0669
377.1311
235.0520
309.1216
286.1606
182.9936
415.2093
118.9266
438.1956
414.1315
235.0972
171.0804
217.0976
481.2524
553.2776
527.1450
107.0525
50
75
100
125
150
175
200
225
250
275
300
325
350
375
400
425
450
475
500
525
550
m/z
Ph
O
O
O
HO
N
N
N
8

223 S1

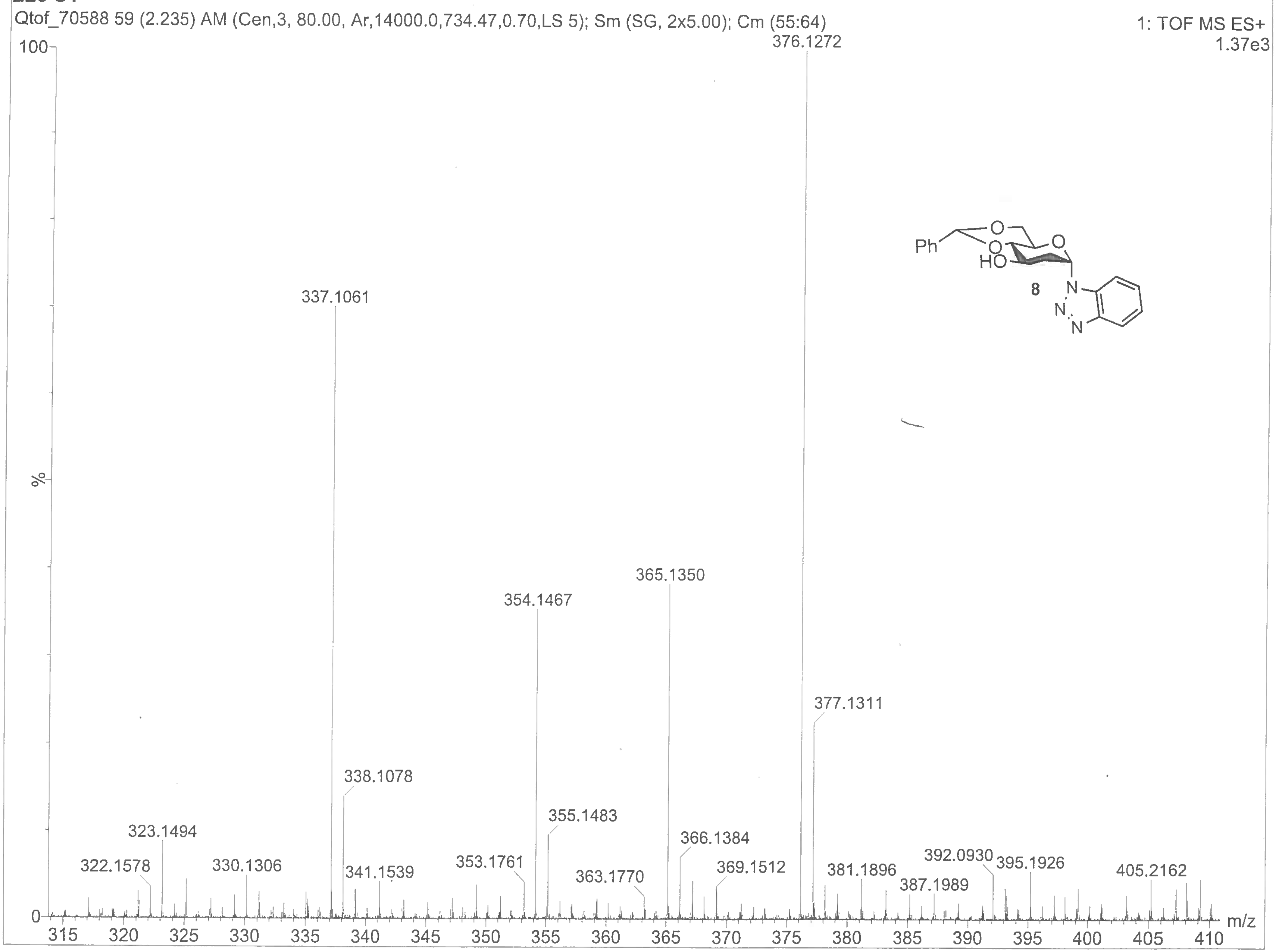

Qtof_70588 59 (2.235) AM (Cen,3, 80.00, Ar,14000.0,734.47,0.70,LS 5); Sm (SG, 2x5.00); Cm (55:64)
1: TOF MS ES+
1.37e3
100
%
0
376.1272
337.1061
365.1350
354.1467
377.1311
338.1078
355.1483
323.1494
366.1384
322.1578
330.1306
341.1539
353.1761
363.1770
369.1512
381.1896
387.1989
392.0930
395.1926
405.2162
315
320
325
330
335
340
345
350
355
360
365
370
375
380
385
390
395
400
405
410
m/z
Ph
O
O
O
HO
N
N
N
8

Single Mass Analysis
Tolerance = 5.0 PPM / DBE: min = -1.5, max = 100.0
Element prediction: Off
Number of isotope peaks used for i-FIT = 3

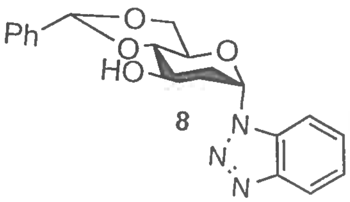

8

Monoisotopic Mass, Even Electron Ions
258 formula(e) evaluated with 1 results within limits (up to 50 closest results for each mass)
Elements Used:
C: 0-200 H: 0-200 N: 0-5 O: 4-9 Na: 0-1
223 S1
Qtof_70588 59 (2.235) AM (Cen,3, 80.00, Ar,14000.0,734.47,0.70,LS 5); Sm (SG, 2x5.00); Cm (55:64)

1: TOF MS ES+
4.84e+002

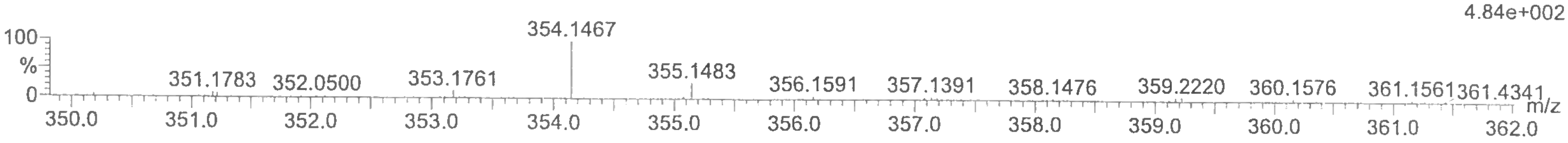

Mass	Calc. Mass	mDa	PPM	DBE	i-FIT	Formula
Minimum:				-1.5		
Maximum:		5.0	5.0	100.0		
354.1467	354.1454	1.3	3.7	11.5	3.8	C19 H20 N3 O4

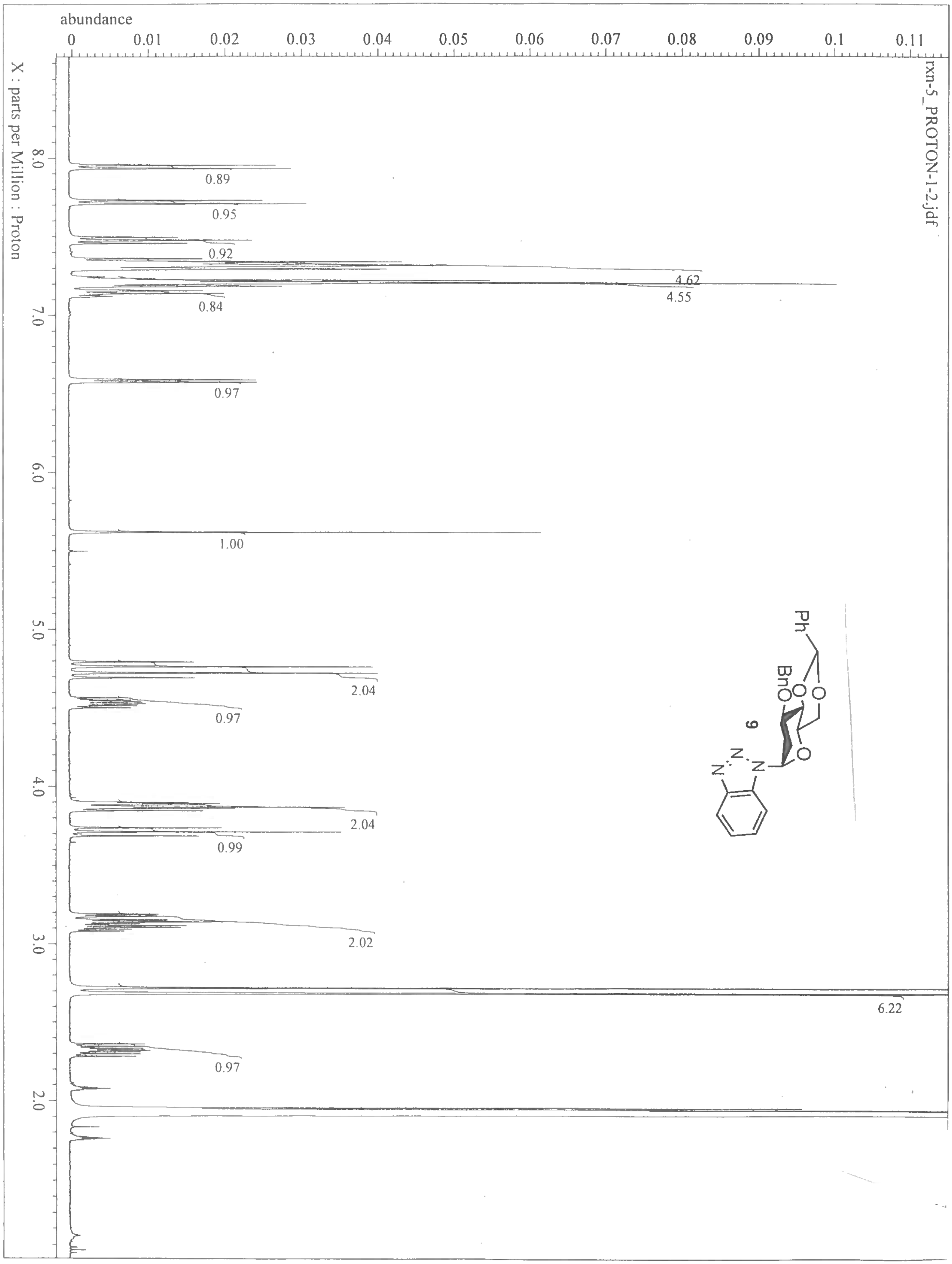
abundance
0 0.01 0.02 0.03 0.04 0.05 0.06 0.07 0.08 0.09 0.1 0.11
X : parts per Million : Proton
8.0 7.0 6.0 5.0 4.0 3.0 2.0
rxn-5_PROTON-1-2.jdf
0.89
0.95
0.92
4.62
4.55
0.84
0.97
1.00
2.04
0.97
2.04
0.99
2.02
6.22
0.97
Ph
BnO
O
N
9

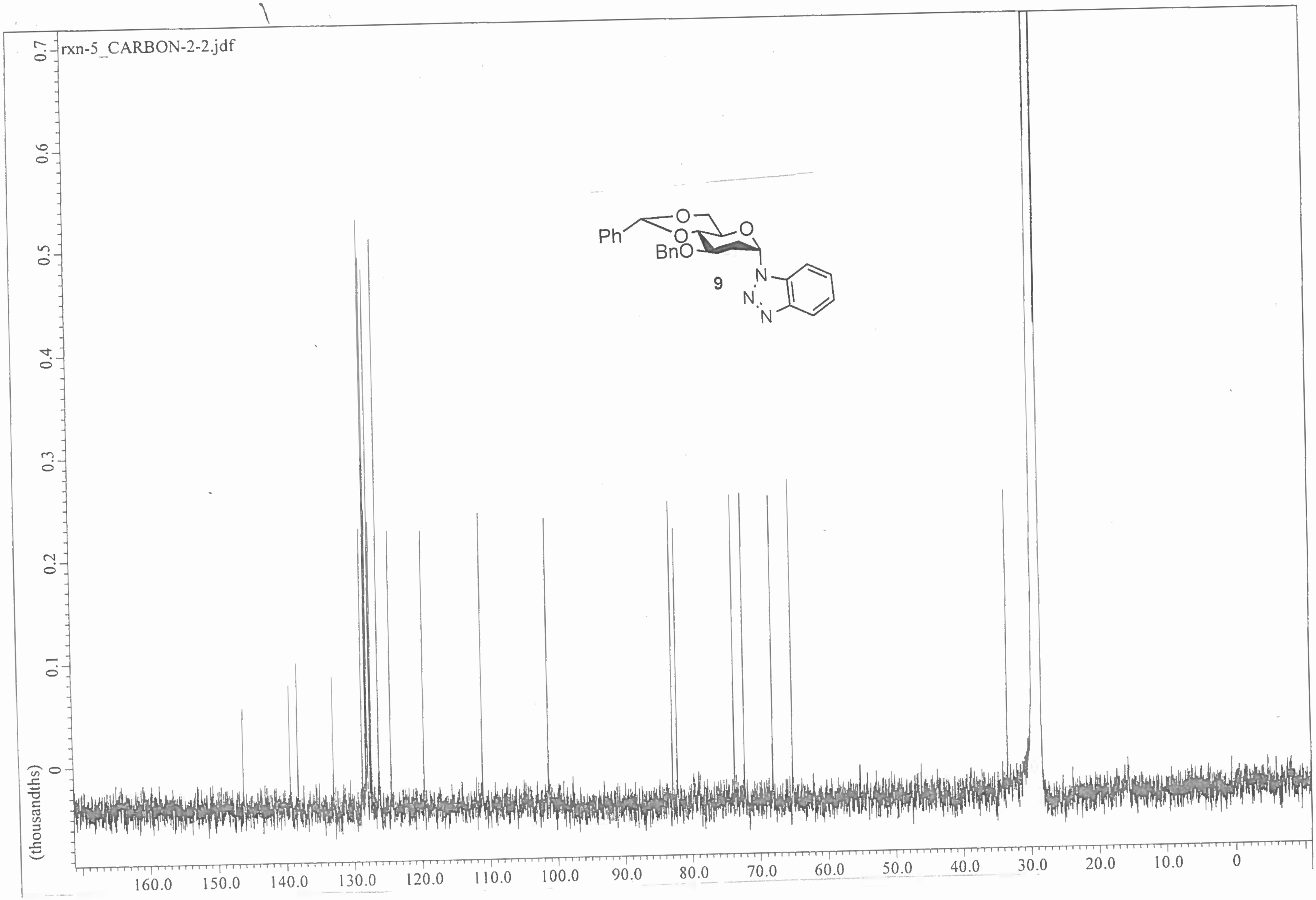

rxn-5_CARBON-2-2.jdf
Ph
BnO
O
N
9
(thousandths)
0
0.1
0.2
0.3
0.4
0.5
0.6
0.7
160.0
150.0
140.0
130.0
120.0
110.0
100.0
90.0
80.0
70.0
60.0
50.0
40.0
30.0
20.0
10.0

5HRMS

Qtof_70591 62 (2.336) AM (Cen,3, 80.00, Ar,14000.0,734.47,0.70,LS 5); Sm (SG, 2x5.00); Cm (62:67)

1: TOF MS ES+ 4.04e3

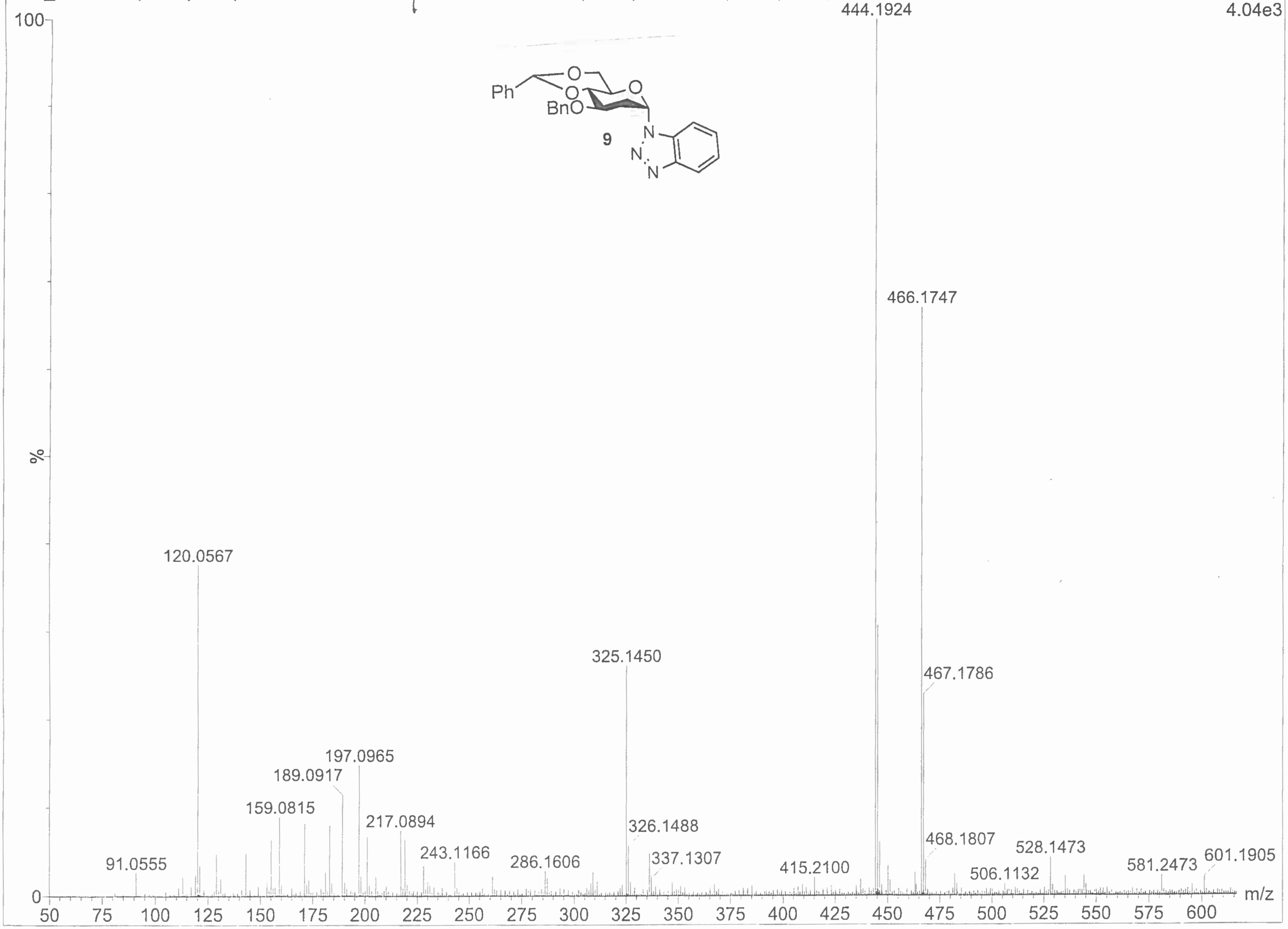

Elemental Composition Report

Single Mass Analysis

Tolerance = 5.0 PPM / DBE: min = -1.5, max = 100.0
Element prediction: Off
Number of isotope peaks used for i-FIT = 3

Monoisotopic Mass, Even Electron Ions
336 formula(e) evaluated with 1 results within limits (up to 50 closest results for each mass)
Elements Used:
C: 0-200 H: 0-200 N: 0-5 O: 4-9 Na: 0-1
5HRMS
Qtof_70591 62 (2.336) AM (Cen,3, 80.00, Ar,14000.0,734.47,0.70,LS 5); Sm (SG, 2x5.00); Cm (62:67)

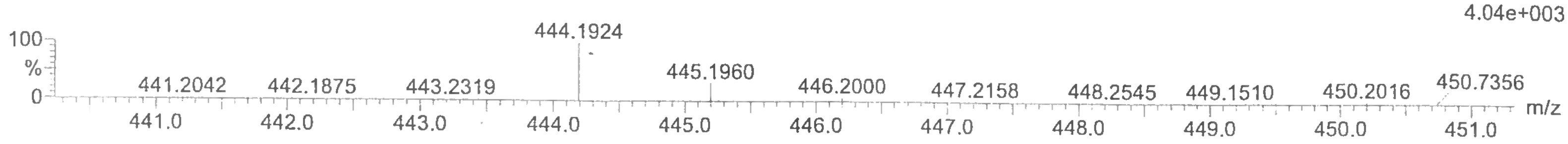

Mass	Calc. Mass	mDa	PPM	DBE	i-FIT	Formula
Minimum:				-1.5		
Maximum:		5.0	5.0	100.0		
444.1924	444.1923	0.1	0.2	15.5	1.7	C26 H26 N3 O4

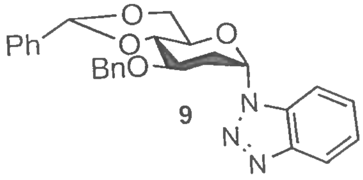

9

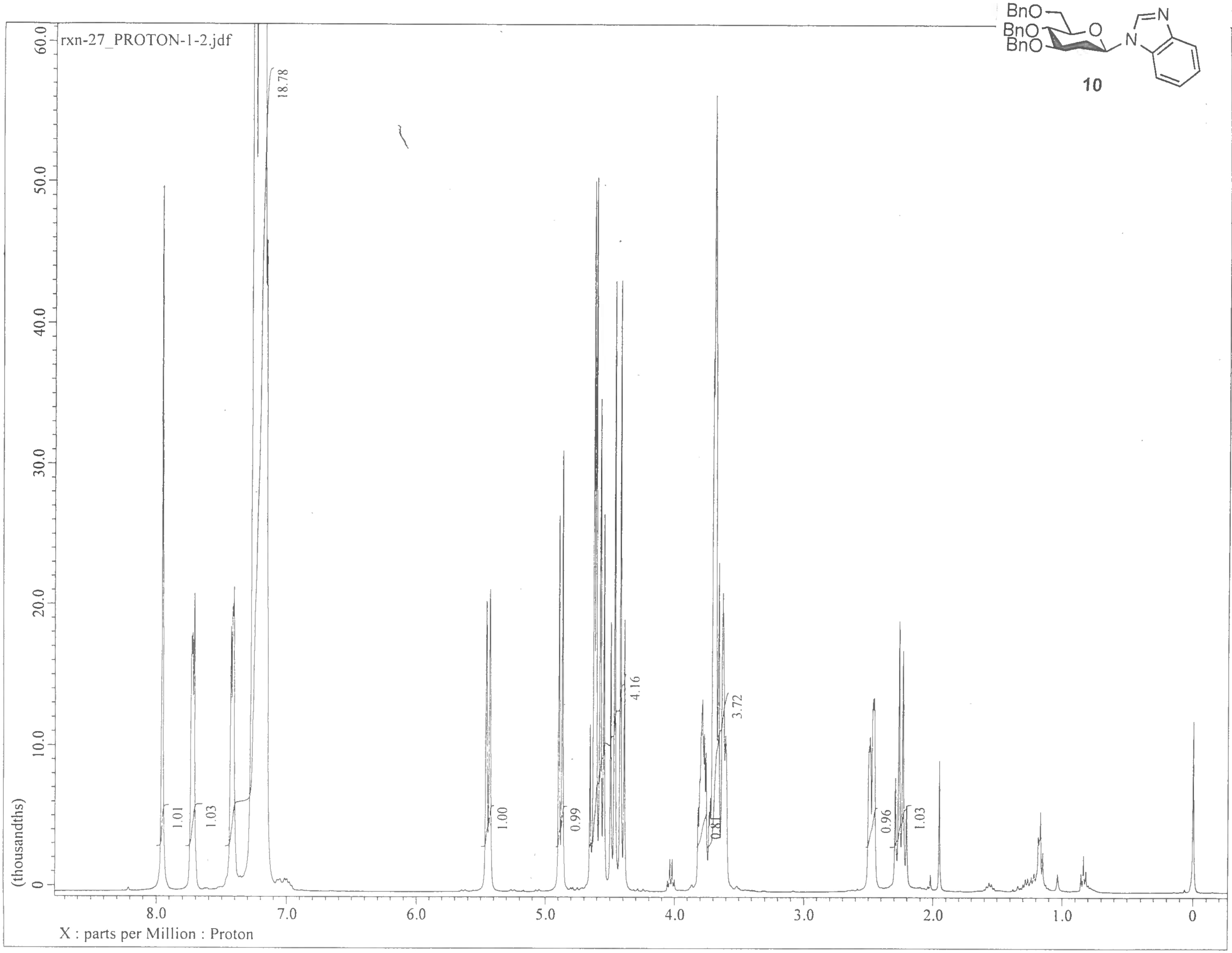
rxn-27_PROTON-1-2.jdf
(thousandths)
0
10.0
20.0
30.0
40.0
50.0
60.0
18.78
1.01
1.03
1.00
0.99
4.16
0.81
3.72
0.96
1.03
8.0
7.0
6.0
5.0
4.0
3.0
2.0
1.0
0
X : parts per Million : Proton
BnO
BnO
BnO
O
N
N

10

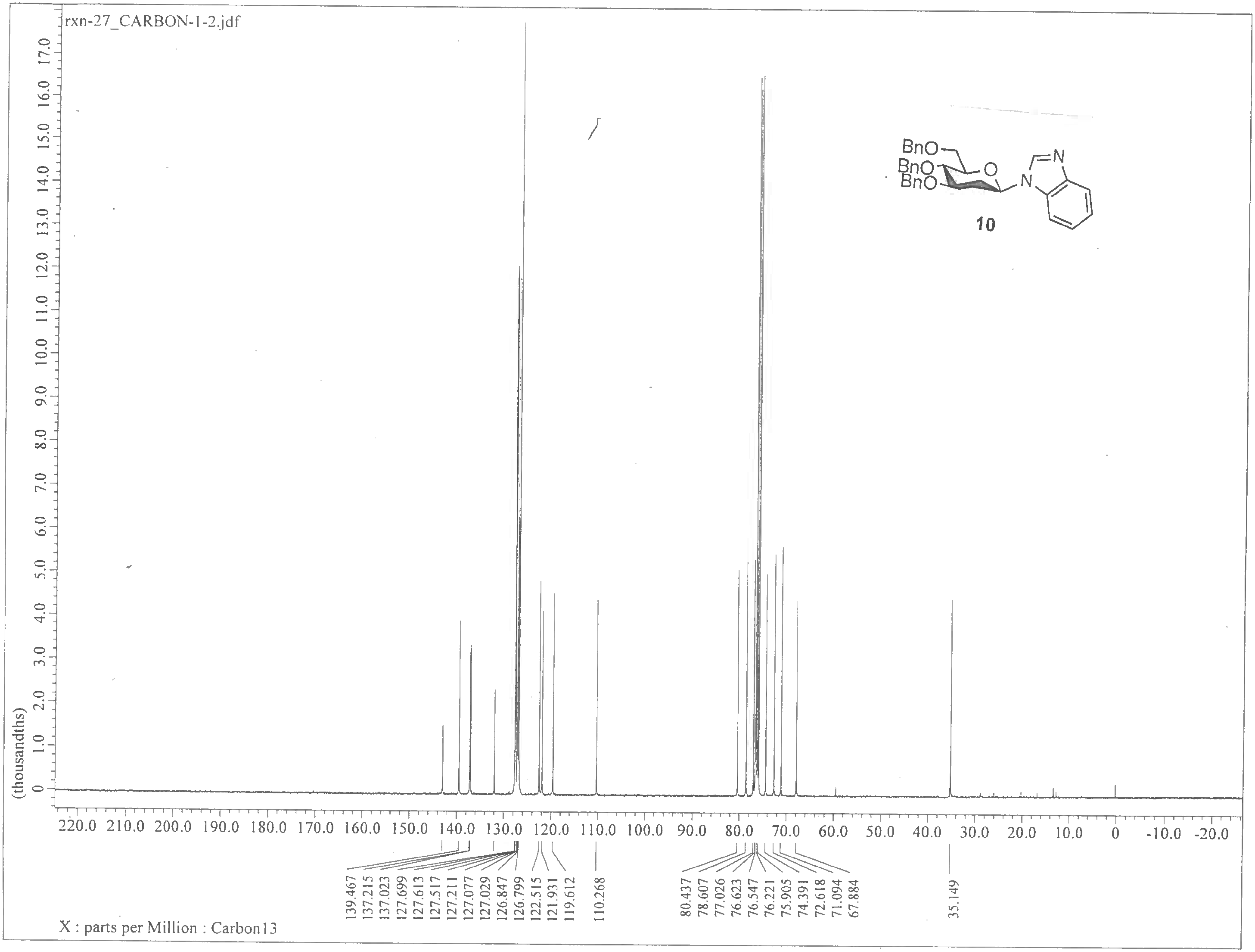
rxn-27_CARBON-1-2.jdf
(thousandths)
0
1.0
2.0
3.0
4.0
5.0
6.0
7.0
8.0
9.0
10.0
11.0
12.0
13.0
14.0
15.0
16.0
17.0
BnO
BnO
BnO
O
N
N
10
220.0
210.0
200.0
190.0
180.0
170.0
160.0
150.0
140.0
130.0
120.0
110.0
100.0
90.0
80.0
70.0
60.0
50.0
40.0
30.0
20.0
10.0
0
-10.0
-20.0
139.467
137.215
137.023
127.699
127.613
127.517
127.211
127.077
127.029
126.847
126.799
122.515
121.931
119.612
110.268
80.437
78.607
77.026
76.623
76.547
76.221
75.905
74.391
72.618
71.094
67.884
35.149
X : parts per Million : Carbon13

27HRMS

Qtof_70590 65 (2.471) AM (Cen,3, 80.00, Ar,14000.0,734.47,0.70,LS 5); Sm (SG, 2x5.00); Cm (61:65) 1: TOF MS ES+ 2.35e3

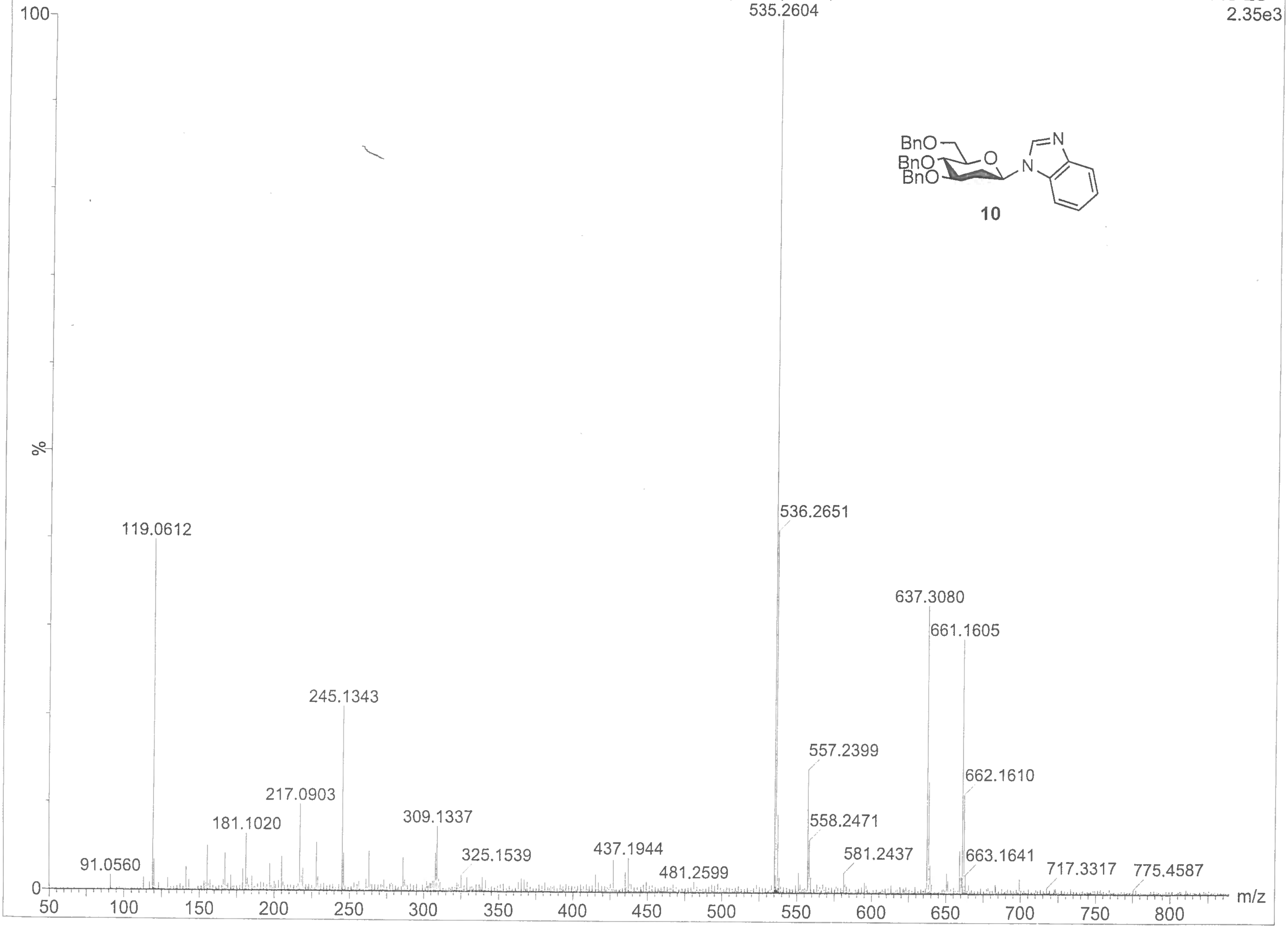

Elemental Composition Report

Single Mass Analysis

Tolerance = 5.0 PPM / DBE: min = -1.5, max = 100.0
Element prediction: Off
Number of isotope peaks used for i-FIT = 3

Monoisotopic Mass, Even Electron Ions
413 formula(e) evaluated with 1 results within limits (up to 50 closest results for each mass)
Elements Used:
C: 0-200 H: 0-200 N: 0-5 O: 4-9 Na: 0-1
27HRMS
Qtof_70590 65 (2.471) AM (Cen,3, 80.00, Ar,14000.0,734.47,0.70,LS 5); Sm (SG, 2x5.00); Cm (61:65)

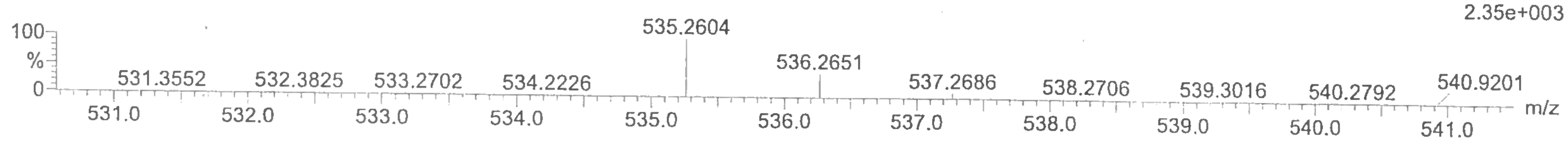

Minimum:				-1.5		
Maximum:		5.0	5.0	100.0		
Mass	Calc. Mass	mDa	PPM	DBE	i-FIT	Formula
535.2604	535.2597	0.7	1.3	18.5	1.2	C34 H35 N2 O4

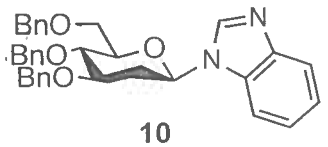

10

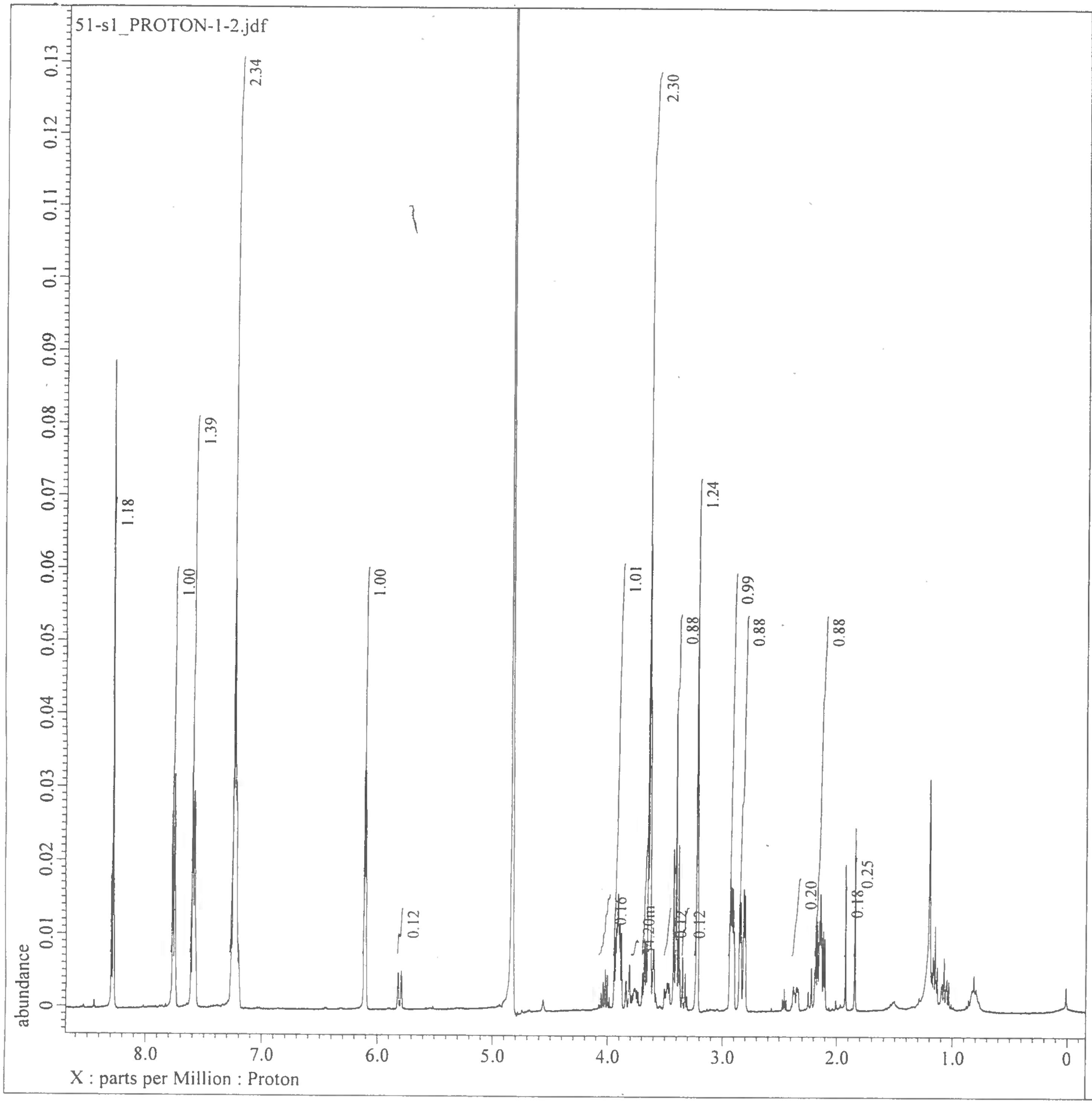

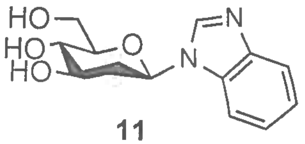

11

Filename	= 51-s1_PROTON-1-2.jd
Author	= decastm
Experiment	= proton.jxp
Sample_Id	= 51-s1
Solvent	= METHANOL-D4
Actual_Start_Time	= 15-JUL-2019 14:45:2
Revision_Time	= 15-JUL-2019 14:50:0
Data_Format	= 1D COMPLEX
Dim_Size	= 26214
X_Domain	= Proton
Dim_Title	= Proton
Dim_Units	= [ppm]
Dimensions	= X
Site	= Farmingdale State C
Spectrometer	= JNM-ECZ400S/L1
Field_Strength	= 9.389766[T] (400[MH
X_Acq_Duration	= 4.37256192[s]
X_Domain	= Proton
X_Freq	= 399.78219838[MHz]
X_Offset	= 5[ppm]
X_Points	= 32768
X_Prescans	= 0
X_Resolution	= 0.22869888[Hz]
X_Sweep	= 7.4940048[kHz]
X_Sweep_Clipped	= 5.99520384[kHz]
Irr_Domain	= Proton
Irr_Freq	= 399.78219838[MHz]
Irr_Offset	= 5[ppm]
Tri_Domain	= Proton
Tri_Freq	= 399.78219838[MHz]
Tri_Offset	= 5[ppm]
Blanking	= 2[us]
Clipped	= FALSE
Scans	= 16
Total_Scans	= 16
Relaxation_Delay	= 4[s]
Recvr_Gain	= 52
Temp_Get	= 17.4[dC]
X_90_Width	= 5.85[us]
X_Acq_Time	= 4.37256192[s]
X_Angle	= 45[deg]
X_Atn	= 1.3[dB]
X_Pulse	= 2.925[us]
Irr_Mode	= Off
Tri_Mode	= Off
Dante_Loop	= 400
Dante_Presat	= FALSE
Decimation_Rate	= 0
Experiment_Path	= c:\Program Files\JE
Initial_Wait	= 1[s]
Phase	= {0, 90, 270, 180, 1
Presat_Time	= 4[s]
Presat_Time_Flag	= FALSE
Relaxation_Delay_Calc	= 0[s]
Relaxation_Delay_Temp	= 4[s]
Repetition_Time	= 8.37256192[s]

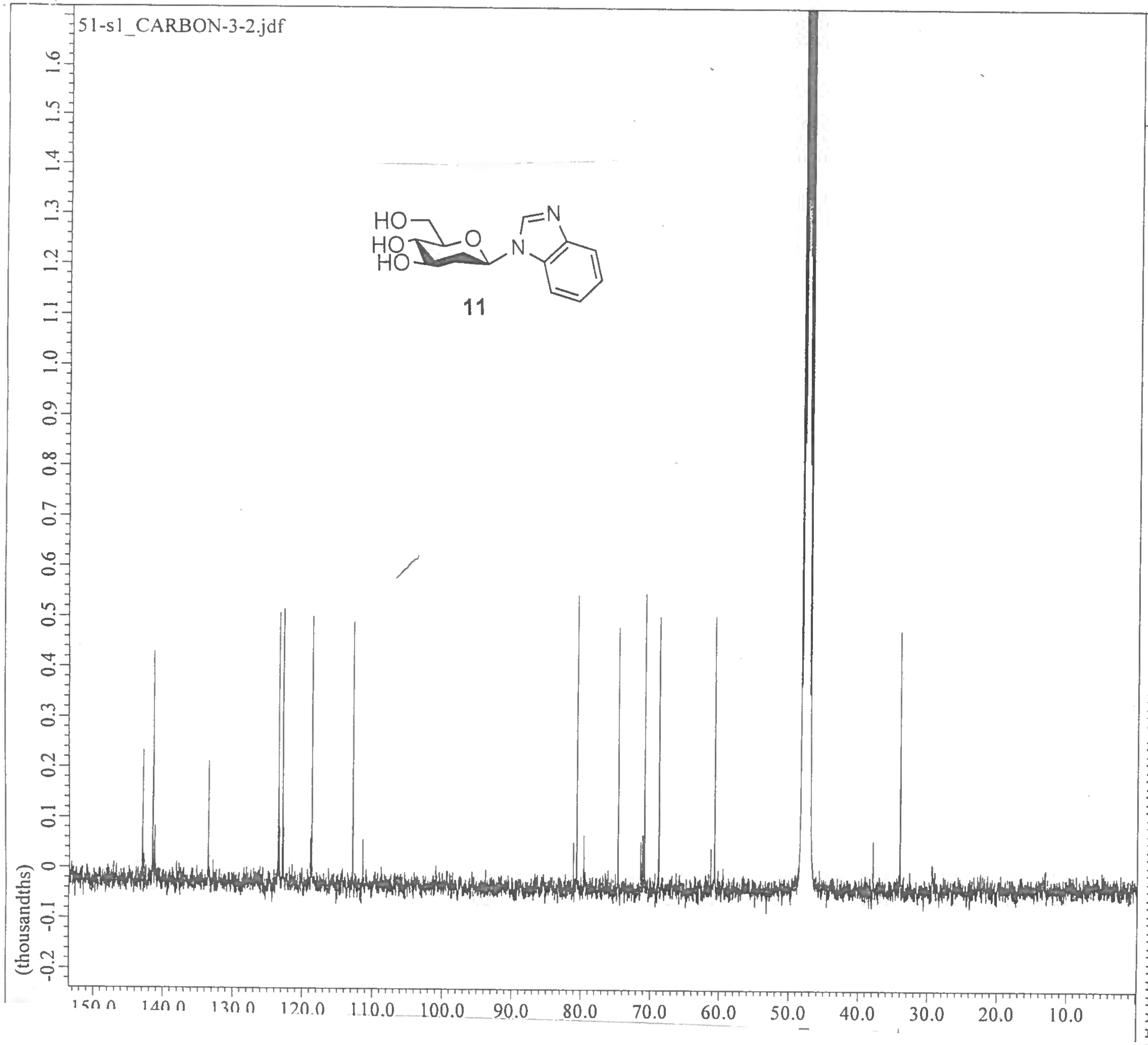

JEOL

Filename	= 51-s1_CARBON-3-2
Author	= decastm
Experiment	= carbon.jxp
Sample_Id	= 51-s1
Solvent	= METHANOL-D4
Actual_Start_Time	= 15-JUL-2019 18:2
Revision_Time	= 16-JUL-2019 15:0
Data_Format	= 1D COMPLEX
Dim_Size	= 26214
X_Domain	= Carbon13
Dim_Title	= Carbon13
Dim_Units	= [ppm]
Dimensions	= X
Site	= Farmingdale Stat
Spectrometer	= JNM-ECZ400S/L1
Field_Strength	= 9.389766[T] (400
X_Acq_Duration	= 1.03809024[s]
X_Domain	= Carbon13
X_Freq	= 100.52530333[MHz
X_Offset	= 100[ppm]
X_Points	= 32768
X_Prescans	= 4
X_Resolution	= 0.96330739[Hz]
X_Sweep	= 31.56565657[kHz]
X_Sweep_Clipped	= 25.25252525[kHz]
Irr_Domain	= Proton
Irr_Freq	= 399.78219838[MHz
Irr_Offset	= 5[ppm]
Blanking	= 5[us]
Clipped	= FALSE
Scans	= 10000
Total_Scans	= 10000
Relaxation_Delay	= 2[s]
Recvr_Gain	= 52
Temp_Get	= 18.8[dC]
X_90_Width	= 11.73[us]
X_Acq_Time	= 1.03809024[s]
X_Angle	= 30[deg]
X_Atn	= 7.9[dB]
X_Pulse	= 3.91[us]
Irr_Atn_Dec	= 27[dB]
Irr_Atn_Dec_Calc	= 27[dB]
Irr_Atn_Dec_Default_Calc	= 27[dB]
Irr_Atn_Noe	= 27[dB]
Irr_Dec_Bandwidth_Hz	= 4.7826087[kHz]
Irr_Dec_Bandwidth_Ppm	= 11.96303566[ppm]
Irr_Dec_Freq	= 399.78219838[MHz
Irr_Dec_Merit_Factor	= 2.2
Irr_Decoupling	= TRUE
Irr_Noe	= TRUE
Irr_Noise	= WALTZ
Irr_Offset_Default	= 5[ppm]
Irr_Pwidth	= 0.115[ms]
Irr_Pwidth_Default	= 0.115[ms]
Irr_Pwidth_Default_Calc	= 0.115[ms]
Irr_Pwidth_Templ	= 0.115[ms]
Irr_Wurst	= FALSE
Decimation_Rate	= 0
Experiment_Path	= c:\Program Files
Initial_Wait	= 1[s]
Noe_Time	= 2[s]

Michael De Castro   mdc-51-N

SYNAPTG2-Si#NotSet
12:17:54

Synapt_22442 26 (0.536)

1: TOF MS ES+
1.28e5

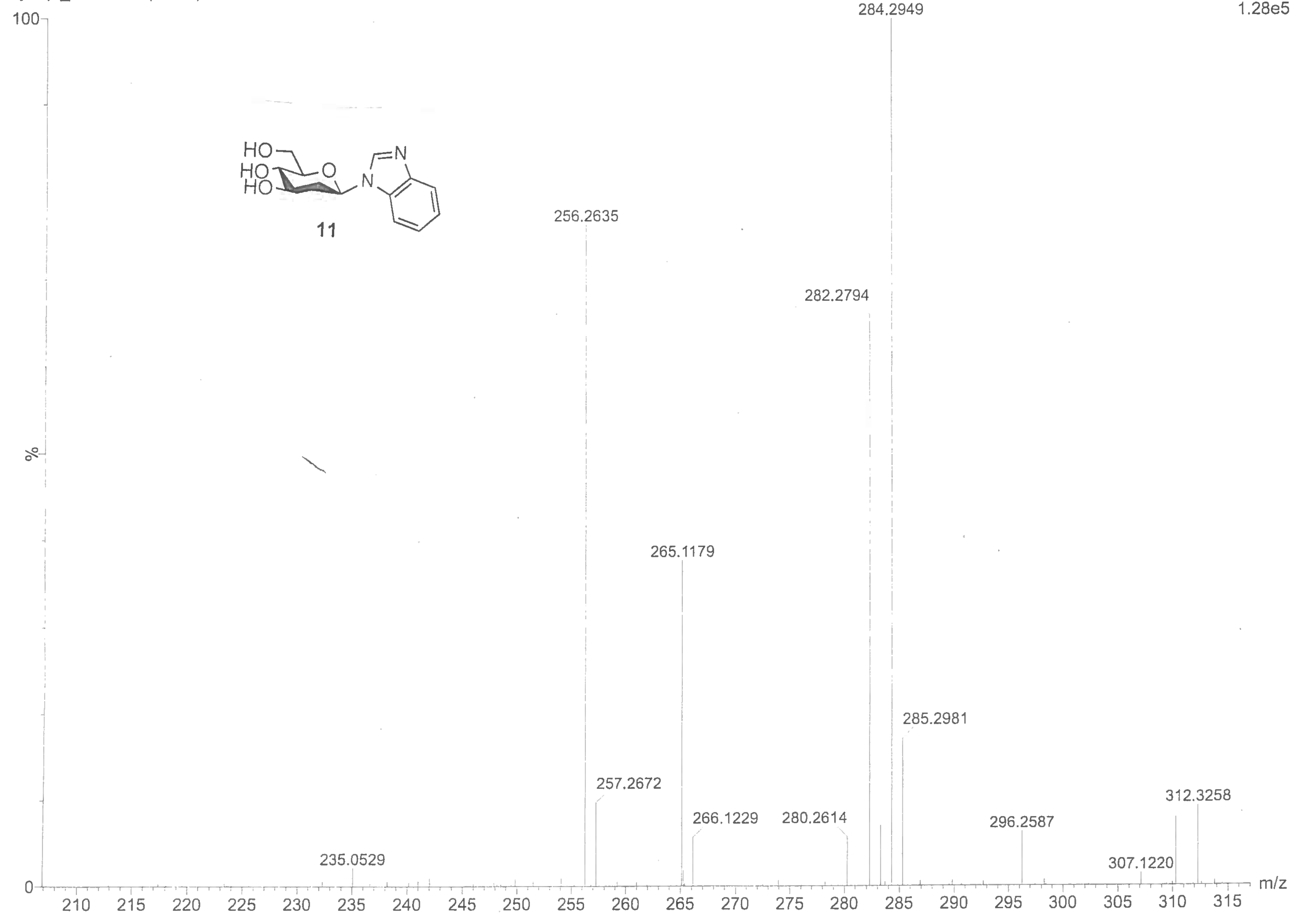

Michael De Castro mdc-51-N

SYNAPTG2-Si#NotSet
12:17:54
1: TOF MS ES+
2.22e5

Synapt_22442 26 (0.536)

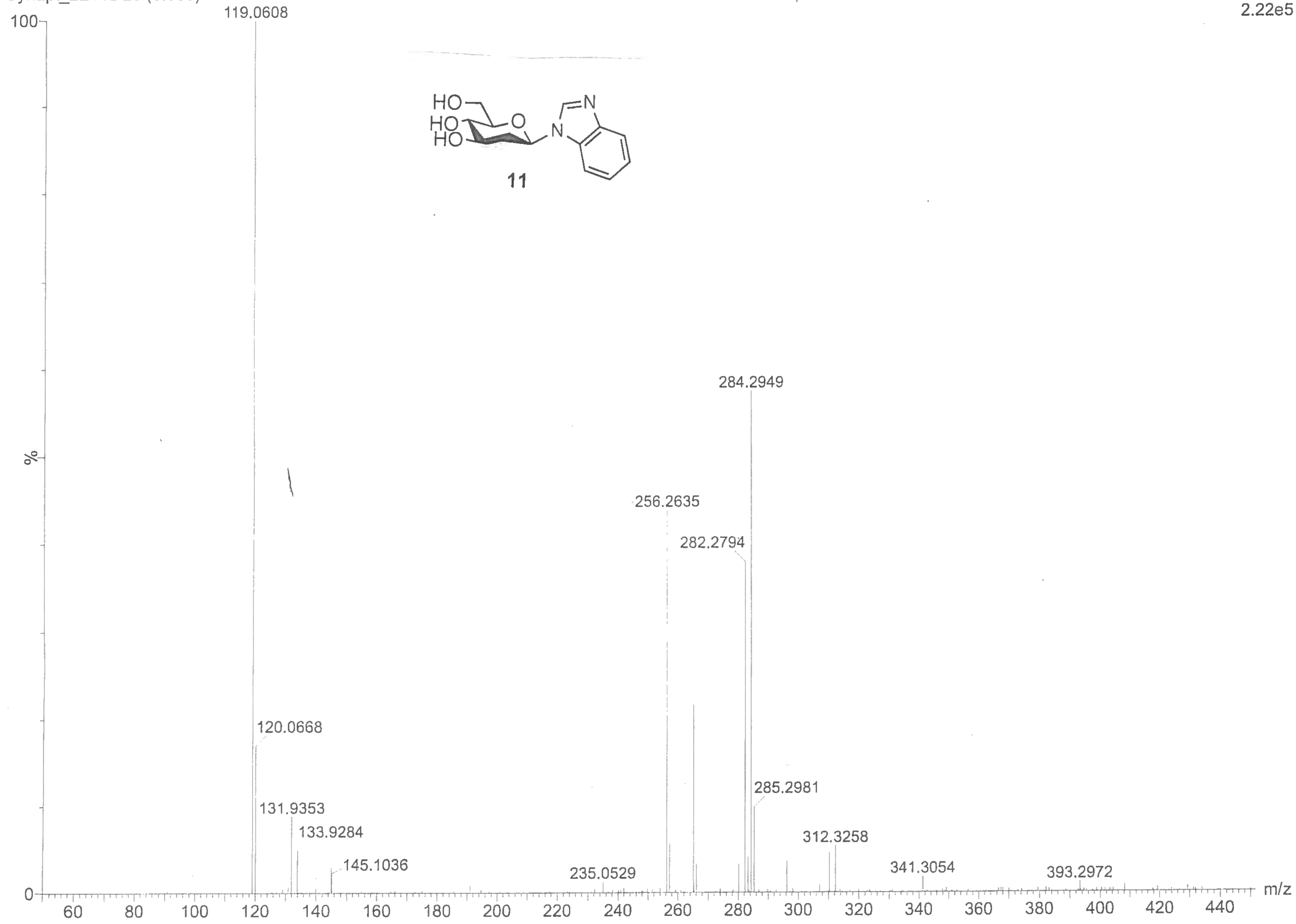

Elemental Composition Report

Single Mass Analysis

Tolerance = 5.0 PPM / DBE: min = -50.0, max = 100.0
Element prediction: Off
Number of isotope peaks used for i-FIT = 9

Monoisotopic Mass, Even Electron Ions
7 formula(e) evaluated with 1 results within limits (up to 10 best isotopic matches for each mass)
Elements Used:
C: 0-50 H: 0-80 N: 2-2 O: 4-4

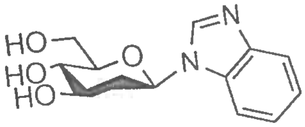

11

Minimum:				-50.0				
Maximum:		5.0	5.0	100.0				
Mass	Calc. Mass	mDa	PPM	DBE	i-FIT	Norm	Conf(%)	Formula
265.1179	265.1188	-0.9	-3.4	6.5	104.3	n/a	n/a	C13 H17 N2 O4

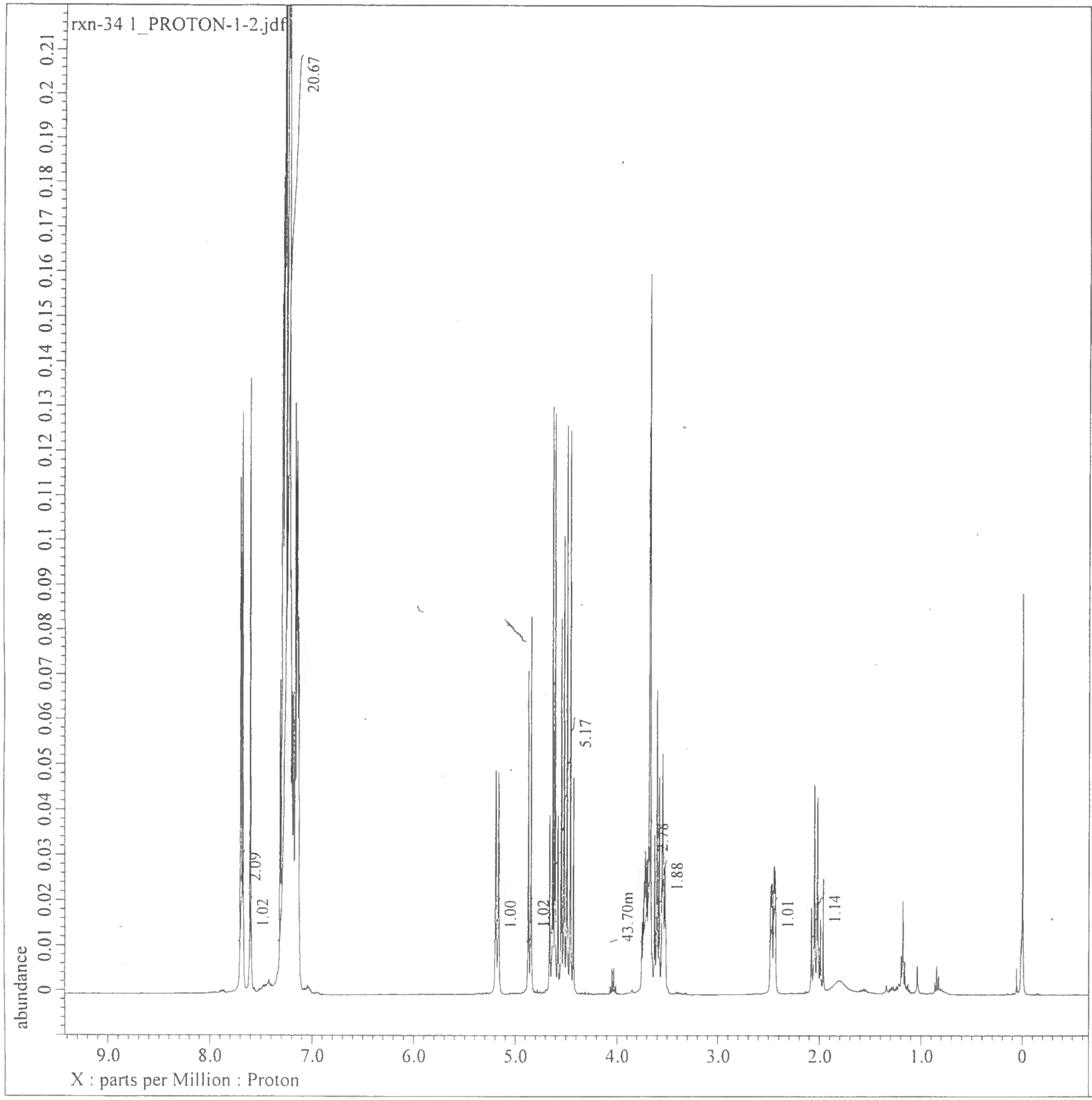

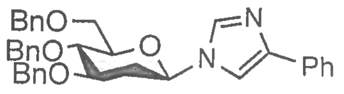

13

Filename	= rxn-34 1_PROTON-1-2
Author	= decastm
Experiment	= proton.jxp
Sample_Id	= rxn-34 1
Solvent	= CHLOROFORM-D
Actual_Start_Time	= 14-MAY-2019 13:23:0
Revision_Time	= 14-MAY-2019 13:27:4
Data_Format	= 1D COMPLEX
Dim_Size	= 26214
X_Domain	= Proton
Dim_Title	= Proton
Dim_Units	= [ppm]
Dimensions	= X
Site	= Farmingdale State C
Spectrometer	= JNM-ECZ400S/L1
Field_Strength	= 9.389766[T] (400[MH
X_Acq_Duration	= 4.37256192[s]
X_Domain	= Proton
X_Freq	= 399.78219838[MHz]
X_Offset	= 5[ppm]
X_Points	= 32768
X_Prescans	= 0
X_Resolution	= 0.22869888[Hz]
X_Sweep	= 7.4940048[kHz]
X_Sweep_Clipped	= 5.99520384[kHz]
Irr_Domain	= Proton
Irr_Freq	= 399.78219838[MHz]
Irr_Offset	= 5[ppm]
Tri_Domain	= Proton
Tri_Freq	= 399.78219838[MHz]
Tri_Offset	= 5[ppm]
Blanking	= 2[us]
Clipped	= FALSE
Scans	= 16
Total_Scans	= 16
Relaxation_Delay	= 4[s]
Recvr_Gain	= 42
Temp_Get	= 19.7[dC]
X_90_Width	= 5.85[us]
X_Acq_Time	= 4.37256192[s]
X_Angle	= 45[deg]
X_Atn	= 1.3[dB]
X_Pulse	= 2.925[us]
Irr_Mode	= Off
Tri_Mode	= Off
Dante_Loop	= 400
Dante_Presat	= FALSE
Decimation_Rate	= 0
Experiment_Path	= c:\Program Files\JE
Initial_Wait	= 1[s]
Phase	= {0, 90, 270, 180, 1
Presat_Time	= 4[s]
Presat_Time_Flag	= FALSE
Relaxation_Delay_Calc	= 0[s]
Relaxation_Delay_Temp	= 4[s]
Repetition_Time	= 8.37256192[s]

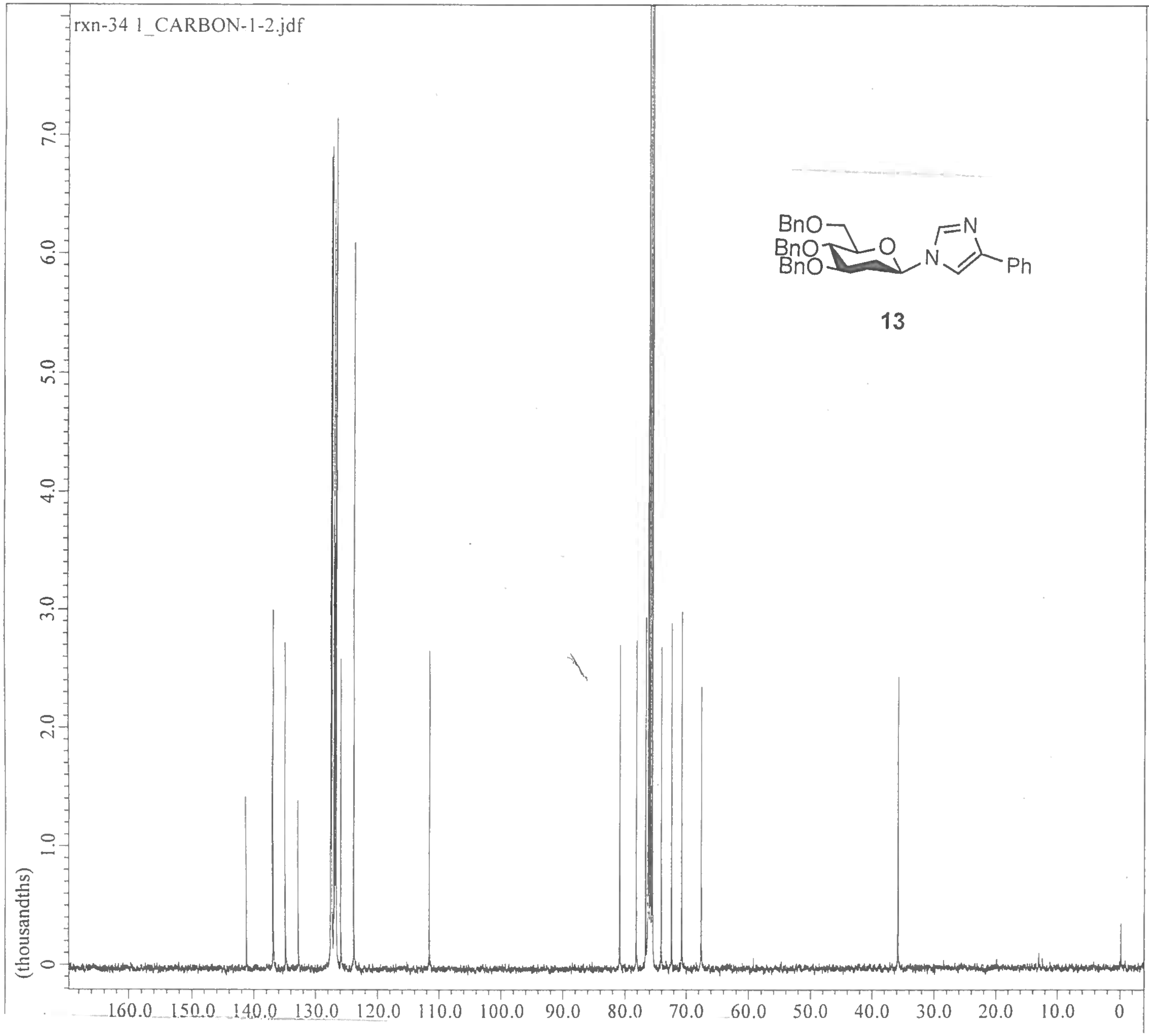

JEOL

Filename	= rxn-34 1_CARBON-
Author	= decastm
Experiment	= carbon.jxp
Sample_Id	= rxn-34 1
Solvent	= CHLOROFORM-D
Actual_Start_Time	= 14-MAY-2019 13:3
Revision_Time	= 14-MAY-2019 17:2
Data_Format	= 1D COMPLEX
Dim_Size	= 26214
X_Domain	= Carbon13
Dim_Title	= Carbon13
Dim_Units	= [ppm]
Dimensions	= X
Site	= Farmingdale Stat
Spectrometer	= JNM-ECZ400S/L1
Field_Strength	= 9.389766[T] (400
X_Acq_Duration	= 1.03809024[s]
X_Domain	= Carbon13
X_Freq	= 100.52530333[MHz
X_Offset	= 100[ppm]
X_Points	= 32768
X_Prescans	= 4
X_Resolution	= 0.96330739[Hz]
X_Sweep	= 31.56565657[kHz]
X_Sweep_Clipped	= 25.25252525[kHz]
Irr_Domain	= Proton
Irr_Freq	= 399.78219838[MHz
Irr_Offset	= 5[ppm]
Blanking	= 5[us]
Clipped	= FALSE
Scans	= 4511
Total_Scans	= 4511
Relaxation_Delay	= 2[s]
Recvr_Gain	= 52
Temp_Get	= 19.5[dC]
X_90_Width	= 11.73[us]
X_Acq_Time	= 1.03809024[s]
X_Angle	= 30[deg]
X_Atn	= 7.9[dB]
X_Pulse	= 3.91[us]
Irr_Atn_Dec	= 27[dB]
Irr_Atn_Dec_Calc	= 27[dB]
Irr_Atn_Dec_Default_Calc	= 27[dB]
Irr_Atn_Noe	= 27[dB]
Irr_Dec_Bandwidth_Hz	= 4.7826087[kHz]
Irr_Dec_Bandwidth_Ppm	= 11.96303566[ppm]
Irr_Dec_Freq	= 399.78219838[MHz
Irr_Dec_Merit_Factor	= 2.2
Irr_Decoupling	= TRUE
Irr_Noe	= TRUE
Irr_Noise	= WALTZ
Irr_Offset_Default	= 5[ppm]
Irr_Pwidth	= 0.115[ms]
Irr_Pwidth_Default	= 0.115[ms]
:h_Default_Calc	= 0.115[ms]
:h_Templ	= 0.115[ms]
:	= FALSE
on_Rate	= 0
it_Path	= c:\Program Files
Vait	= 1[s]
	= 2[s]

Michael De Castro mdc-34-N

Synapt_22437 36 (0.725)

SYNAPTG2-Si#NotSet
12:07:57
1: TOF MS ES+
3.60e6

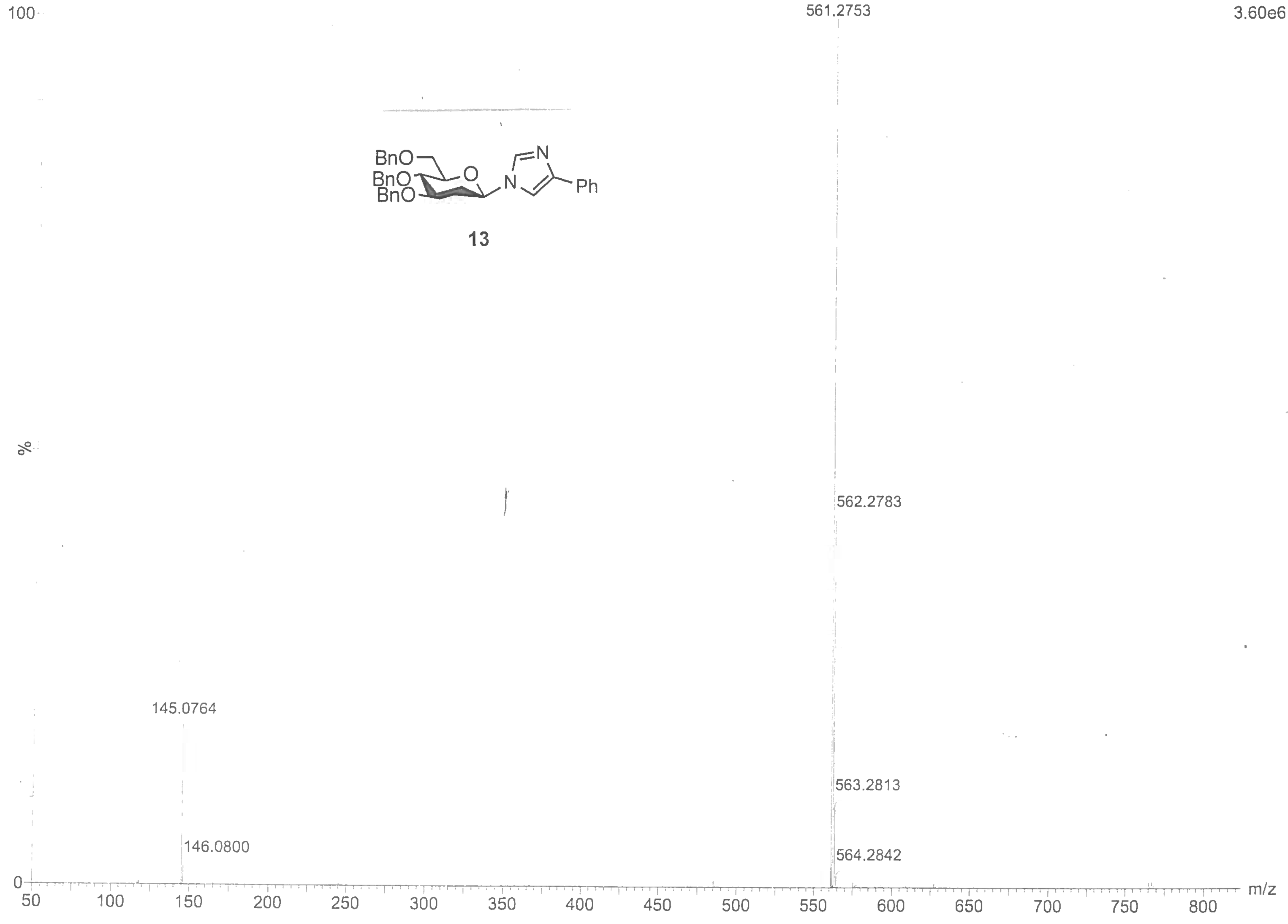

Elemental Composition Report

Single Mass Analysis

Tolerance = 5.0 PPM / DBE: min = -50.0, max = 100.0
Element prediction: Off
Number of isotope peaks used for i-FIT = 9

Monoisotopic Mass, Even Electron Ions
7 formula(e) evaluated with 1 results within limits (up to 10 best isotopic matches for each mass)
Elements Used:
C: 0-50 H: 0-80 N: 2-2 O: 4-4

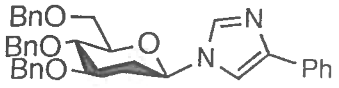

13

Minimum:				-50.0				
Maximum:		5.0	5.0	100.0				
Mass	Calc. Mass	mDa	PPM	DBE	i-FIT	Norm	Conf(%)	Formula
561.2753	561.2753	0.0	0.0	19.5	922.5	n/a	n/a	C36 H37 N2 O4

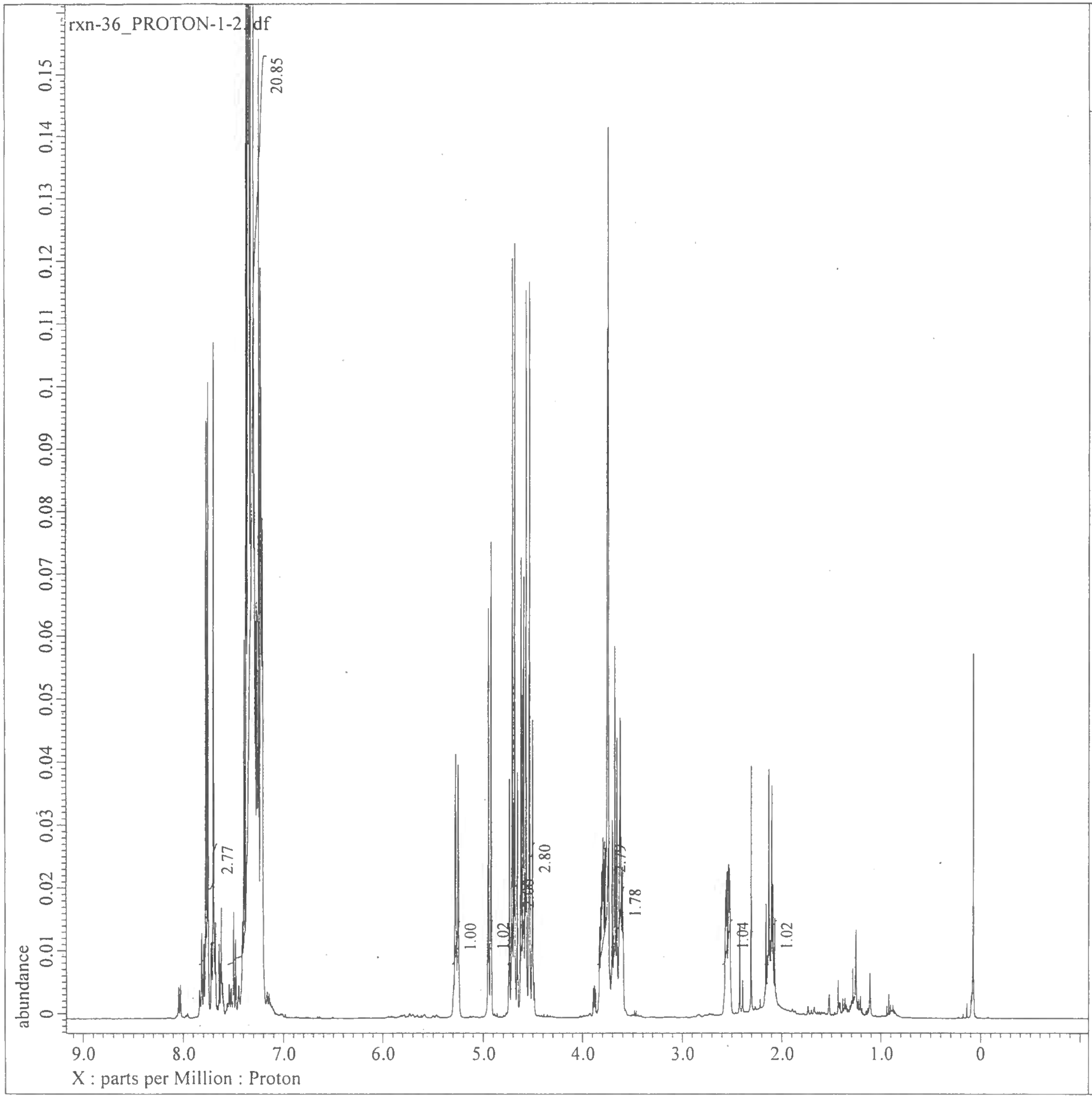

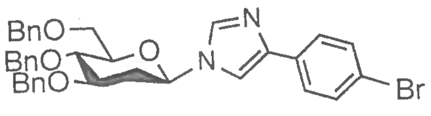

14

Filename	= rxn-36_PROTON-1-2.j
Author	= decastm
Experiment	= proton.jxp
Sample_Id	= rxn-36
Solvent	= CHLOROFORM-D
Actual_Start_Time	= 20-MAY-2019 12:19:1
Revision_Time	= 20-MAY-2019 12:25:1
Data_Format	= 1D COMPLEX
Dim_Size	= 26214
X_Domain	= Proton
Dim_Title	= Proton
Dim_Units	= [ppm]
Dimensions	= X
Site	= Farmingdale State C
Spectrometer	= JNM-ECZ400S/L1
Field_Strength	= 9.389766[T] (400[MH
X_Acq_Duration	= 4.37256192[s]
X_Domain	= Proton
X_Freq	= 399.78219838[MHz]
X_Offset	= 5[ppm]
X_Points	= 32768
X_Prescans	= 0
X_Resolution	= 0.22869888[Hz]
X_Sweep	= 7.4940048[kHz]
X_Sweep_Clipped	= 5.99520384[kHz]
Irr_Domain	= Proton
Irr_Freq	= 399.78219838[MHz]
Irr_Offset	= 5[ppm]
Tri_Domain	= Proton
Tri_Freq	= 399.78219838[MHz]
Tri_Offset	= 5[ppm]
Blanking	= 2[us]
Clipped	= FALSE
Scans	= 16
Total_Scans	= 16
Relaxation_Delay	= 4[s]
Recvr_Gain	= 42
Temp_Get	= 16.9[dC]
X_90_Width	= 5.85[us]
X_Acq_Time	= 4.37256192[s]
X_Angle	= 45[deg]
X_Atn	= 1.3[dB]
X_Pulse	= 2.925[us]
Irr_Mode	= Off
Tri_Mode	= Off
Dante_Loop	= 400
Dante_Presat	= FALSE
Decimation_Rate	= 0
Experiment_Path	= c:\Program Files\JE
Initial_Wait	= 1[s]
Phase	= {0, 90, 270, 180, 1
Presat_Time	= 4[s]
Presat_Time_Flag	= FALSE
Relaxation_Delay_Calc	= 0[s]
Relaxation_Delay_Temp	= 4[s]
Repetition_Time	= 8.37256192[s]

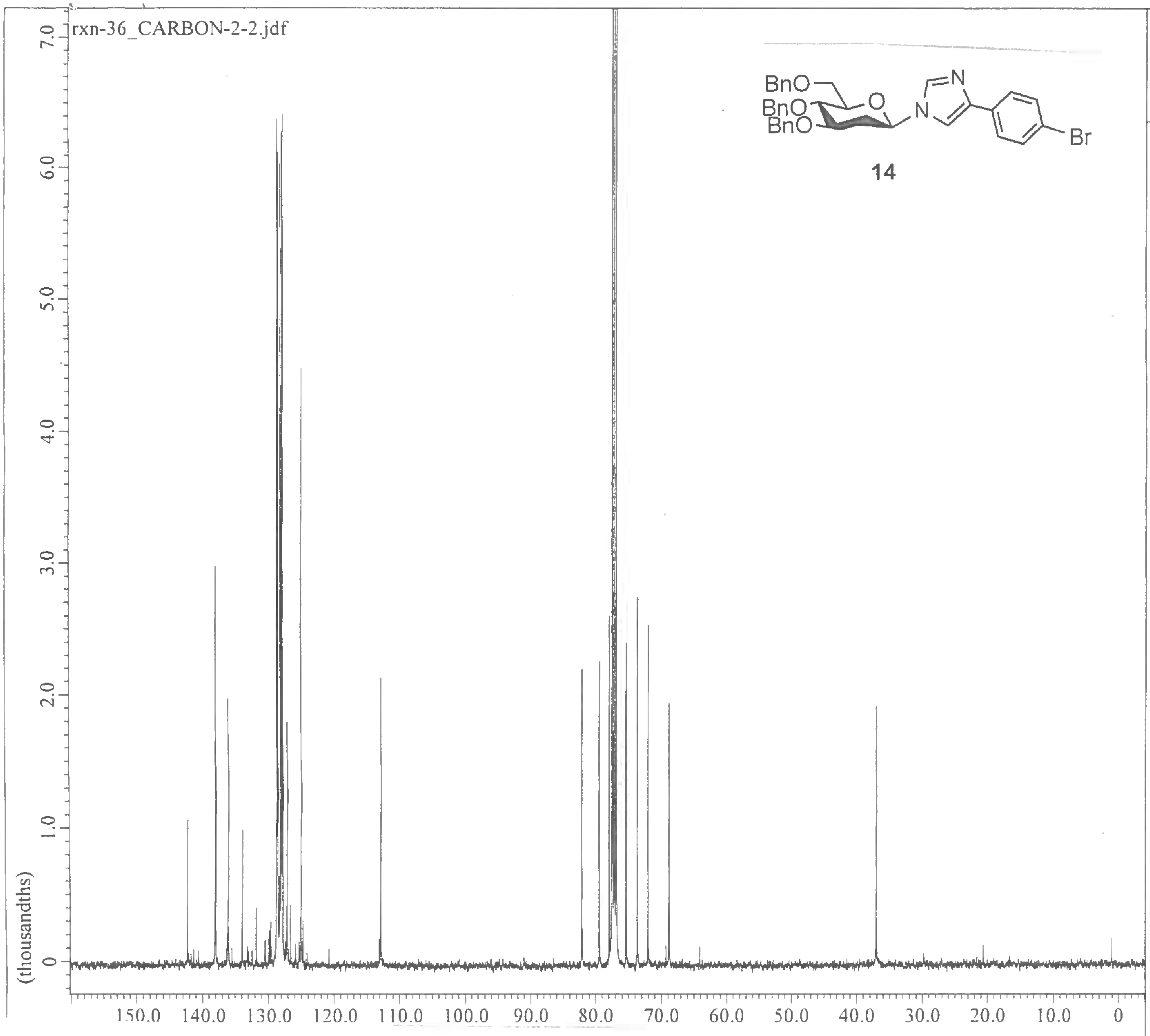

14

JEOL

Filename	= rxn-36_CARBON-2-
Author	= decastm
Experiment	= carbon.jxp
Sample_Id	= rxn-36
Solvent	= CHLOROFORM-D
Actual_Start_Time	= 20-MAY-2019 12:3
Revision_Time	= 21-MAY-2019 14:2
Data_Format	= 1D COMPLEX
Dim_Size	= 26214
X_Domain	= Carbon13
Dim_Title	= Carbon13
Dim_Units	= [ppm]
Dimensions	= X
Site	= Farmingdale Stat
Spectrometer	= JNM-ECZ400S/L1
Field_Strength	= 9.389766[T] (400
X_Acq_Duration	= 1.03809024[s]
X_Domain	= Carbon13
X_Freq	= 100.52530333[MHz
X_Offset	= 100[ppm]
X_Points	= 32768
X_Prescans	= 4
X_Resolution	= 0.96330739[Hz]
X_Sweep	= 31.56565657[kHz]
X_Sweep_Clipped	= 25.25252525[kHz]
Irr_Domain	= Proton
Irr_Freq	= 399.78219838[MHz
Irr_Offset	= 5[ppm]
Blanking	= 5[us]
Clipped	= FALSE
Scans	= 5000
Total_Scans	= 5000
Relaxation_Delay	= 2[s]
Recvr_Gain	= 52
Temp_Get	= 17.7[dC]
X_90_Width	= 11.73[us]
X_Acq_Time	= 1.03809024[s]
X_Angle	= 30[deg]
X_Atn	= 7.9[dB]
X_Pulse	= 3.91[us]
Irr_Atn_Dec	= 27[dB]
Irr_Atn_Dec_Calc	= 27[dB]
Irr_Atn_Dec_Default_Calc	= 27[dB]
Irr_Atn_Noe	= 27[dB]
Irr_Dec_Bandwidth_Hz	= 4.7826087[kHz]
Irr_Dec_Bandwidth_Ppm	= 11.96303566[ppm]
Irr_Dec_Freq	= 399.78219838[MHz
Irr_Dec_Merit_Factor	= 2.2
Irr_Decoupling	= TRUE
Irr_Noe	= TRUE
Irr_Noise	= WALTZ
Irr_Offset_Default	= 5[ppm]
Irr_Pwidth	= 0.115[ms]
Irr_Pwidth_Default	= 0.115[ms]
Irr_Pwidth_Default_Calc	= 0.115[ms]
Irr_Pwidth_Templ	= 0.115[ms]
Irr_Wurst	= FALSE
Decimation_Rate	= 0
Experiment_Path	= c:\Program Files
Initial_Wait	= 1[s]
Noe_Time	= 2[s]

mdc-36

Qtof_72038 68 (2.572) AM (Cen,5, 80.00, Ar,14000.0,734.47,0.70,LS 5); Sm (SG, 2x5.00); Cm (67:72) 1: TOF MS ES+

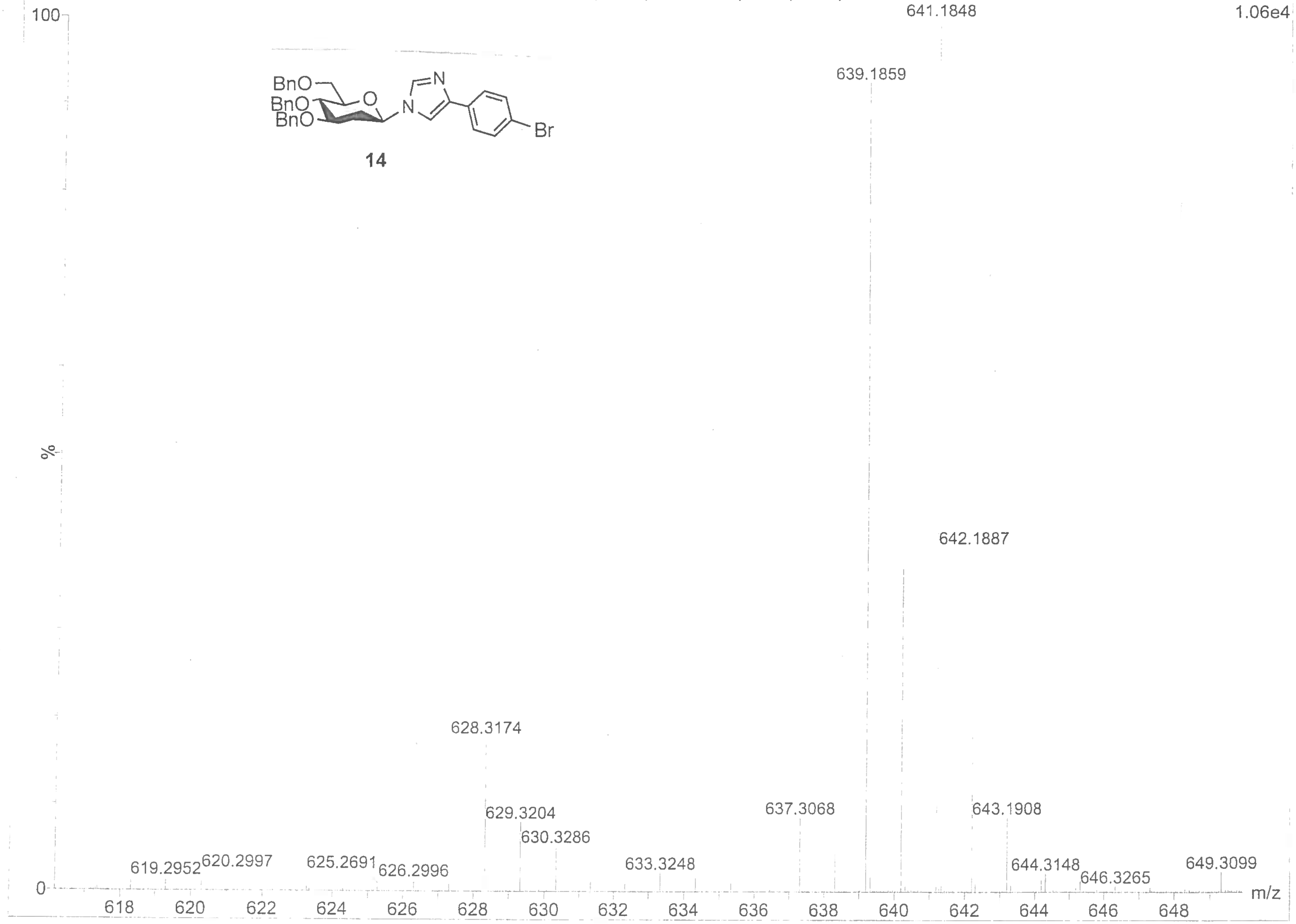

Elemental Composition Report

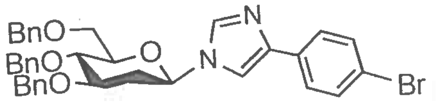

14

Single Mass Analysis

Tolerance = 5.0 PPM / DBE: min = -1.5, max = 100.0
Element prediction: Off
Number of isotope peaks used for i-FIT = 3

Monoisotopic Mass, Even Electron Ions
1042 formula(e) evaluated with 5 results within limits (up to 50 closest results for each mass)
Elements Used:
C: 0-200 H: 0-200 N: 0-5 O: 0-5 Na: 0-1 Br: 0-1

mdc-36
Qtof_72038 68 (2.572) AM (Cen,5, 80.00, Ar,14000.0,734.47,0.70,LS 5); Sm (SG, 2x5.00); Cm (67:72)

1: TOF MS ES+
1.06e+004

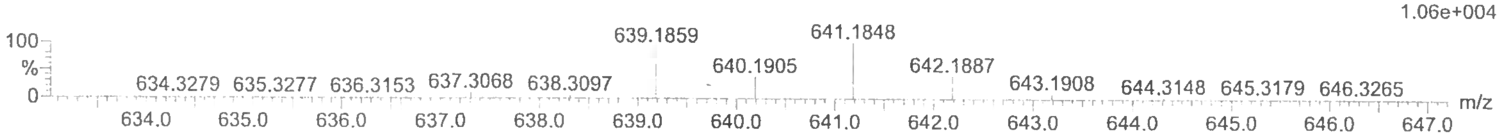

Minimum:				-1.5		
Maximum:		5.0	5.0	100.0		
Mass	Calc. Mass	mDa	PPM	DBE	i-FIT	Formula
639.1859	639.1858	0.1	0.2	19.5	2.2	C36 H36 N2 O4 Br
	639.1861	-0.2	-0.3	39.5	4182.2	C49 H23 N2
	639.1875	-1.6	-2.5	20.5	13.9	C39 H37 O2 Na Br
	639.1837	2.2	3.4	36.5	4216.3	C47 H24 N2 Na
	639.1834	2.5	3.9	16.5	3.1	C34 H37 N2 O4 Na Br

206 s-2_PROTON-4-4.jdf

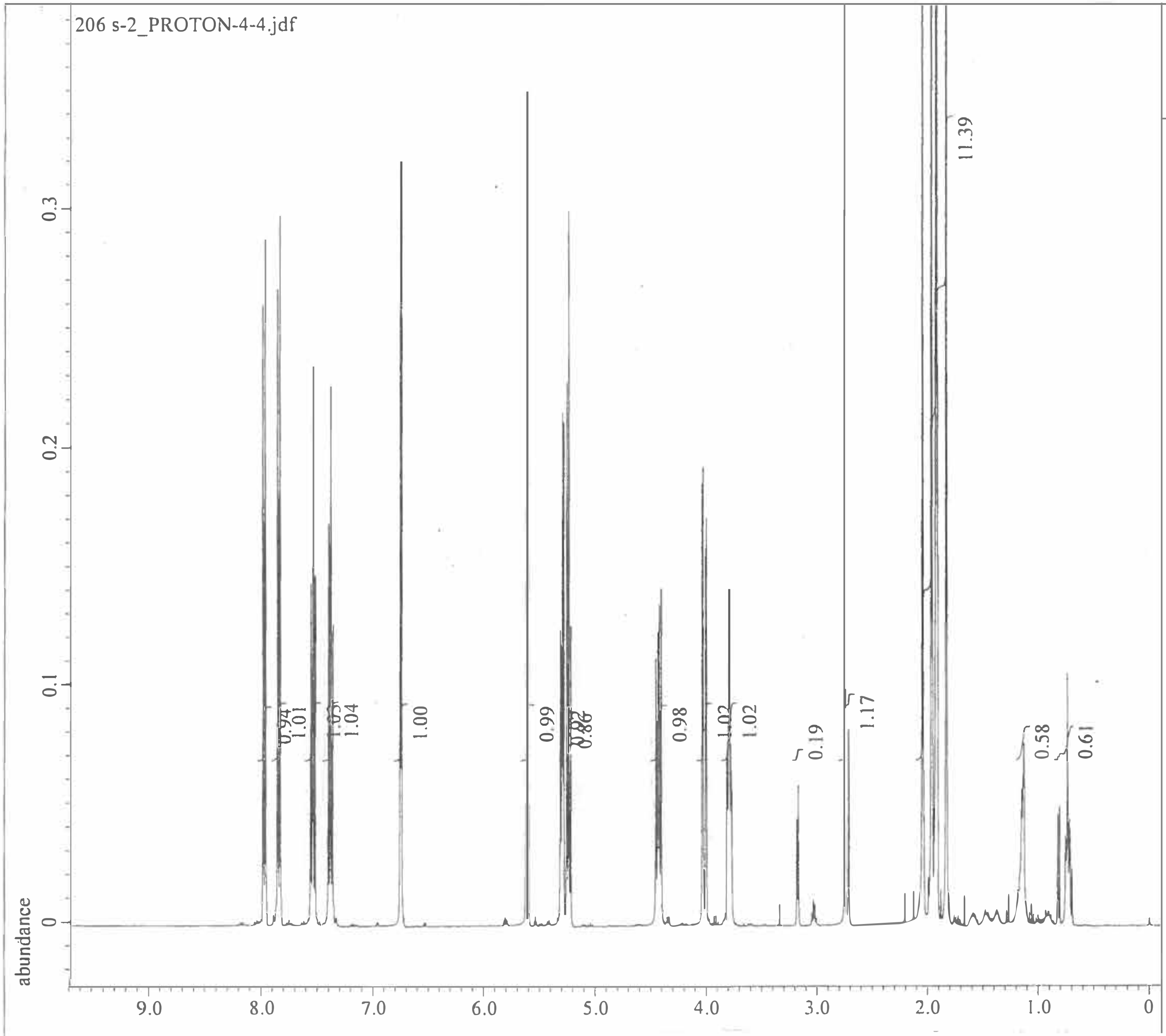

JEOL

```
---- PROCESSING PARAMETERS ----
sexp( 0.2[Hz], 0.0[s] )
trapezoid3( 0[%], 80[%], 100[%] )
zerofill( 1 )
fft( 1, TRUE, TRUE )
machinephase
ppm
thresh( 2[%], 1 )
peak_pick( 0[Hz], 0.1[ppm], Both, 0[Hz] )
norm_smallest_int( 1.0, 0[Hz], 25[Hz] )

Derived from: 206 s-2_PROTON-4-1.jdf
```

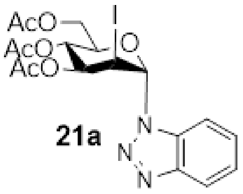

21a

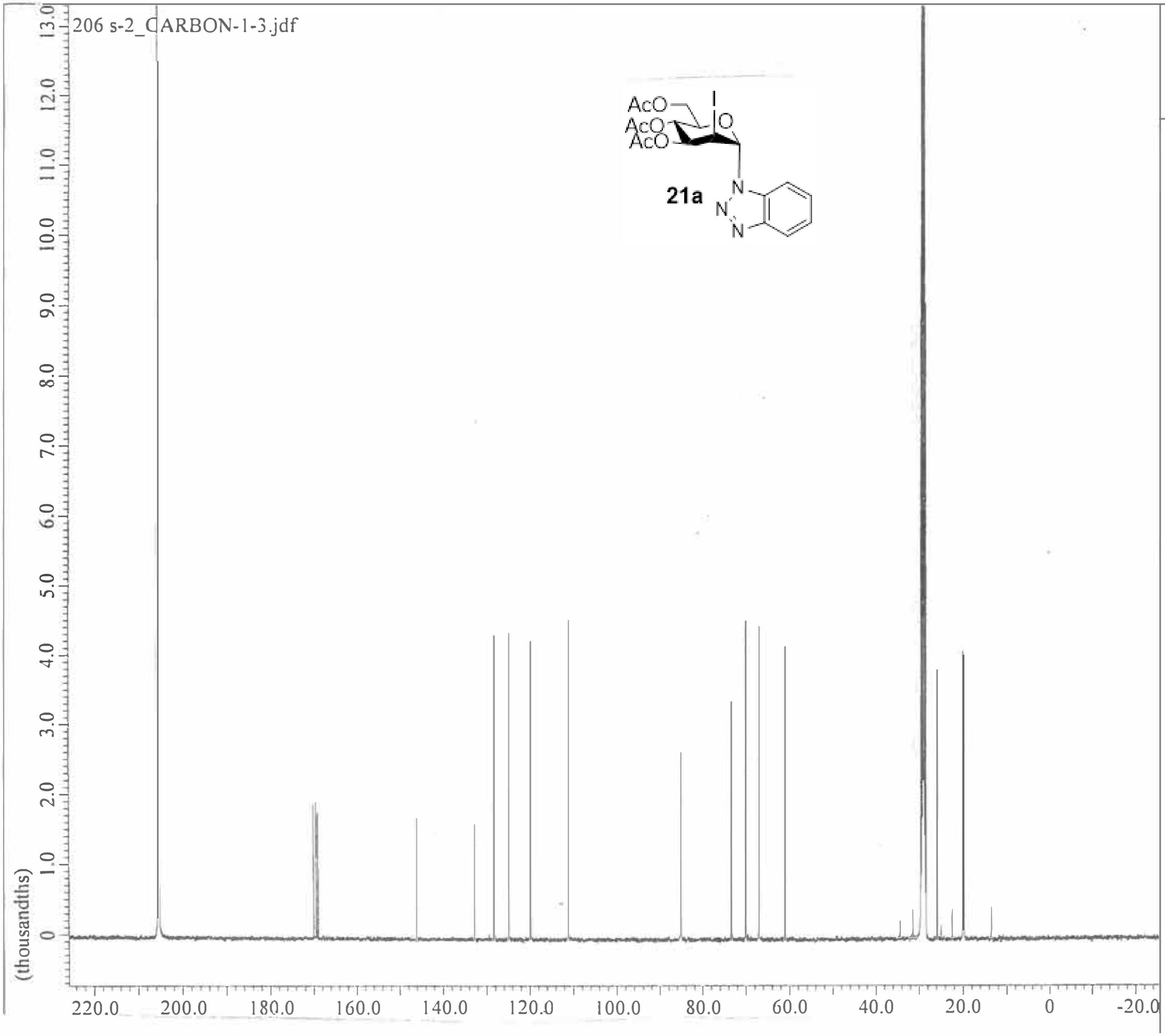


```
---- PROCESSING PARAMETERS ----
sexp( 2.0[Hz], 0.0[s] )
trapezoid3( 0[%], 80[%], 100[%] )
zerofill( 1 )
fft( 1, TRUE, TRUE )
machinephase
ppm
thresh( 5[%], 1 )
peak_pick( 0[Hz], 0.1[ppm], Peaks, 0[Hz] )

Derived from: 206 s-2_CARBON-1-1.jdf
```

Michael De Castro 206 S-2

SYNAPTG2-Si#UGA305
13:01:01

Synapt_18957 32 (0.637)

1: TOF MS ES+
1.24e5

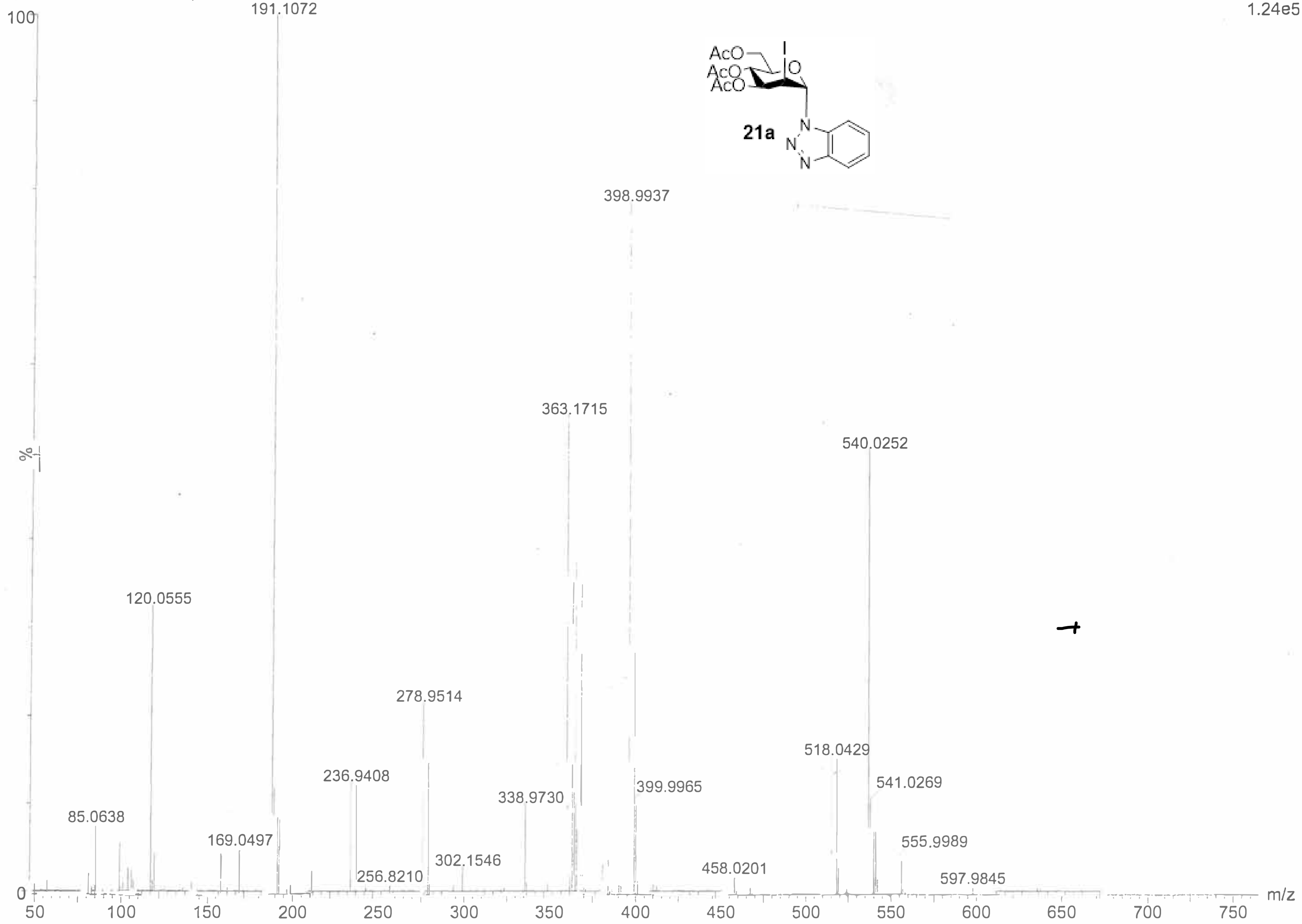

21a

Elemental Composition Report

Single Mass Analysis

Tolerance = 5.0 PPM / DBE: min = -50.0, max = 100.0
Element prediction: Off
Number of isotope peaks used for i-FIT = 9

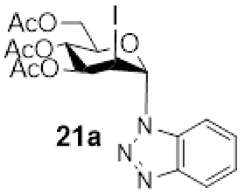

21a

Monoisotopic Mass, Even Electron Ions
193 formula(e) evaluated with 1 results within limits (up to 10 best isotopic matches for each mass)
Elements Used:
C: 0-90 H: 0-130 N: 2-4 O: 6-8 Na: 0-1 I: 1-1

Mass	Calc. Mass	mDa	PPM	DBE	i-FIT	Norm	Conf(%)	Formula
Minimum:				-50.0				
Maximum:		5.0	5.0	100.0				
518.0429	518.0424	0.5	1.0	9.5	105.0	n/a	n/a	C18 H21 N3 O7 I

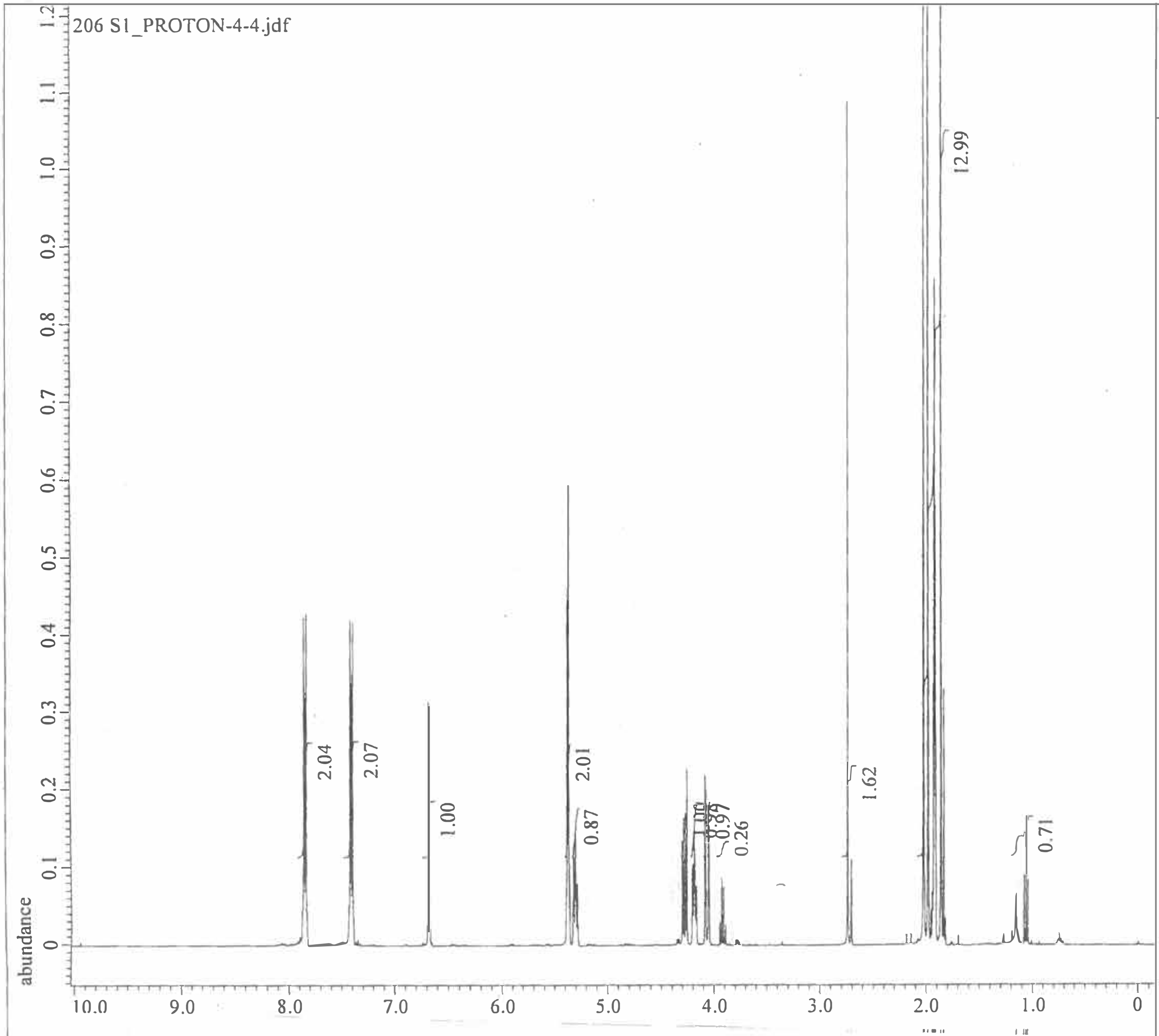

JEOL

```
---- PROCESSING PARAMETERS ----
sexp( 0.2[Hz], 0.0[s] )
trapezoid3( 0[%], 80[%], 100[%] )
zerofill( 1 )
fft( 1, TRUE, TRUE )
machinephase
ppm
thresh( 2[%], 1 )
peak_pick( 0[Hz], 0.1[ppm], Both, 0[Hz] )
norm_smallest_int( 1.0, 0[Hz], 25[Hz] )

Derived from: 206 S1_PROTON-4-1.jdf
```

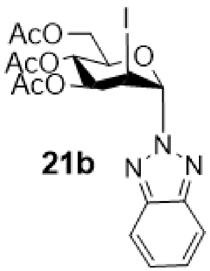

21b

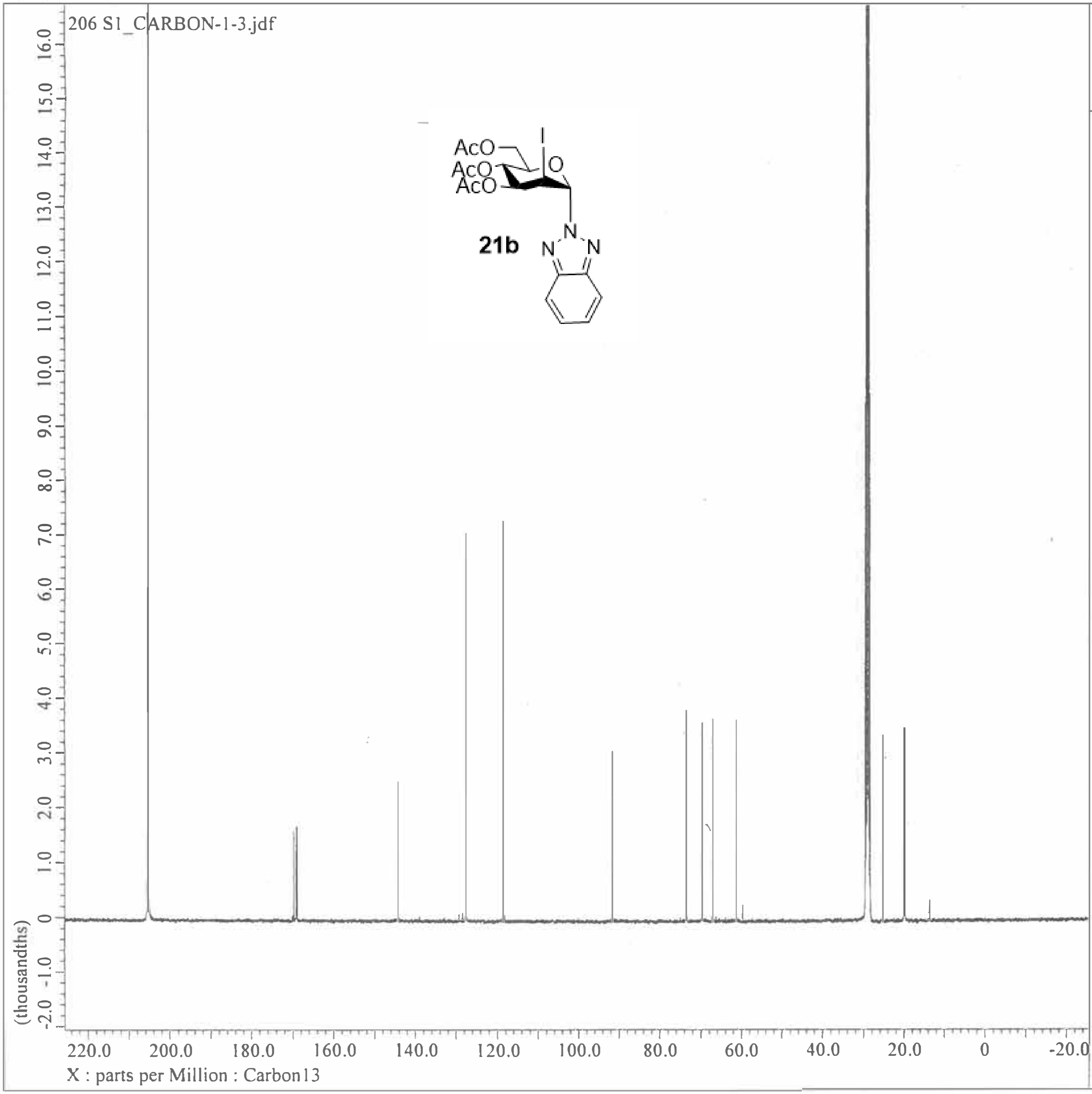

---- PROCESSING PARAMETERS ----
sexp(2.0[Hz], 0.0[s])
trapezoid3(0[%], 80[%], 100[%])
zerofill(1)
fft(1, TRUE, TRUE)
machinephase
ppm
thresh(5[%], 1)

Derived from: 206 S1_CARBON-1-1.jdf

Michael De Castro 206 S-2

SYNAPTG2-Si#UGA305
13:01:01

Synapt_18957 32 (0.637)

1: TOF MS ES+
1.24e5

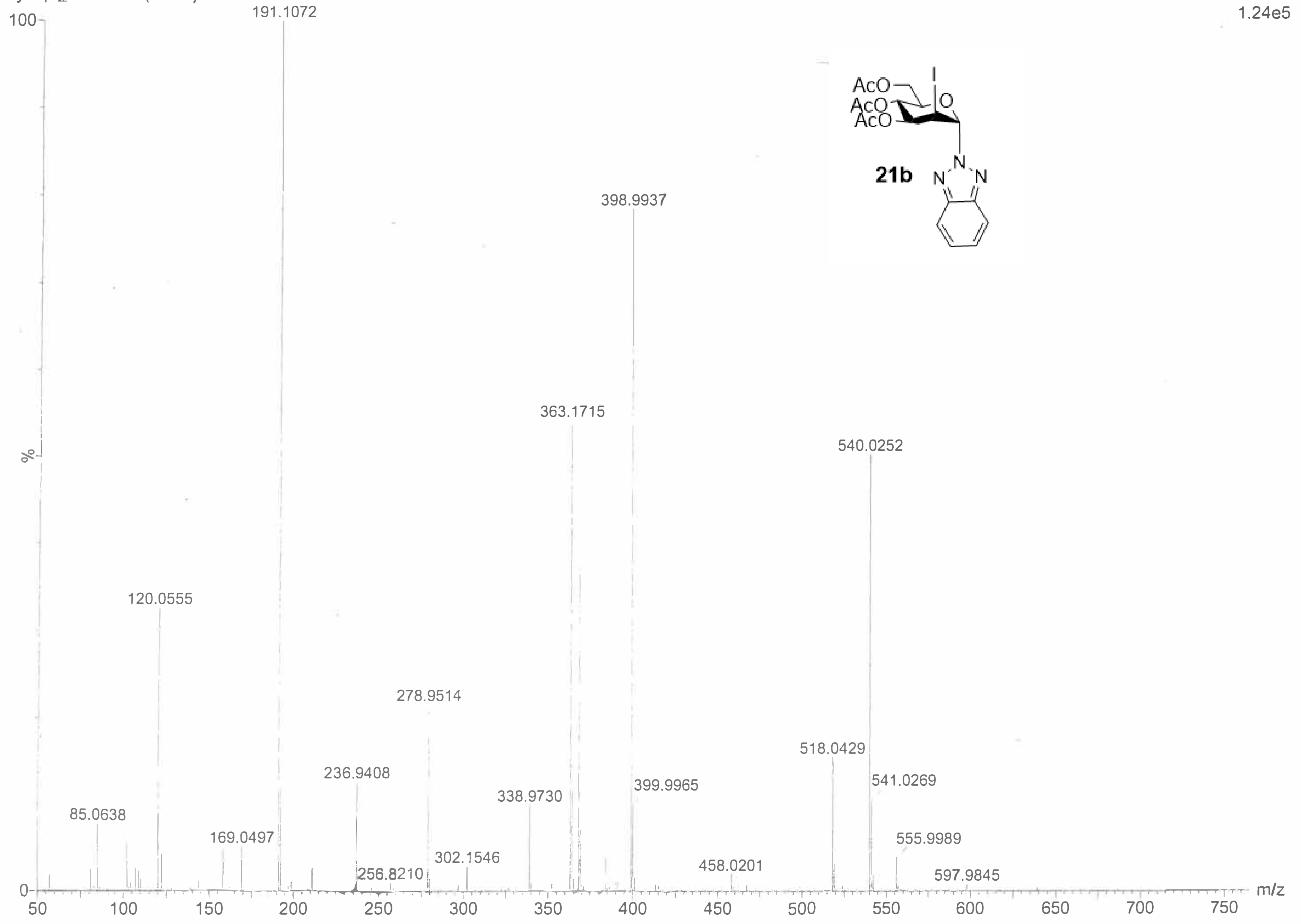

Elemental Composition Report

Single Mass Analysis

Tolerance = 5.0 PPM / DBE: min = -50.0, max = 100.0
Element prediction: Off
Number of isotope peaks used for i-FIT = 9

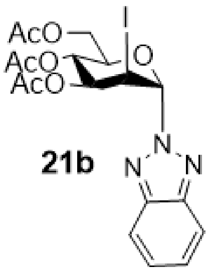

21b

Monoisotopic Mass, Even Electron Ions
193 formula(e) evaluated with 1 results within limits (up to 10 best isotopic matches for each mass)
Elements Used:
C: 0-90 H: 0-130 N: 2-4 O: 6-8 Na: 0-1 I: 1-1

Minimum:				-50.0				
Maximum:		5.0	5.0	100.0				
Mass	Calc. Mass	mDa	PPM	DBE	i-FIT	Norm	Conf(%)	Formula
518.0429	518.0424	0.5	1.0	9.5	105.0	n/a	n/a	C18 H21 N3 O7 I

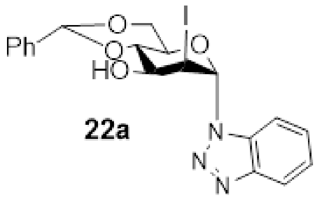

22a

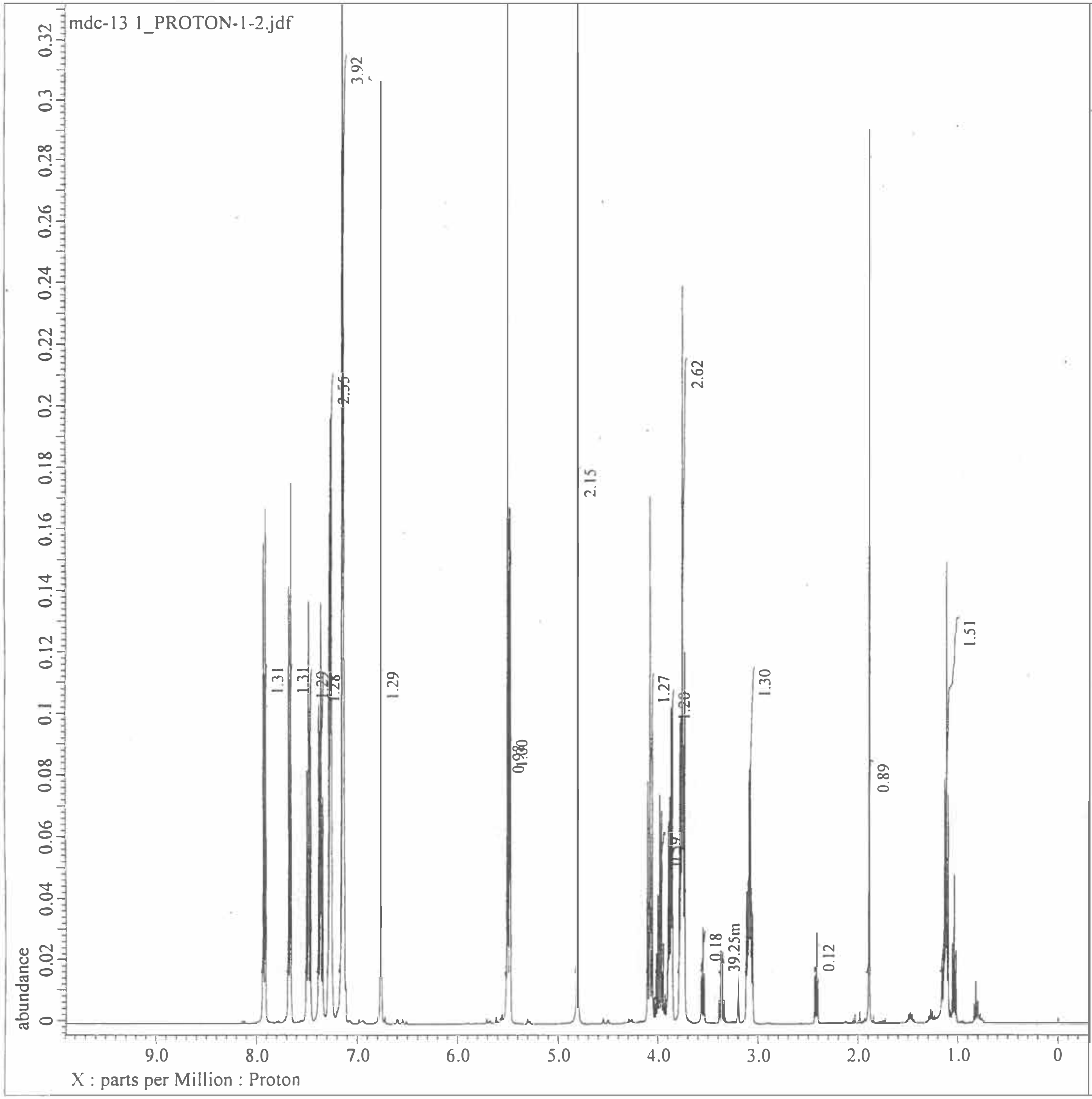

Filename	= mdc-13 1_PROTON-1-2
Author	= decastm
Experiment	= proton.jxp
Sample_Id	= mdc-13 1
Solvent	= METHANOL-D4
Actual_Start_Time	= 25-JUN-2019 11:37:3
Revision_Time	= 25-JUN-2019 11:43:0
Data_Format	= 1D COMPLEX
Dim_Size	= 26214
X_Domain	= Proton
Dim_Title	= Proton
Dim_Units	= [ppm]
Dimensions	= X
Site	= Farmingdale State C
Spectrometer	= JNM-ECZ400S/L1
Field_Strength	= 9.389766[T] (400[MH
X_Acq_Duration	= 4.37256192[s]
X_Domain	= Proton
X_Freq	= 399.78219838[MHz]
X_Offset	= 5[ppm]
X_Points	= 32768
X_Prescans	= 0
X_Resolution	= 0.22869888[Hz]
X_Sweep	= 7.4940048[kHz]
X_Sweep_Clipped	= 5.99520384[kHz]
Irr_Domain	= Proton
Irr_Freq	= 399.78219838[MHz]
Irr_Offset	= 5[ppm]
Tri_Domain	= Proton
Tri_Freq	= 399.78219838[MHz]
Tri_Offset	= 5[ppm]
Blanking	= 2[us]
Clipped	= FALSE
Scans	= 16
Total_Scans	= 16
Relaxation_Delay	= 4[s]
Recvr_Gain	= 42
Temp_Get	= 17.1[dC]
X_90_Width	= 5.85[us]
X_Acq_Time	= 4.37256192[s]
X_Angle	= 45[deg]
X_Atn	= 1.3[dB]
X_Pulse	= 2.925[us]
Irr_Mode	= Off
Tri_Mode	= Off
Dante_Loop	= 400
Dante_Presat	= FALSE
Decimation_Rate	= 0
Experiment_Path	= c:\Program Files\JE
Initial_Wait	= 1[s]
Phase	= {0, 90, 270, 180, 1
Presat_Time	= 4[s]
Presat_Time_Flag	= FALSE
Relaxation_Delay_Calc	= 0[s]
Relaxation_Delay_Temp	= 4[s]
Repetition_Time	= 8.37256192[s]

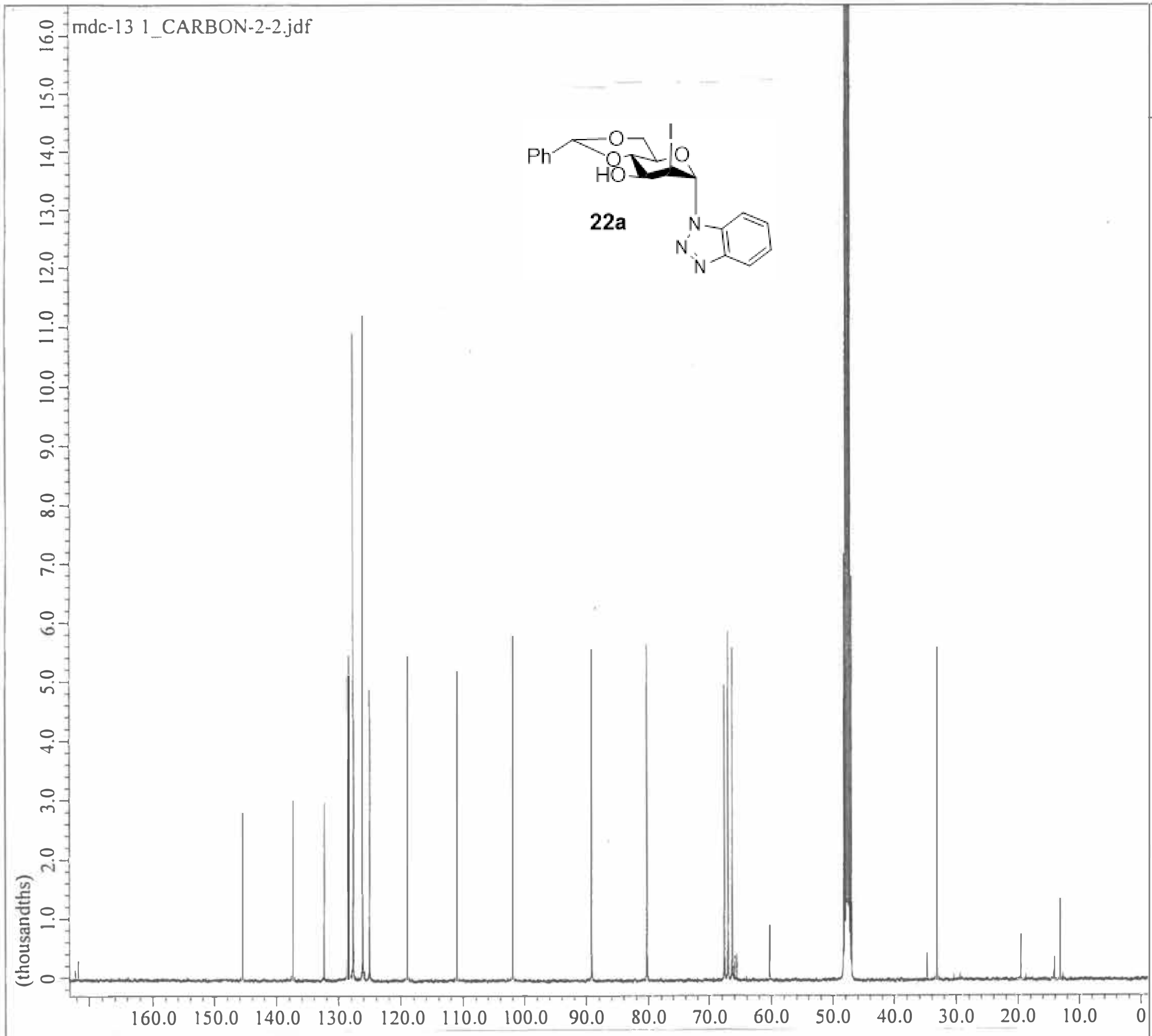

JEOL

Filename	= mdc-13 1_CARBON-
Author	= decastm
Experiment	= carbon.jxp
Sample_Id	= mdc-13 1
Solvent	= METHANOL-D4
Actual_Start_Time	= 25-JUN-2019 16:4
Revision_Time	= 1-JUL-2019 15:1
Data_Format	= 1D COMPLEX
Dim_Size	= 26214
X_Domain	= Carbon13
Dim_Title	= Carbon13
Dim_Units	= [ppm]
Dimensions	= X
Site	= Farmingdale Stat
Spectrometer	= JNM-ECZ400S/L1
Field_Strength	= 9.389766[T] (400
X_Acq_Duration	= 1.03809024[s]
X_Domain	= Carbon13
X_Freq	= 100.52530333[MHz
X_Offset	= 100[ppm]
X_Points	= 32768
X_Prescans	= 4
X_Resolution	= 0.96330739[Hz]
X_Sweep	= 31.56565657[kHz]
X_Sweep_Clipped	= 25.25252525[kHz]
Irr_Domain	= Proton
Irr_Freq	= 399.78219838[MHz
Irr_Offset	= 5[ppm]
Blanking	= 5[us]
Clipped	= FALSE
Scans	= 5000
Total_Scans	= 5000
Relaxation_Delay	= 2[s]
Recvr_Gain	= 52
Temp_Get	= 17.1[dC]
X_90_Width	= 11.73[us]
X_Acq_Time	= 1.03809024[s]
X_Angle	= 30[deg]
X_Atn	= 7.9[dB]
X_Pulse	= 3.91[us]
Irr_Atn_Dec	= 27[dB]
Irr_Atn_Dec_Calc	= 27[dB]
Irr_Atn_Dec_Default_Calc	= 27[dB]
Irr_Atn_Noe	= 27[dB]
Irr_Dec_Bandwidth_Hz	= 4.7826087[kHz]
Irr_Dec_Bandwidth_Ppm	= 11.96303566[ppm]
Irr_Dec_Freq	= 399.78219838[MHz
Irr_Dec_Merit_Factor	= 2.2
Irr_Decoupling	= TRUE
Irr_Noe	= TRUE
Irr_Noise	= WALTZ
Irr_Offset_Default	= 5[ppm]
Irr_Pwidth	= 0.115[ms]
Irr_Pwidth_Default	= 0.115[ms]
Irr_Pwidth_Default_Calc	= 0.115[ms]
Irr_Pwidth_Templ	= 0.115[ms]
Irr_Wurst	= FALSE
Decimation_Rate	= 0
Experiment_Path	= c:\Program Files
Initial_Wait	= 1[s]
Noe_Time	= 2[s]

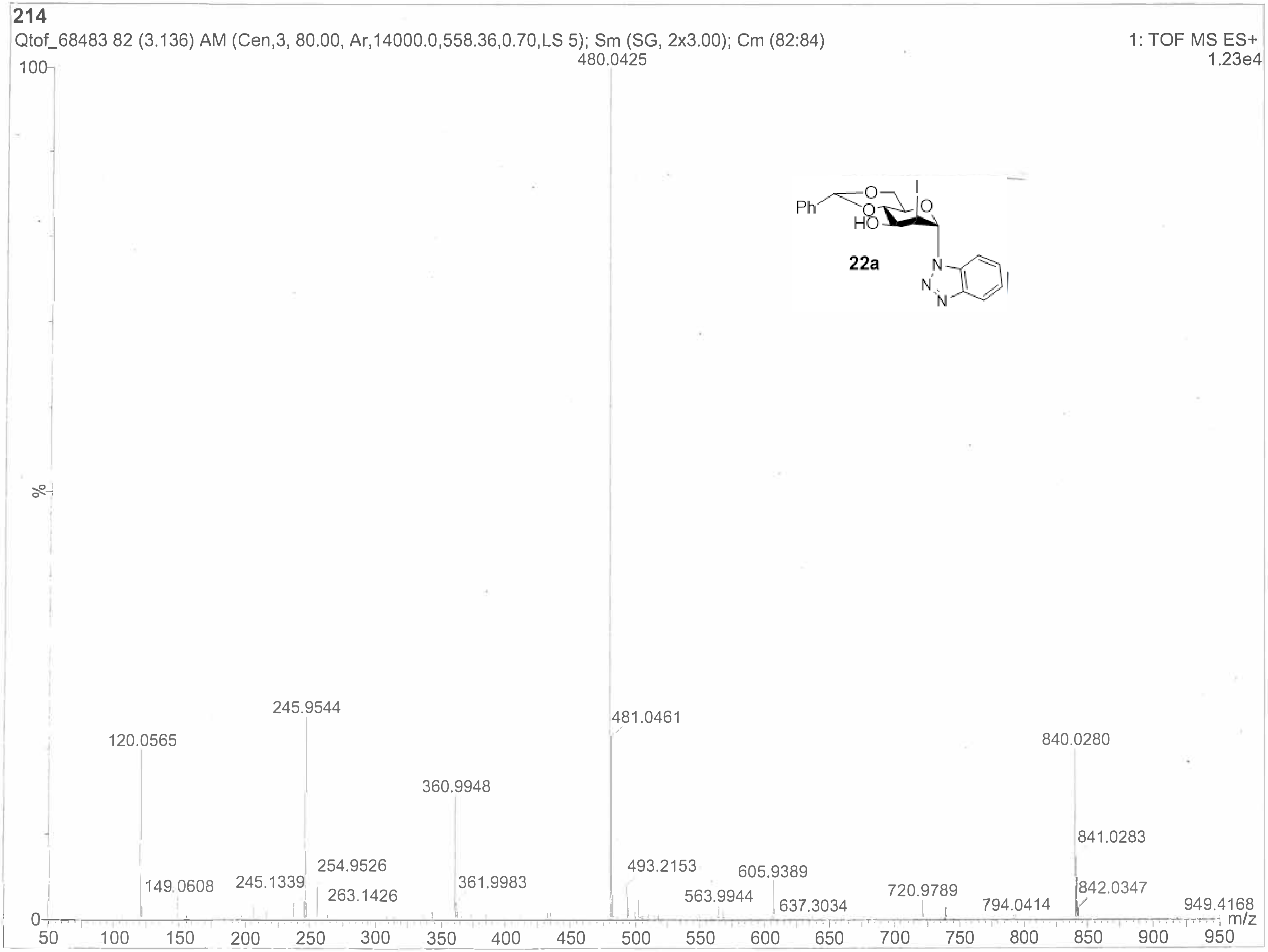
214
Qtof_68483 82 (3.136) AM (Cen,3, 80.00, Ar,14000.0,558.36,0.70,LS 5); Sm (SG, 2x3.00); Cm (82:84)
1: TOF MS ES+
1.23e4
100
%
0
480.0425
245.9544
120.0565
481.0461
840.0280
360.9948
841.0283
254.9526
493.2153
605.9389
149.0608
245.1339
361.9983
263.1426
563.9944
637.3034
720.9789
842.0347
794.0414
949.4168
m/z
50
100
150
200
250
300
350
400
450
500
550
600
650
700
750
800
850
900
950
22a
Ph
O
HO
I
N
N
N

Elemental Composition Report

Single Mass Analysis

Tolerance = 5.0 PPM / DBE: min = -1.5, max = 100.0
Element prediction: Off
Number of isotope peaks used for i-FIT = 3

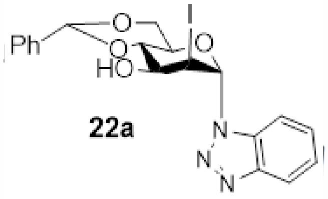

22a

Monoisotopic Mass, Even Electron Ions
833 formula(e) evaluated with 4 results within limits (up to 50 closest results for each mass)
Elements Used:
C: 0-200 H: 0-200 N: 0-5 O: 0-6 Na: 0-1 I: 0-1

214
Qtof_68483 82 (3.136) AM (Cen,3, 80.00, Ar,14000.0,558.36,0.70,LS 5); Sm (SG, 2x3.00); Cm (82:84)

1: TOF MS ES+
1.23e+004

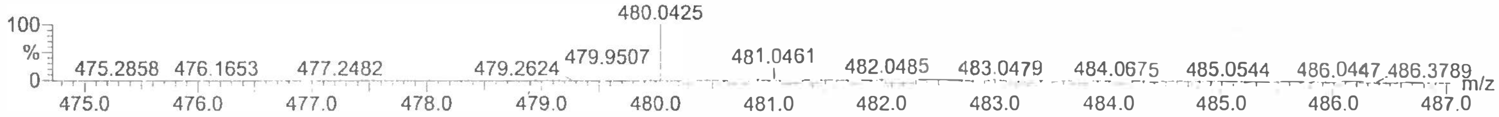

Minimum:				-1.5		
Maximum:		5.0	5.0	100.0		
Mass	Calc. Mass	mDa	PPM	DBE	i-FIT	Formula
480.0425	480.0425	0.0	0.0	32.5	750.0	C35 H7 N O Na
	480.0420	0.5	1.0	11.5	5.6	C19 H19 N3 O4 I
	480.0437	-1.2	-2.5	12.5	35.6	C22 H20 N O2 Na I
	480.0409	1.6	3.3	31.5	604.2	C32 H6 N3 O3

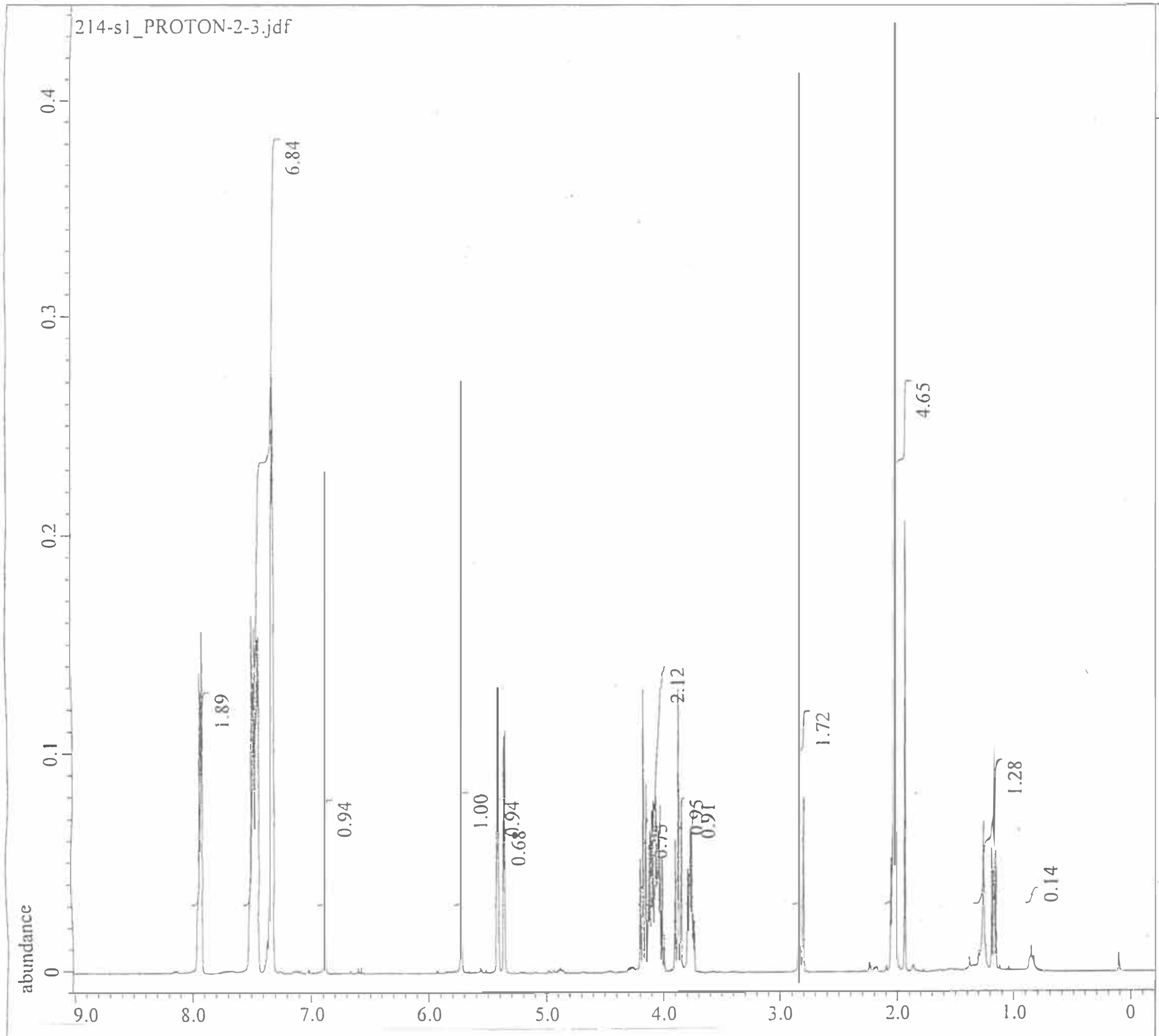

JEOL

```
---- PROCESSING PARAMETERS ----
sexp( 0.2[Hz], 0.0[s] )
trapezoid3( 0[%], 80[%], 100[%] )
zerofill( 1 )
fft( 1, TRUE, TRUE )
machinephase
ppm
thresh( 2[%], 1 )
peak_pick( 0[Hz], 0.1[ppm], Both, 0[Hz] )
norm_smallest_int( 1.0, 0[Hz], 25[Hz] )
clip( 1.3, FALSE )

Derived from: 214-s1_PROTON-2-1.jdf
```

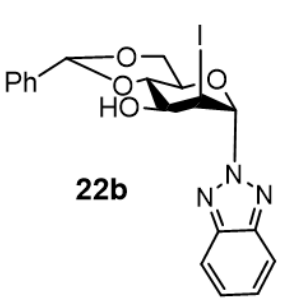

22b

214-s2_CARBON-3-3.jdf

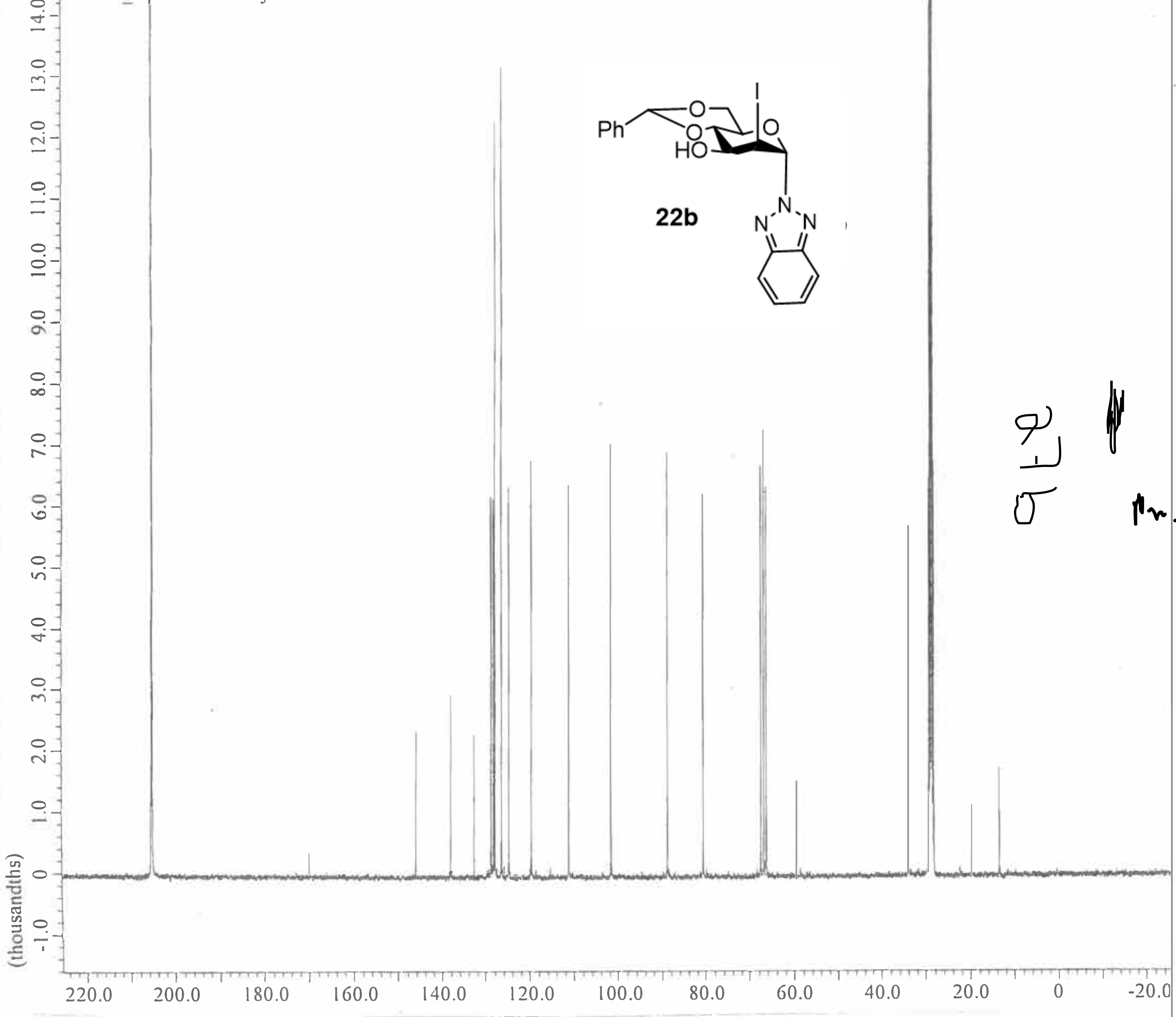

JEOL

```
---- PROCESSING PARAMETERS ----
sexp( 2.0[Hz], 0.0[s] )
trapezoid3( 0[%], 80[%], 100[%] )
zerofill( 1 )
fft( 1, TRUE, TRUE )
machinephase
ppm
thresh( 5[%], 1 )
peak_pick( 0[Hz], 0.1[ppm], Peaks, 0[Hz] )

Derived from: 214-s2_CARBON-3-1.jdf
```

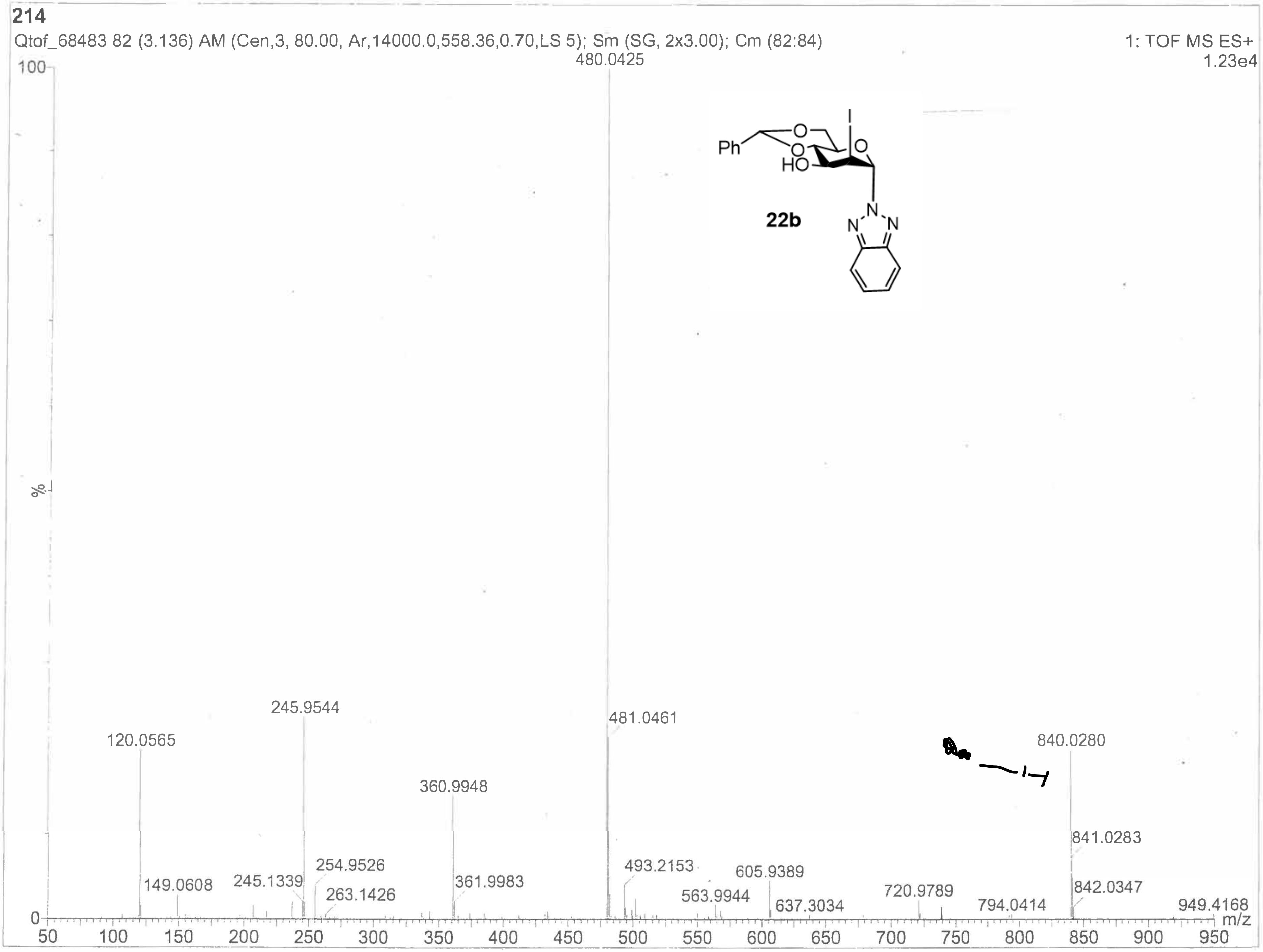
214
Qtof_68483 82 (3.136) AM (Cen,3, 80.00, Ar,14000.0,558.36,0.70,LS 5); Sm (SG, 2x3.00); Cm (82:84)
1: TOF MS ES+
1.23e4
100
%
0
480.0425
481.0461
245.9544
120.0565
840.0280
360.9948
841.0283
254.9526
493.2153
605.9389
149.0608
245.1339
361.9983
842.0347
263.1426
563.9944
720.9789
637.3034
794.0414
949.4168
m/z
50
100
150
200
250
300
350
400
450
500
550
600
650
700
750
800
850
900
950
Ph
O
O
HO
O
I
N
N
N
22b

Elemental Composition Report

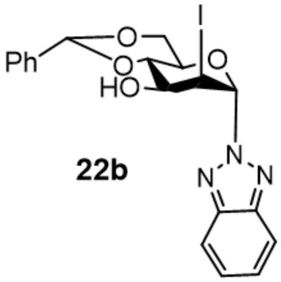

22b

Single Mass Analysis

Tolerance = 5.0 PPM / DBE: min = -1.5, max = 100.0
Element prediction: Off
Number of isotope peaks used for i-FIT = 3

Monoisotopic Mass, Even Electron Ions
833 formula(e) evaluated with 4 results within limits (up to 50 closest results for each mass)
Elements Used:
C: 0-200 H: 0-200 N: 0-5 O: 0-6 Na: 0-1 I: 0-1

214
Qtof_68483 82 (3.136) AM (Cen,3, 80.00, Ar,14000.0,558.36,0.70,LS 5); Sm (SG, 2x3.00); Cm (82:84)

1: TOF MS ES+
1.23e+004

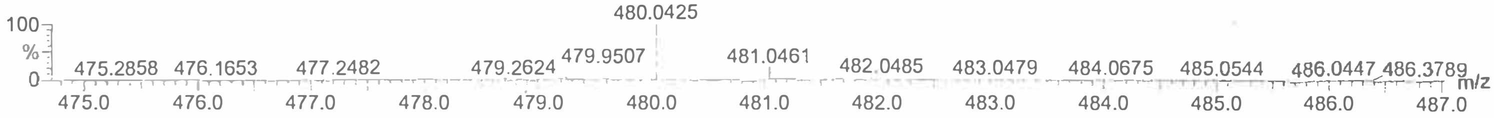

Minimum:				-1.5		
Maximum:		5.0	5.0	100.0		

Mass	Calc. Mass	mDa	PPM	DBE	i-FIT	Formula
480.0425	480.0425	0.0	0.0	32.5	750.0	C35 H7 N O Na
	480.0420	0.5	1.0	11.5	5.6	C19 H19 N3 O4 I
	480.0437	-1.2	-2.5	12.5	35.6	C22 H20 N O2 Na I
	480.0409	1.6	3.3	31.5	604.2	C32 H6 N3 O3

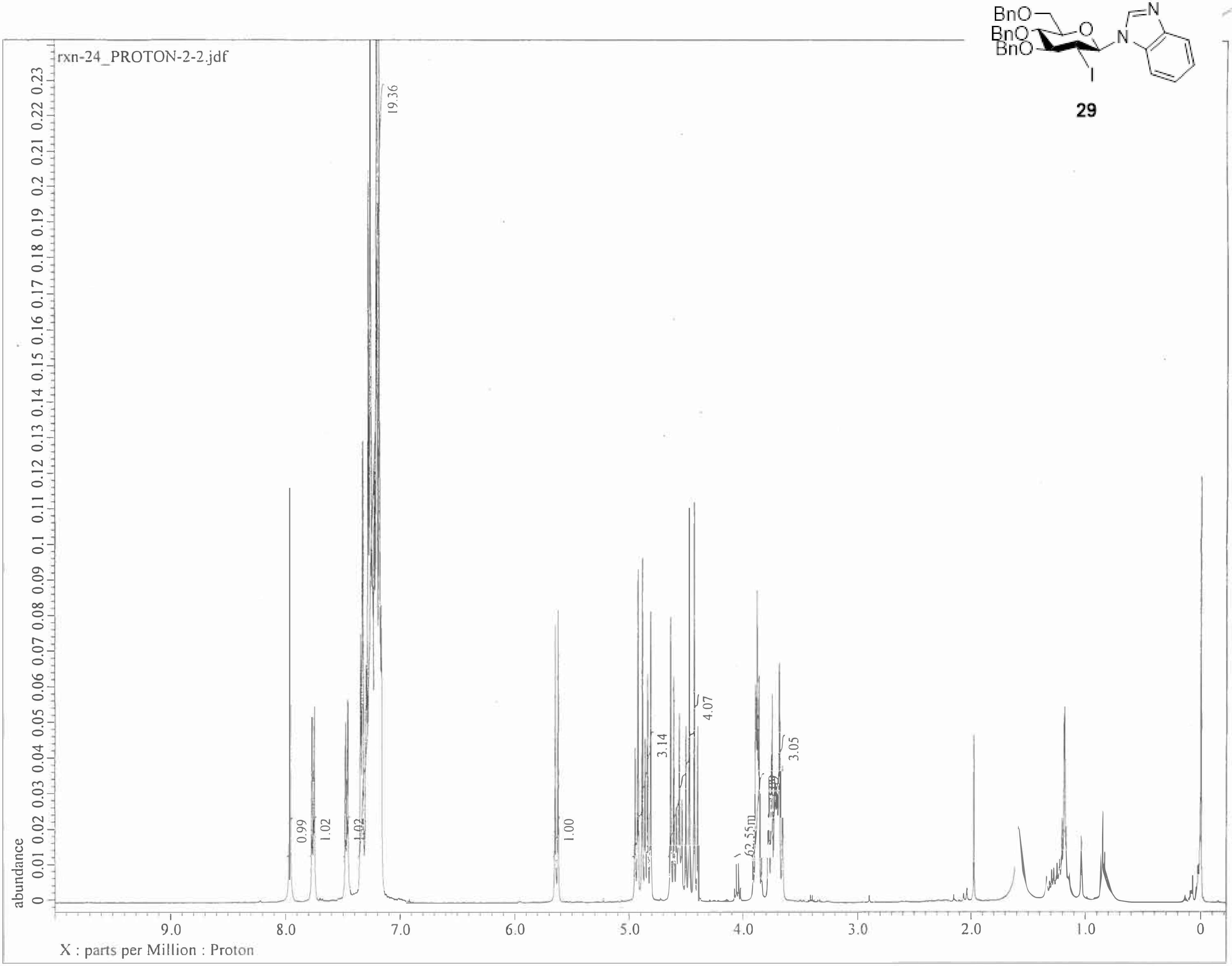

rxn-24_PROTON-2-2.jdf
abundance
0 0.01 0.02 0.03 0.04 0.05 0.06 0.07 0.08 0.09 0.1 0.11 0.12 0.13 0.14 0.15 0.16 0.17 0.18 0.19 0.2 0.21 0.22 0.23
0.99
1.02
1.02
19.36
1.00
3.14
4.07
62.55m
3.05
9.0 8.0 7.0 6.0 5.0 4.0 3.0 2.0 1.0 0
X : parts per Million : Proton

29

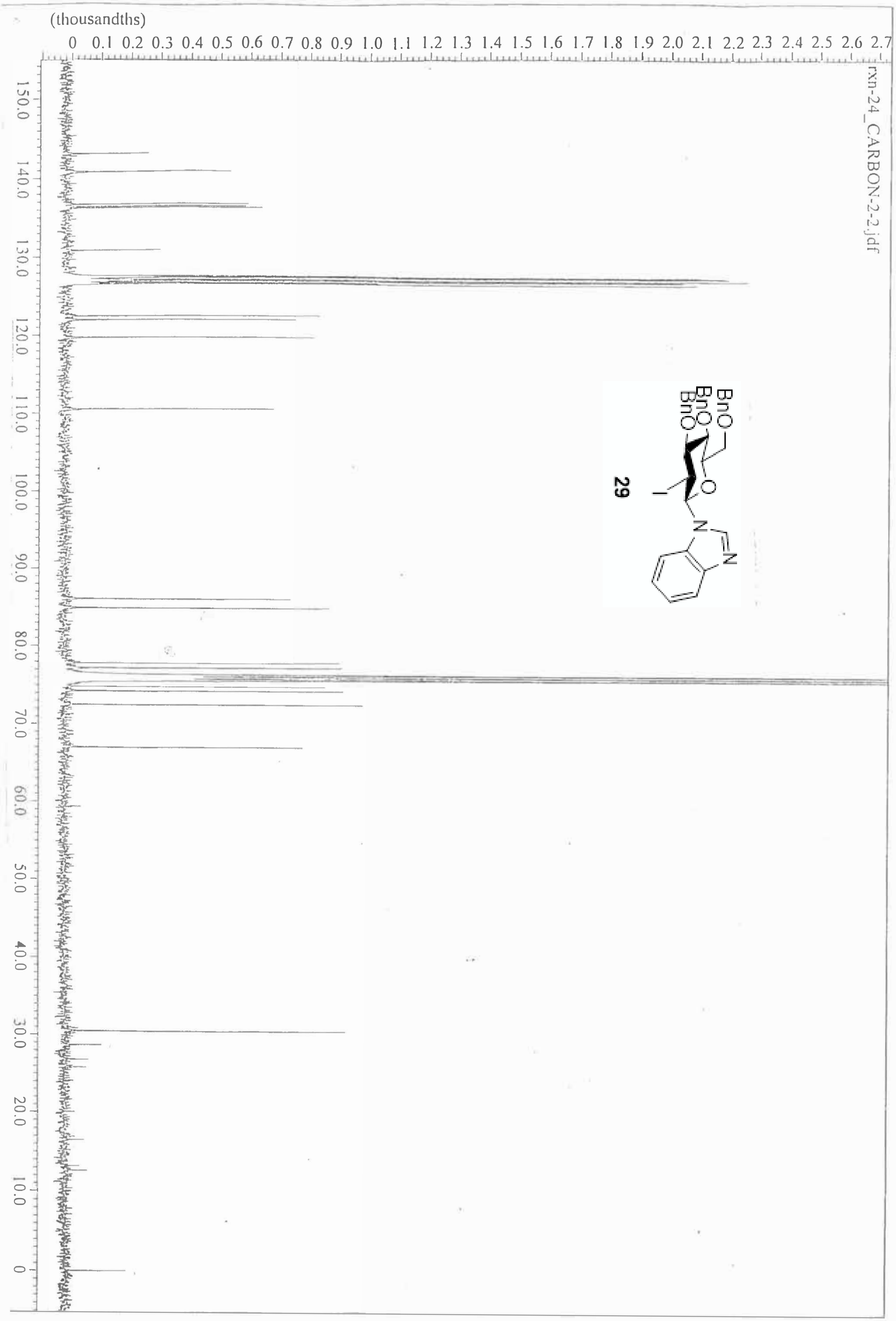

(thousandths)
0 0.1 0.2 0.3 0.4 0.5 0.6 0.7 0.8 0.9 1.0 1.1 1.2 1.3 1.4 1.5 1.6 1.7 1.8 1.9 2.0 2.1 2.2 2.3 2.4 2.5 2.6 2.7
150.0 140.0 130.0 120.0 110.0 100.0 90.0 80.0 70.0 60.0 50.0 40.0 30.0 20.0 10.0 0
rxn-24_CARBON-2-2.jdf
BnO
BnO
BnO
O
I
N
N
29

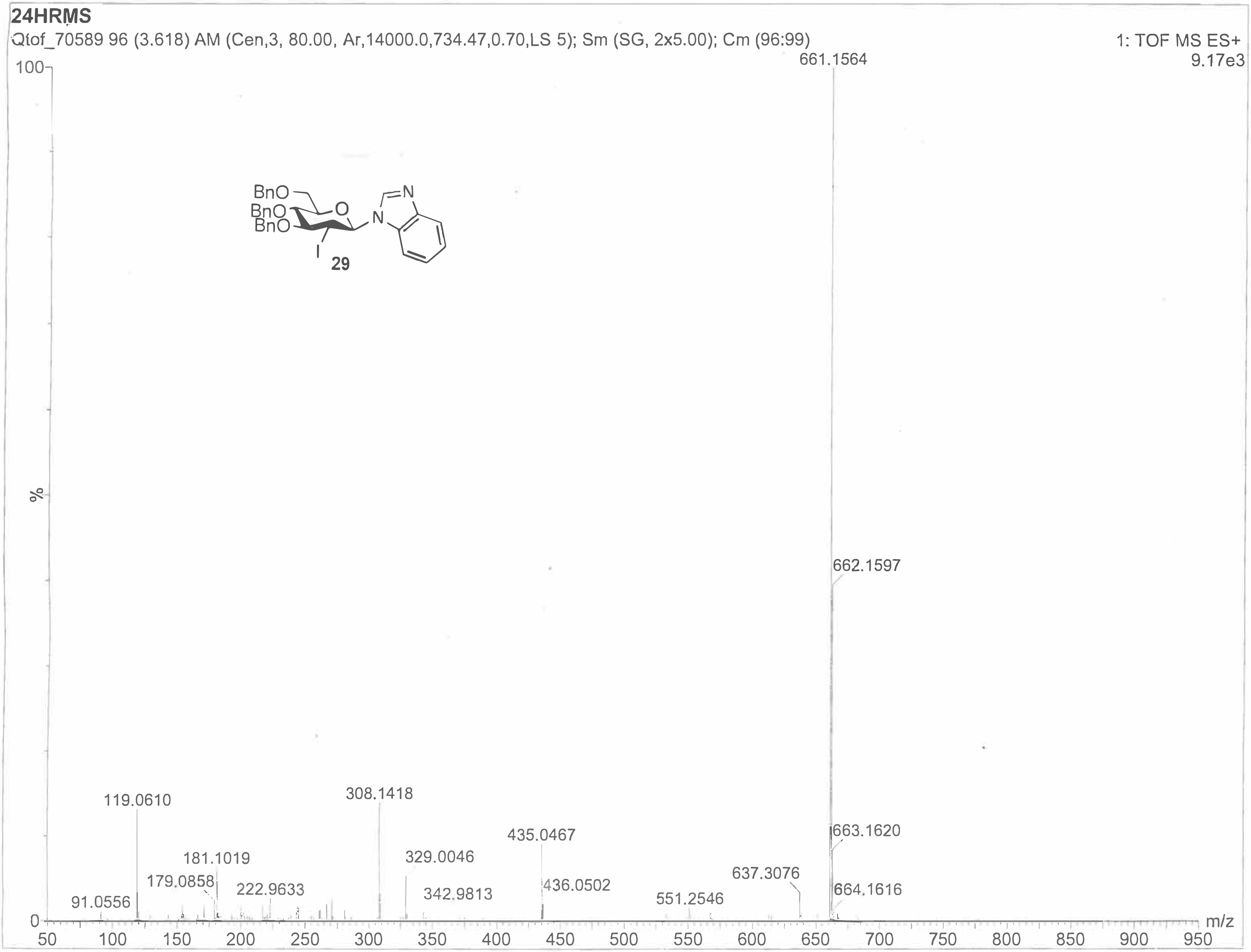
24HRMS
Qtof_70589 96 (3.618) AM (Cen,3, 80.00, Ar,14000.0,734.47,0.70,LS 5); Sm (SG, 2x5.00); Cm (96:99)
1: TOF MS ES+
9.17e3
661.1564
662.1597
663.1620
664.1616
637.3076
551.2546
435.0467
436.0502
329.0046
342.9813
308.1418
222.9633
181.1019
179.0858
119.0610
91.0556
BnO
BnO
BnO
O
N
N
I
29
100
%
0
m/z
50
100
150
200
250
300
350
400
450
500
550
600
650
700
750
800
850
900
950

Single Mass Analysis

Tolerance = 5.0 PPM / DBE: min = -1.5, max = 100.0
Element prediction: Off
Number of isotope peaks used for i-FIT = 3

Monoisotopic Mass, Even Electron Ions
928 formula(e) evaluated with 3 results within limits (up to 50 closest results for each mass)
Elements Used:
C: 0-200 H: 0-200 N: 0-5 O: 4-9 Na: 0-1 I: 0-1

24HRMS
Qtof_70589 96 (3.618) AM (Cen,3, 80.00, Ar,14000.0,734.47,0.70,LS 5); Sm (SG, 2x5.00); Cm (96:99) 1: TOF MS ES+ 9.17e+003

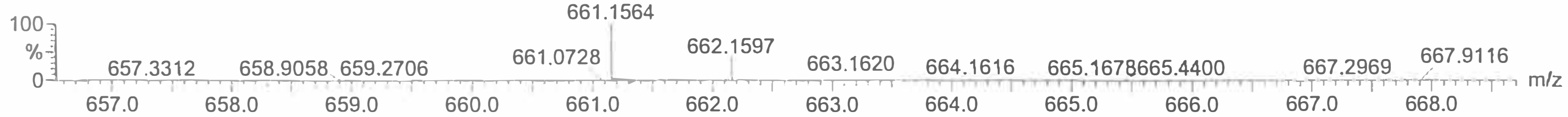

Mass	Calc. Mass	mDa	PPM	DBE	i-FIT	Formula
Minimum:				-1.5		
Maximum:		5.0	5.0	100.0		
661.1564	661.1563	0.1	0.2	18.5	0.2	C34 H34 N2 O4 I
	661.1587	-2.3	-3.5	26.5	36.6	C38 H26 N2 O8 Na
	661.1539	2.5	3.8	15.5	9.9	C32 H35 N2 O4 Na I

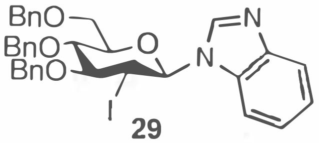

29

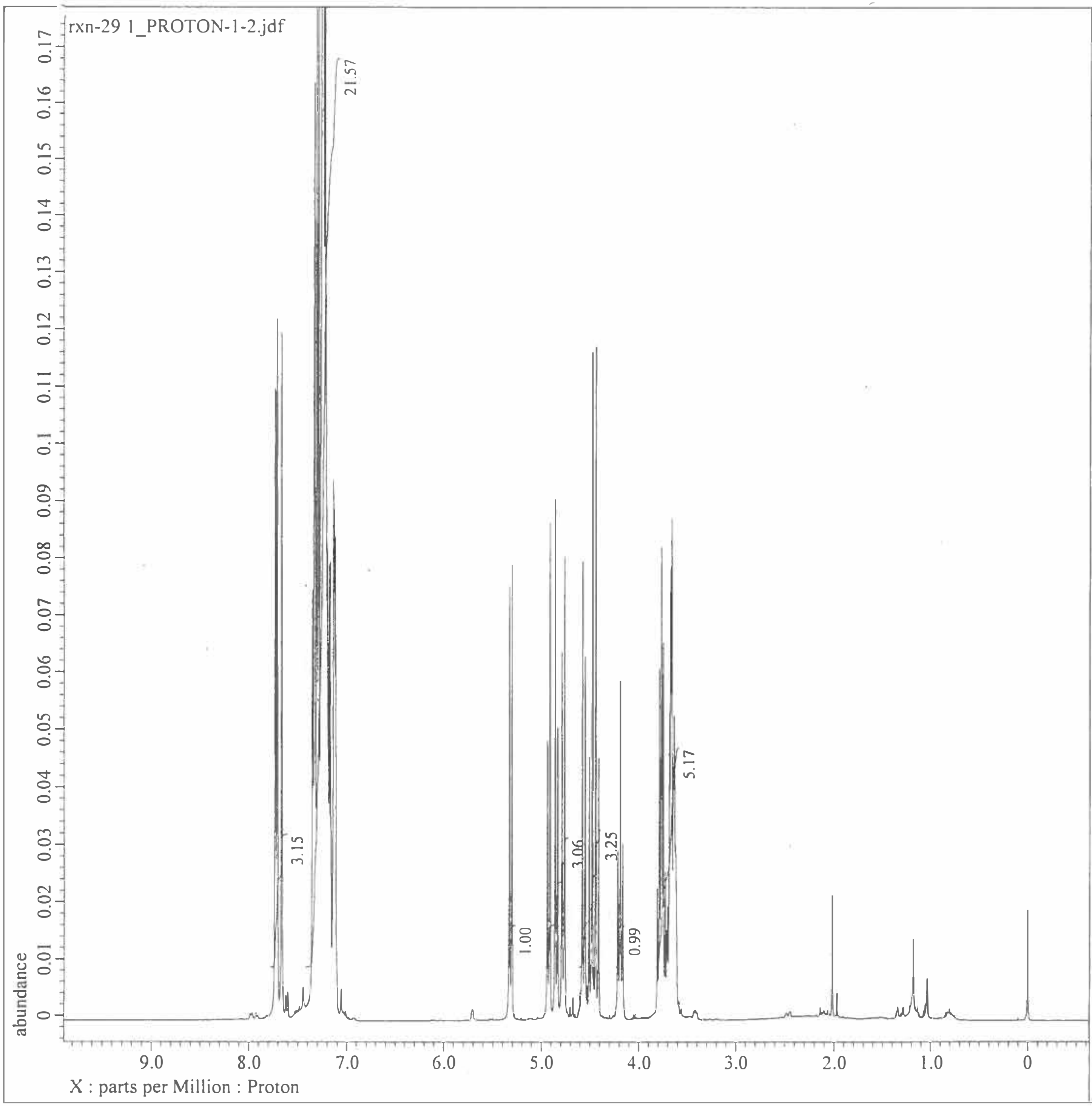

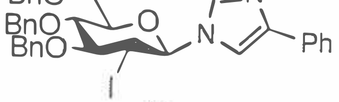

31

Filename	= rxn-29 1_PROTON-1-2
Author	= delta
Experiment	= proton.jxp
Sample_Id	= rxn-29 1
Solvent	= CHLOROFORM-D
Actual_Start_Time	= 9-MAY-2019 12:44:5
Revision_Time	= 9-MAY-2019 13:03:1
Data_Format	= 1D COMPLEX
Dim_Size	= 26214
X_Domain	= Proton
Dim_Title	= Proton
Dim_Units	= [ppm]
Dimensions	= X
Site	= Farmingdale State C
Spectrometer	= JNM-ECZ400S/L1
Field_Strength	= 9.389766[T] (400[MH
X_Acq_Duration	= 4.37256192[s]
X_Domain	= Proton
X_Freq	= 399.78219838[MHz]
X_Offset	= 5[ppm]
X_Points	= 32768
X_Prescans	= 0
X_Resolution	= 0.22869888[Hz]
X_Sweep	= 7.4940048[kHz]
X_Sweep_Clipped	= 5.99520384[kHz]
Irr_Domain	= Proton
Irr_Freq	= 399.78219838[MHz]
Irr_Offset	= 5[ppm]
Tri_Domain	= Proton
Tri_Freq	= 399.78219838[MHz]
Tri_Offset	= 5[ppm]
Blanking	= 2[us]
Clipped	= FALSE
Scans	= 16
Total_Scans	= 16
Relaxation_Delay	= 4[s]
Recvr_Gain	= 42
Temp_Get	= 19.1[dC]
X_90_Width	= 5.85[us]
X_Acq_Time	= 4.37256192[s]
X_Angle	= 45[deg]
X_Atn	= 1.3[dB]
X_Pulse	= 2.925[us]
Irr_Mode	= Off
Tri_Mode	= Off
Dante_Loop	= 400
Dante_Presat	= FALSE
Decimation_Rate	= 0
Experiment_Path	= c:\Program Files\JE
Initial_Wait	= 1[s]
Phase	= {0, 90, 270, 180, 1
Presat_Time	= 4[s]
Presat_Time_Flag	= FALSE
Relaxation_Delay_Calc	= 0[s]
Relaxation_Delay_Temp	= 4[s]
Repetition_Time	= 8.37256192[s]

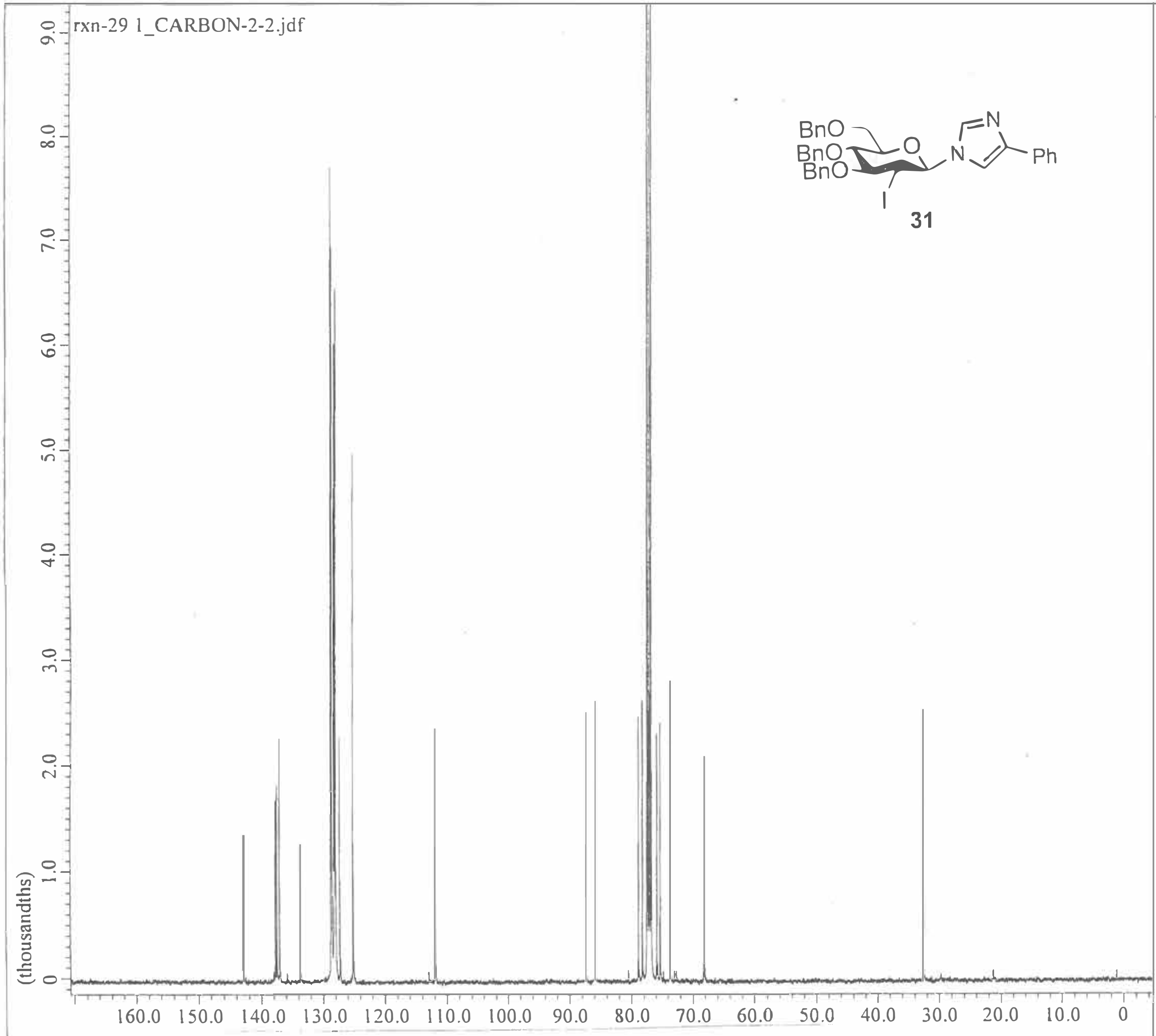

Filename	= rxn-29 1_CARBON-
Author	= decastm
Experiment	= carbon.jxp
Sample_Id	= rxn-29 1
Solvent	= CHLOROFORM-D
Actual_Start_Time	= 9-MAY-2019 23:5
Revision_Time	= 13-MAY-2019 13:3
Data_Format	= 1D COMPLEX
Dim_Size	= 26214
X_Domain	= Carbon13
Dim_Title	= Carbon13
Dim_Units	= [ppm]
Dimensions	= X
Site	= Farmingdale Stat
Spectrometer	= JNM-ECZ400S/L1
Field_Strength	= 9.389766[T] (400
X_Acq_Duration	= 1.03809024[s]
X_Domain	= Carbon13
X_Freq	= 100.52530333[MHz
X_Offset	= 100[ppm]
X_Points	= 32768
X_Prescans	= 4
X_Resolution	= 0.96330739[Hz]
X_Sweep	= 31.56565657[kHz]
X_Sweep_Clipped	= 25.25252525[kHz]
Irr_Domain	= Proton
Irr_Freq	= 399.78219838[MHz
Irr_Offset	= 5[ppm]
Blanking	= 5[us]
Clipped	= FALSE
Scans	= 10000
Total_Scans	= 10000
Relaxation_Delay	= 2[s]
Recvr_Gain	= 52
Temp_Get	= 19.6[dC]
X_90_Width	= 11.73[us]
X_Acq_Time	= 1.03809024[s]
X_Angle	= 30[deg]
X_Atn	= 7.9[dB]
X_Pulse	= 3.91[us]
Irr_Atn_Dec	= 27[dB]
Irr_Atn_Dec_Calc	= 27[dB]
Irr_Atn_Dec_Default_Calc	= 27[dB]
Irr_Atn_Noe	= 27[dB]
Irr_Dec_Bandwidth_Hz	= 4.7826087[kHz]
Irr_Dec_Bandwidth_Ppm	= 11.96303566[ppm]
Irr_Dec_Freq	= 399.78219838[MHz
Irr_Dec_Merit_Factor	= 2.2
Irr_Decoupling	= TRUE
Irr_Noe	= TRUE
Irr_Noise	= WALTZ
Irr_Offset_Default	= 5[ppm]
Irr_Pwidth	= 0.115[ms]
Irr_Pwidth_Default	= 0.115[ms]
Irr_Pwidth_Default_Calc	= 0.115[ms]
Irr_Pwidth_Templ	= 0.115[ms]
Irr_Wurst	= FALSE
Decimation_Rate	= 0
Experiment_Path	= c:\Program Files
Initial_Wait	= 1[s]
Noe_Time	= 2[s]

Michael De Castro mdc-29-N

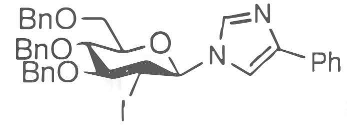

31

SYNAPTG2-Si#NotSet
12:05:54
1: TOF MS ES+
5.52e6

Synapt_22436 39 (0.775)

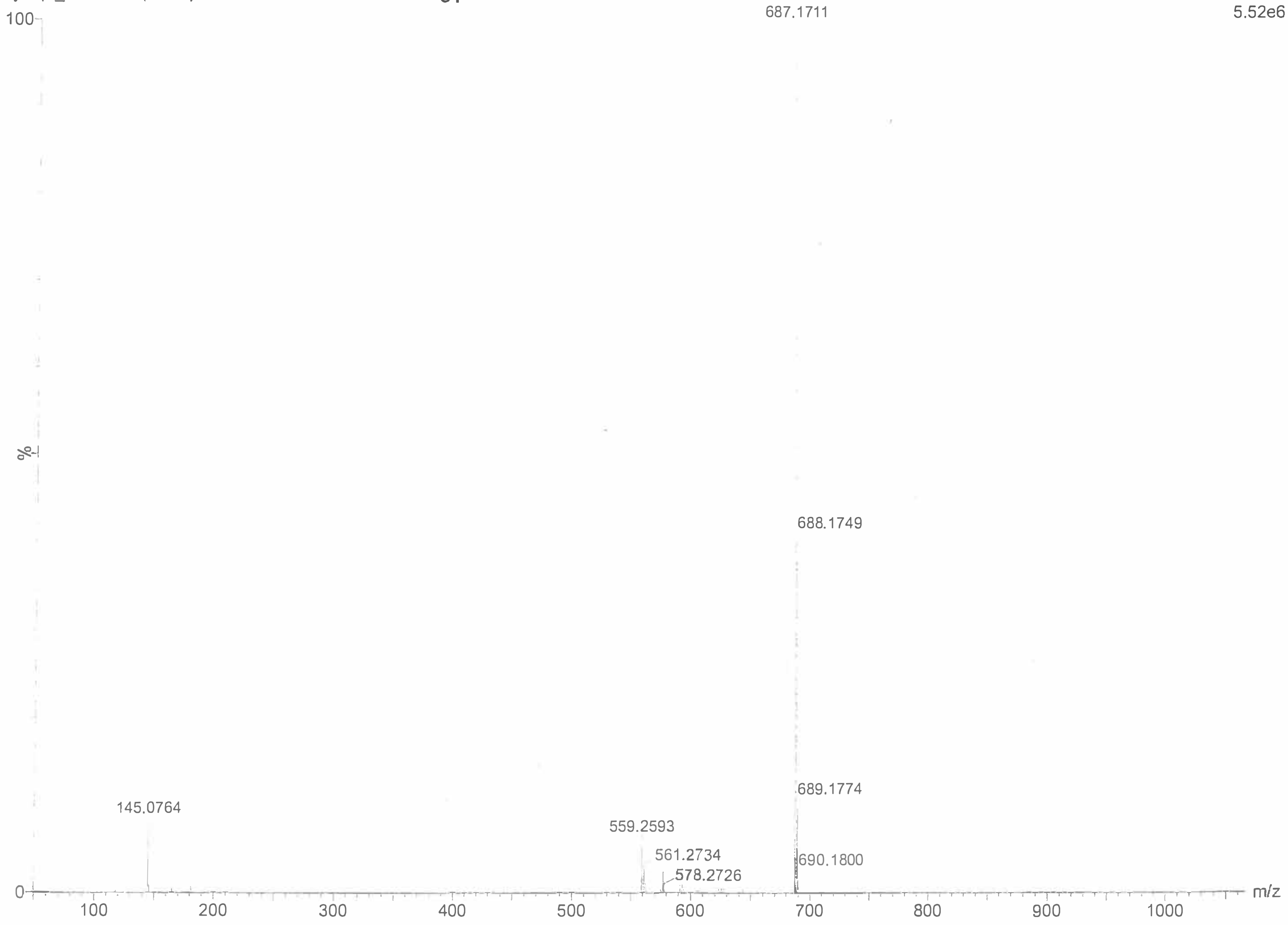

Elemental Composition Report

Single Mass Analysis

Tolerance = 5.0 PPM / DBE: min = -50.0, max = 100.0
Element prediction: Off
Number of isotope peaks used for i-FIT = 9

Monoisotopic Mass, Even Electron Ions
14 formula(e) evaluated with 1 results within limits (up to 10 best isotopic matches for each mass)
Elements Used:
C: 0-50 H: 0-80 N: 2-2 O: 4-4 I: 0-1

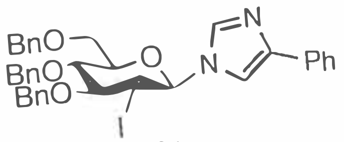

31

Minimum:				-50.0				
Maximum:		5.0	5.0	100.0				
Mass	Calc. Mass	mDa	PPM	DBE	i-FIT	Norm	Conf(%)	Formula
687.1711	687.1720	-0.9	-1.3	19.5	1414.9	n/a	n/a	C36 H36 N2 O4 I

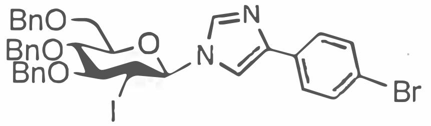

32

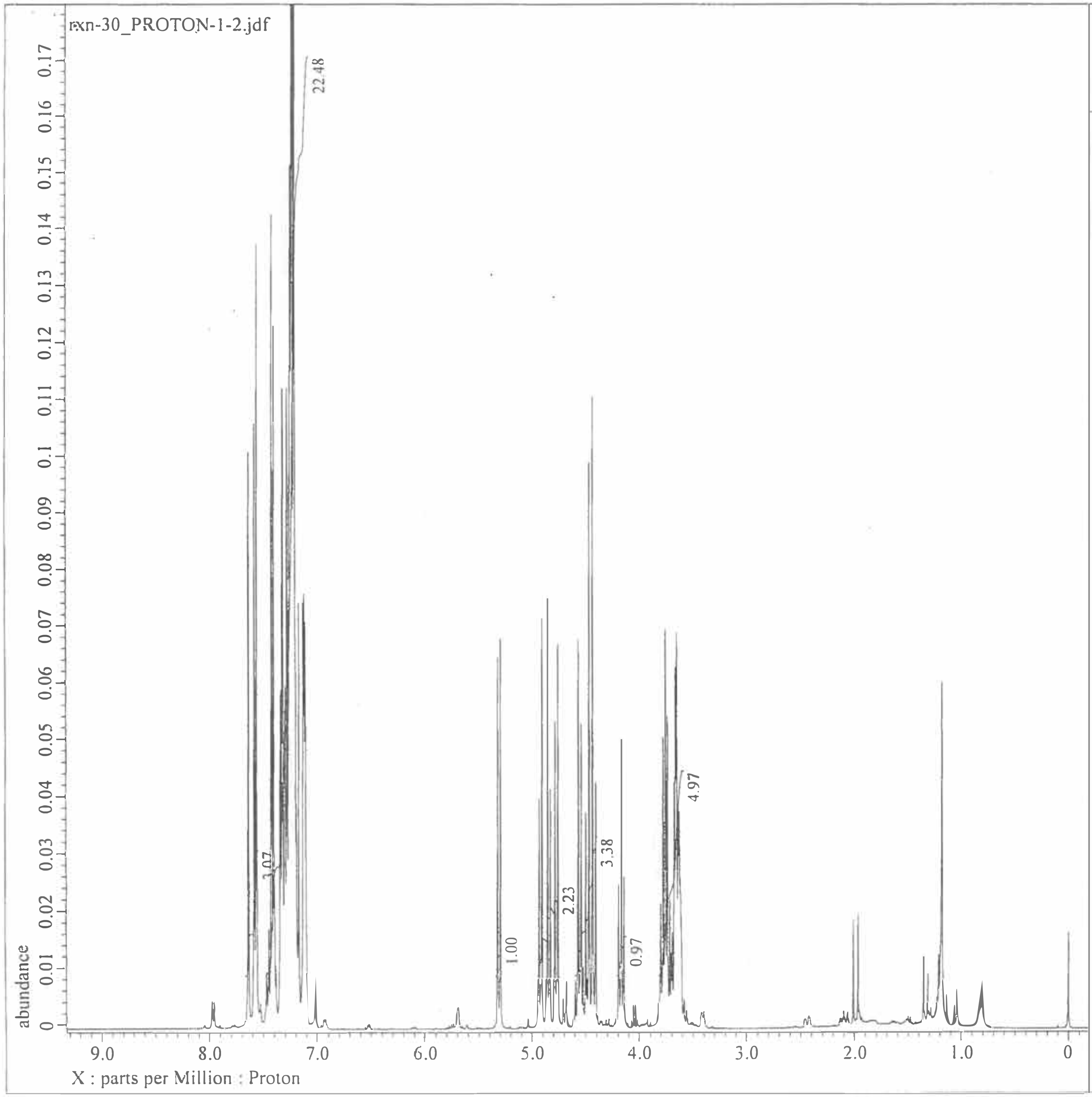


```
Filename                 = rxn-30_PROTON-1-2.j
Author                   = delta
Experiment               = proton.jxp
Sample_Id                = rxn-30
Solvent                  = CHLOROFORM-D
Actual_Start_Time        =  9-MAY-2019 12:50:5
Revision_Time            =  9-MAY-2019 13:23:0

Data_Format              = 1D COMPLEX
Dim_Size                 = 26214
X_Domain                 = Proton
Dim_Title                = Proton
Dim_Units                = [ppm]
Dimensions               = X
Site                     = Farmingdale State C
Spectrometer             = JNM-ECZ400S/L1

Field_Strength           = 9.389766[T] (400[MH
X_Acq_Duration           = 4.37256192[s]
X_Domain                 = Proton
X_Freq                   = 399.78219838[MHz]
X_Offset                 = 5[ppm]
X_Points                 = 32768
X_Prescans               = 0
X_Resolution             = 0.22869888[Hz]
X_Sweep                  = 7.4940048[kHz]
X_Sweep_Clipped          = 5.99520384[kHz]
Irr_Domain               = Proton
Irr_Freq                 = 399.78219838[MHz]
Irr_Offset               = 5[ppm]
Tri_Domain               = Proton
Tri_Freq                 = 399.78219838[MHz]
Tri_Offset               = 5[ppm]
Blanking                 = 2[us]
Clipped                  = FALSE
Scans                    = 16
Total_Scans              = 16

Relaxation_Delay         = 4[s]
Recvr_Gain               = 42
Temp_Get                 = 19.5[dC]
X_90_Width               = 5.85[us]
X_Acq_Time               = 4.37256192[s]
X_Angle                  = 45[deg]
X_Atn                    = 1.3[dB]
X_Pulse                  = 2.925[us]
Irr_Mode                 = Off
Tri_Mode                 = Off
Dante_Loop               = 400
Dante_Presat             = FALSE
Decimation_Rate          = 0
Experiment_Path          = c:\Program Files\JE
Initial_Wait             = 1[s]
Phase                    = {0, 90, 270, 180, 1
Presat_Time              = 4[s]
Presat_Time_Flag         = FALSE
Relaxation_Delay_Calc    = 0[s]
Relaxation_Delay_Temp    = 4[s]
Repetition_Time          = 8.37256192[s]
```

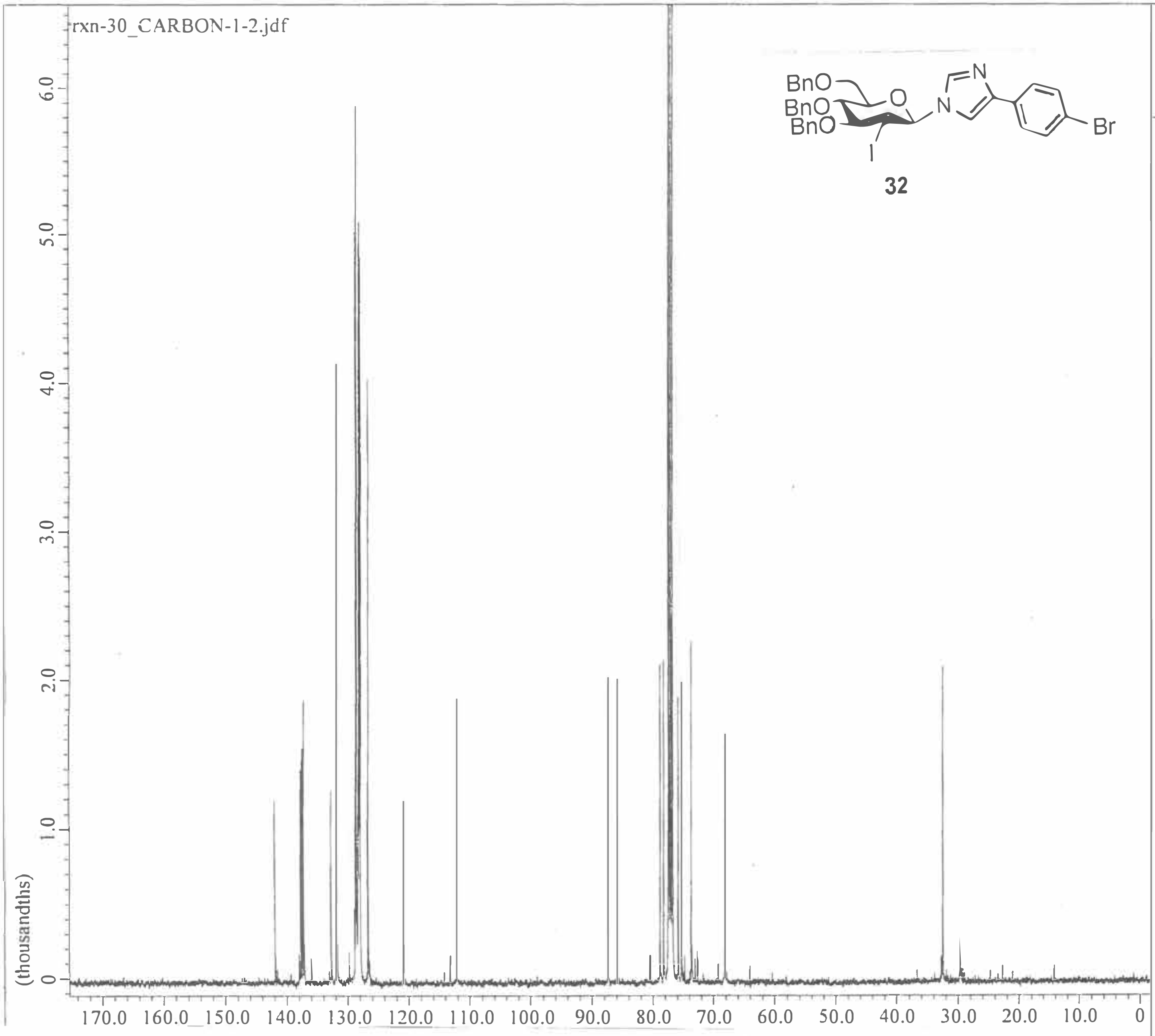

32

JEOL

Filename	= rxn-30_CARBON-1-
Author	= decastm
Experiment	= carbon.jxp
Sample_Id	= rxn-30
Solvent	= CHLOROFORM-D
Actual_Start_Time	= 9-MAY-2019 15:2
Revision_Time	= 9-MAY-2019 23:5
Data_Format	= 1D COMPLEX
Dim_Size	= 26214
X_Domain	= Carbon13
Dim_Title	= Carbon13
Dim_Units	= [ppm]
Dimensions	= X
Site	= Farmingdale Stat
Spectrometer	= JNM-ECZ400S/L1
Field_Strength	= 9.389766[T] (400
X_Acq_Duration	= 1.03809024[s]
X_Domain	= Carbon13
X_Freq	= 100.52530333[MHz
X_Offset	= 100[ppm]
X_Points	= 32768
X_Prescans	= 4
X_Resolution	= 0.96330739[Hz]
X_Sweep	= 31.56565657[kHz]
X_Sweep_Clipped	= 25.25252525[kHz]
Irr_Domain	= Proton
Irr_Freq	= 399.78219838[MHz
Irr_Offset	= 5[ppm]
Blanking	= 5[us]
Clipped	= FALSE
Scans	= 10000
Total_Scans	= 10000
Relaxation_Delay	= 2[s]
Recvr_Gain	= 52
Temp_Get	= 19.2[dC]
X_90_Width	= 11.73[us]
X_Acq_Time	= 1.03809024[s]
X_Angle	= 30[deg]
X_Atn	= 7.9[dB]
X_Pulse	= 3.91[us]
Irr_Atn_Dec	= 27[dB]
Irr_Atn_Dec_Calc	= 27[dB]
Irr_Atn_Dec_Default_Calc	= 27[dB]
Irr_Atn_Noe	= 27[dB]
Irr_Dec_Bandwidth_Hz	= 4.7826087[kHz]
Irr_Dec_Bandwidth_Ppm	= 11.96303566[ppm]
Irr_Dec_Freq	= 399.78219838[MHz
Irr_Dec_Merit_Factor	= 2.2
Irr_Decoupling	= TRUE
Irr_Noe	= TRUE
Irr_Noise	= WALTZ
Irr_Offset_Default	= 5[ppm]
Irr_Pwidth	= 0.115[ms]
Irr_Pwidth_Default	= 0.115[ms]
Irr_Pwidth_Default_Calc	= 0.115[ms]
Irr_Pwidth_Templ	= 0.115[ms]
Irr_Wurst	= FALSE
Decimation_Rate	= 0
Experiment_Path	= c:\Program Files
Initial_Wait	= 1[s]
Noe_Time	= 2[s]

Michael De Castro mdc-100

SYNAPTG2-Si#NotSet
12:03:59

Synapt_22435 33 (0.674)

1: TOF MS ES+
7.59e5

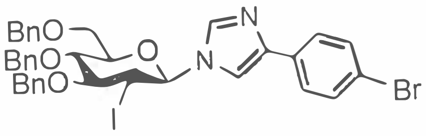

32

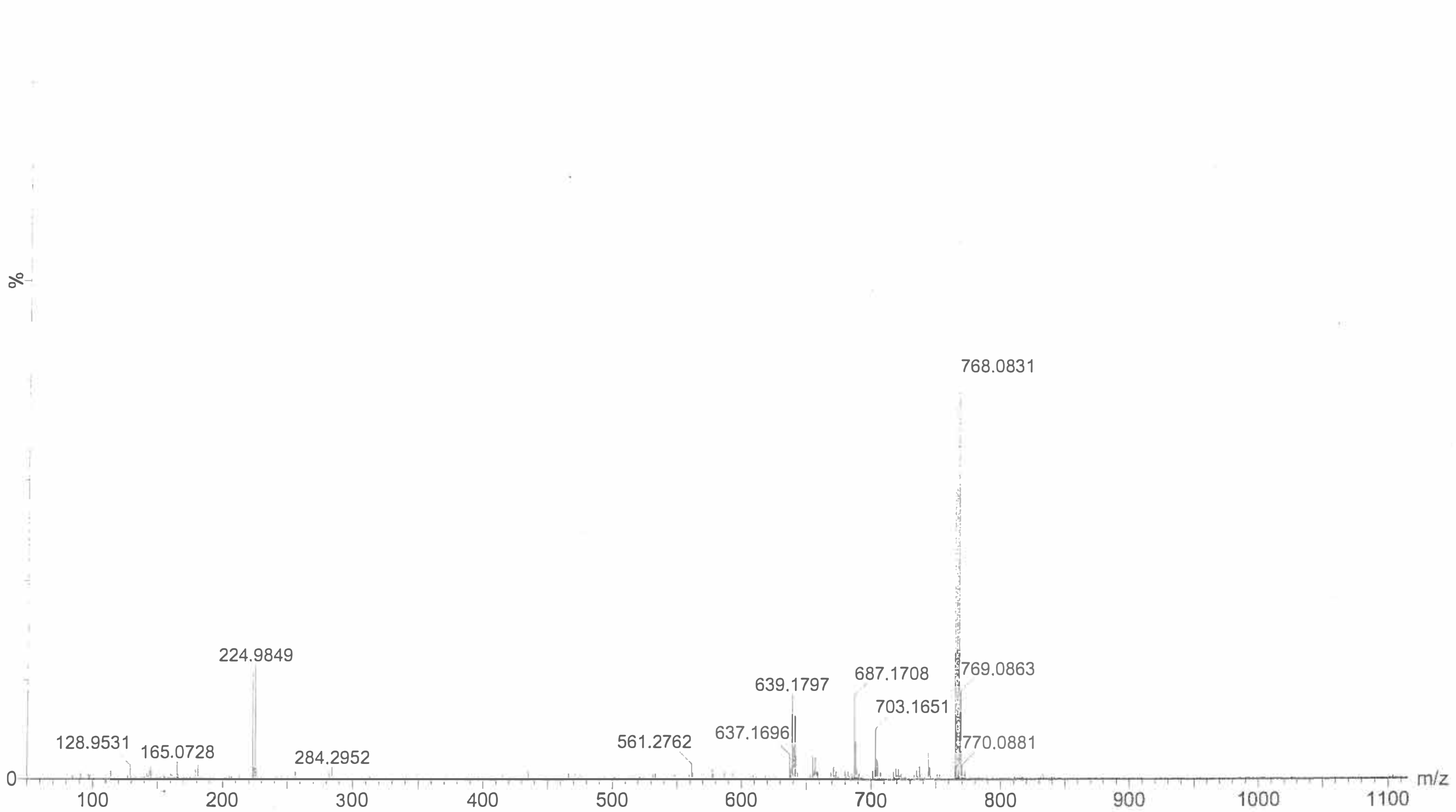

Elemental Composition Report

Single Mass Analysis

Tolerance = 5.0 PPM / DBE: min = -50.0, max = 100.0
Element prediction: Off
Number of isotope peaks used for i-FIT = 9

Monoisotopic Mass, Even Electron Ions
14 formula(e) evaluated with 1 results within limits (up to 10 best isotopic matches for each mass)
Elements Used:
C: 0-50 H: 0-80 N: 2-2 O: 4-4 Br: 1-1 I: 0-1

Minimum:				-50.0				
Maximum:		5.0	5.0	100.0				
Mass	Calc. Mass	mDa	PPM	DBE	i-FIT	Norm	Conf(%)	Formula
765.0823	765.0825	-0.2	-0.3	19.5	1544.1	n/a	n/a	C36 H35 N2 O4 Br I

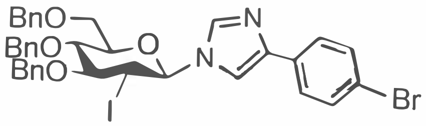

32

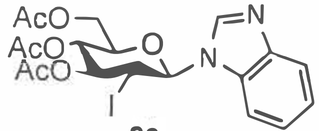

33

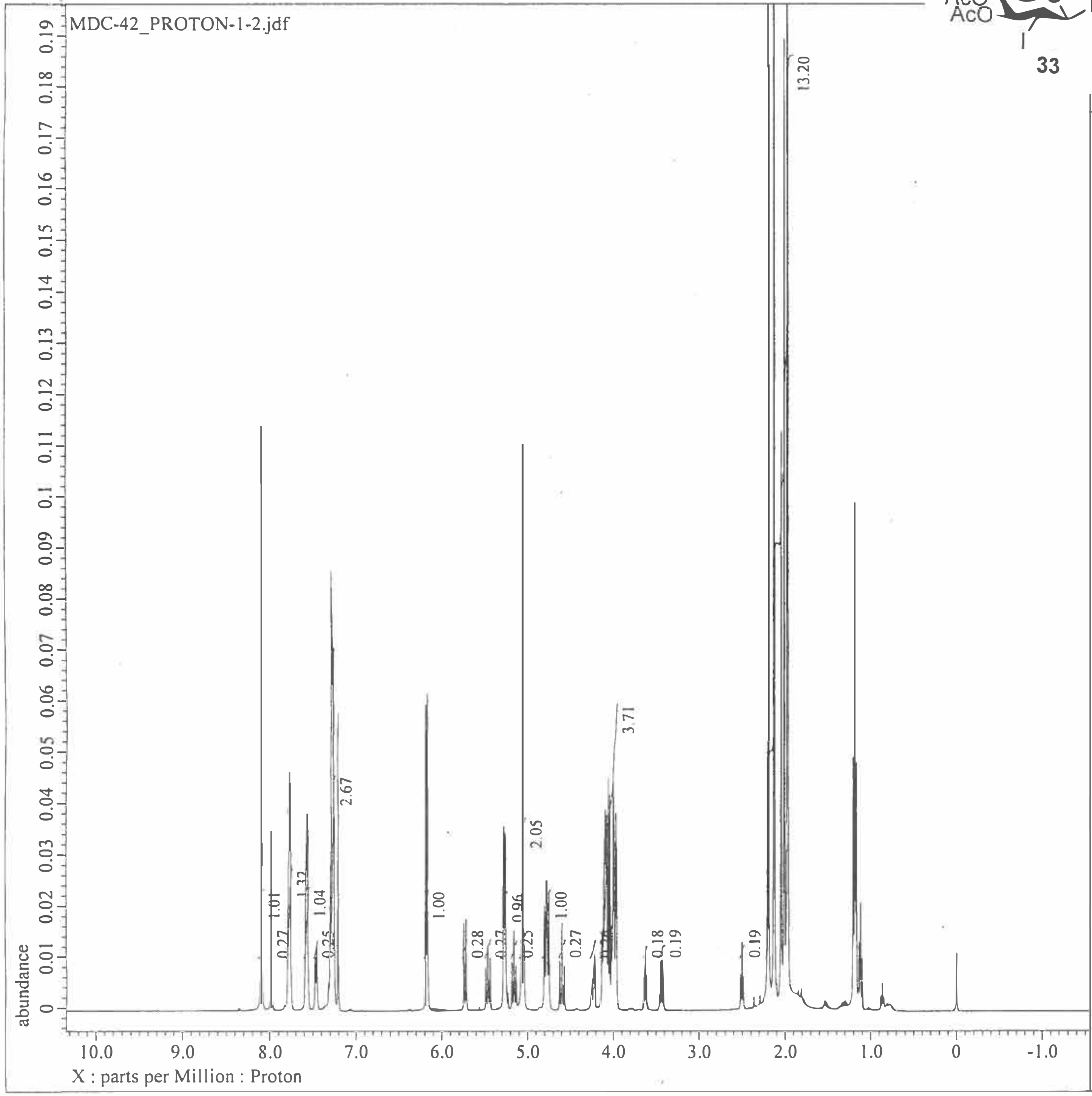

Filename	= MDC-42_PROTON-1-2.j
Author	= decastm
Experiment	= proton.jxp
Sample_Id	= MDC-42
Solvent	= CHLOROFORM-D
Actual_Start_Time	= 6-JUN-2019 16:57:5
Revision_Time	= 6-JUN-2019 17:02:3
Data_Format	= 1D COMPLEX
Dim_Size	= 26214
X_Domain	= Proton
Dim_Title	= Proton
Dim_Units	= [ppm]
Dimensions	= X
Site	= Farmingdale State C
Spectrometer	= JNM-ECZ400S/L1
Field_Strength	= 9.389766[T] (400[MH
X_Acq_Duration	= 4.37256192[s]
X_Domain	= Proton
X_Freq	= 399.78219838[MHz]
X_Offset	= 5[ppm]
X_Points	= 32768
X_Prescans	= 0
X_Resolution	= 0.22869888[Hz]
X_Sweep	= 7.4940048[kHz]
X_Sweep_Clipped	= 5.99520384[kHz]
Irr_Domain	= Proton
Irr_Freq	= 399.78219838[MHz]
Irr_Offset	= 5[ppm]
Tri_Domain	= Proton
Tri_Freq	= 399.78219838[MHz]
Tri_Offset	= 5[ppm]
Blanking	= 2[us]
Clipped	= FALSE
Scans	= 16
Total_Scans	= 16
Relaxation_Delay	= 4[s]
Recvr_Gain	= 42
Temp_Get	= 17.1[dC]
X_90_Width	= 5.85[us]
X_Acq_Time	= 4.37256192[s]
X_Angle	= 45[deg]
X_Atn	= 1.3[dB]
X_Pulse	= 2.925[us]
Irr_Mode	= Off
Tri_Mode	= Off
Dante_Loop	= 400
Dante_Presat	= FALSE
Decimation_Rate	= 0
Experiment_Path	= c:\Program Files\JE
Initial_Wait	= 1[s]
Phase	= {0, 90, 270, 180, 1
Presat_Time	= 4[s]
Presat_Time_Flag	= FALSE
Relaxation_Delay_Calc	= 0[s]
Relaxation_Delay_Temp	= 4[s]
Repetition_Time	= 8.37256192[s]

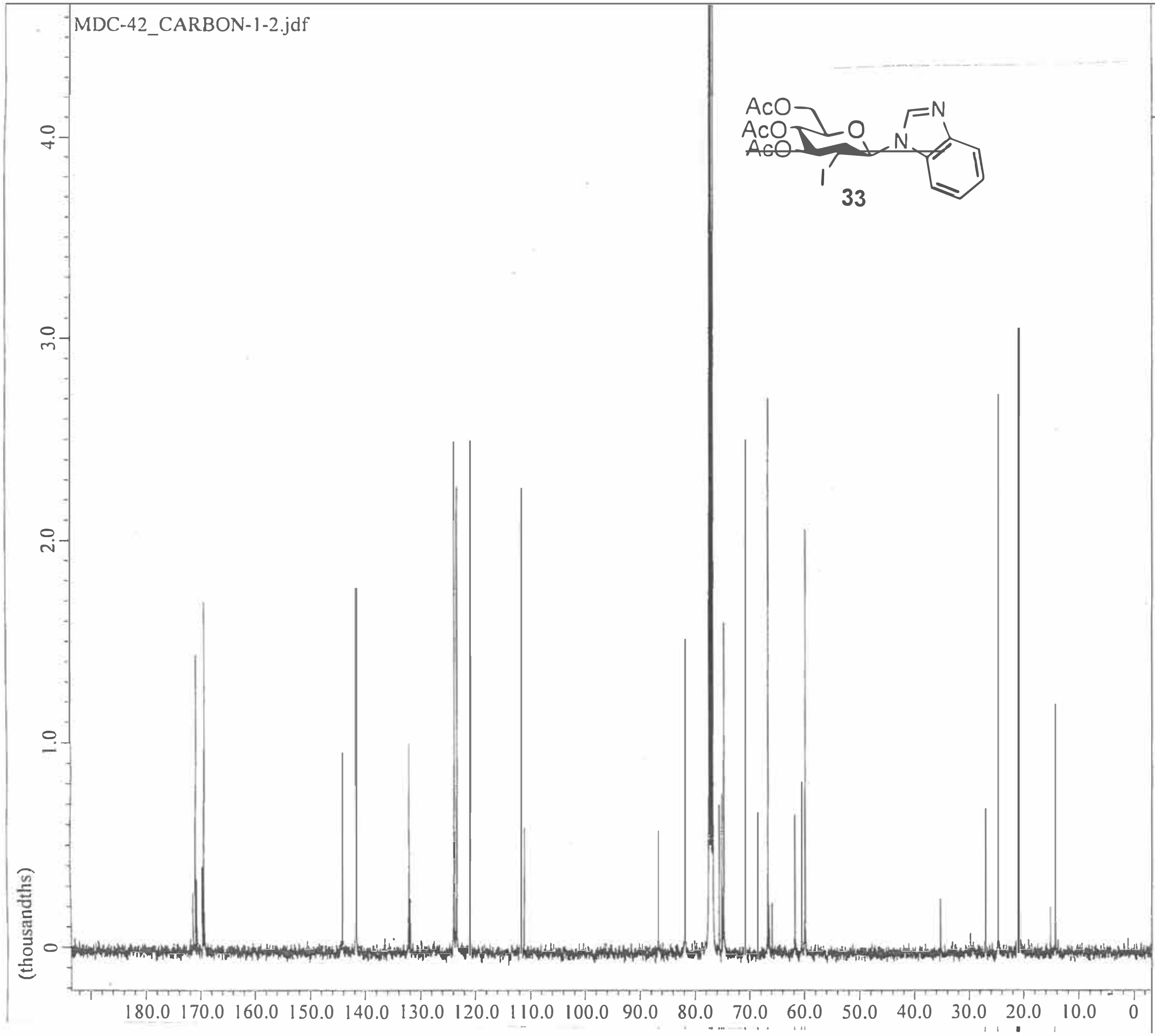

JEOL

Filename	= MDC-42_CARBON-1-
Author	= decastm
Experiment	= carbon.jxp
Sample_Id	= MDC-42
Solvent	= CHLOROFORM-D
Actual_Start_Time	= 6-JUN-2019 17:0
Revision_Time	= 10-JUN-2019 15:0
Data_Format	= 1D COMPLEX
Dim_Size	= 26214
X_Domain	= Carbon13
Dim_Title	= Carbon13
Dim_Units	= [ppm]
Dimensions	= X
Site	= Farmingdale Stat
Spectrometer	= JNM-ECZ400S/L1
Field_Strength	= 9.389766[T] (400
X_Acq_Duration	= 1.03809024[s]
X_Domain	= Carbon13
X_Freq	= 100.52530333[MHz
X_Offset	= 100[ppm]
X_Points	= 32768
X_Prescans	= 4
X_Resolution	= 0.96330739[Hz]
X_Sweep	= 31.56565657[kHz]
X_Sweep_Clipped	= 25.25252525[kHz]
Irr_Domain	= Proton
Irr_Freq	= 399.78219838[MHz
Irr_Offset	= 5[ppm]
Blanking	= 5[us]
Clipped	= FALSE
Scans	= 5000
Total_Scans	= 5000
Relaxation_Delay	= 2[s]
Recvr_Gain	= 52
Temp_Get	= 16.7[dC]
X_90_Width	= 11.73[us]
X_Acq_Time	= 1.03809024[s]
X_Angle	= 30[deg]
X_Atn	= 7.9[dB]
X_Pulse	= 3.91[us]
Irr_Atn_Dec	= 27[dB]
Irr_Atn_Dec_Calc	= 27[dB]
Irr_Atn_Dec_Default_Calc	= 27[dB]
Irr_Atn_Noe	= 27[dB]
Irr_Dec_Bandwidth_Hz	= 4.7826087[kHz]
Irr_Dec_Bandwidth_Ppm	= 11.96303566[ppm]
Irr_Dec_Freq	= 399.78219838[MHz
Irr_Dec_Merit_Factor	= 2.2
Irr_Decoupling	= TRUE
Irr_Noe	= TRUE
Irr_Noise	= WALTZ
Irr_Offset_Default	= 5[ppm]
Irr_Pwidth	= 0.115[ms]
Irr_Pwidth_Default	= 0.115[ms]
[illegible]	[illegible][ms]
[illegible]	[illegible][ms]
[illegible]	[illegible]
[illegible]	[illegible]ogram Files

Michael De Castro mdc-42-N

SYNAPTG2-Si#NotSet
12:11:56

Synapt_22439 32 (0.637)

1: TOF MS ES+
1.31e6

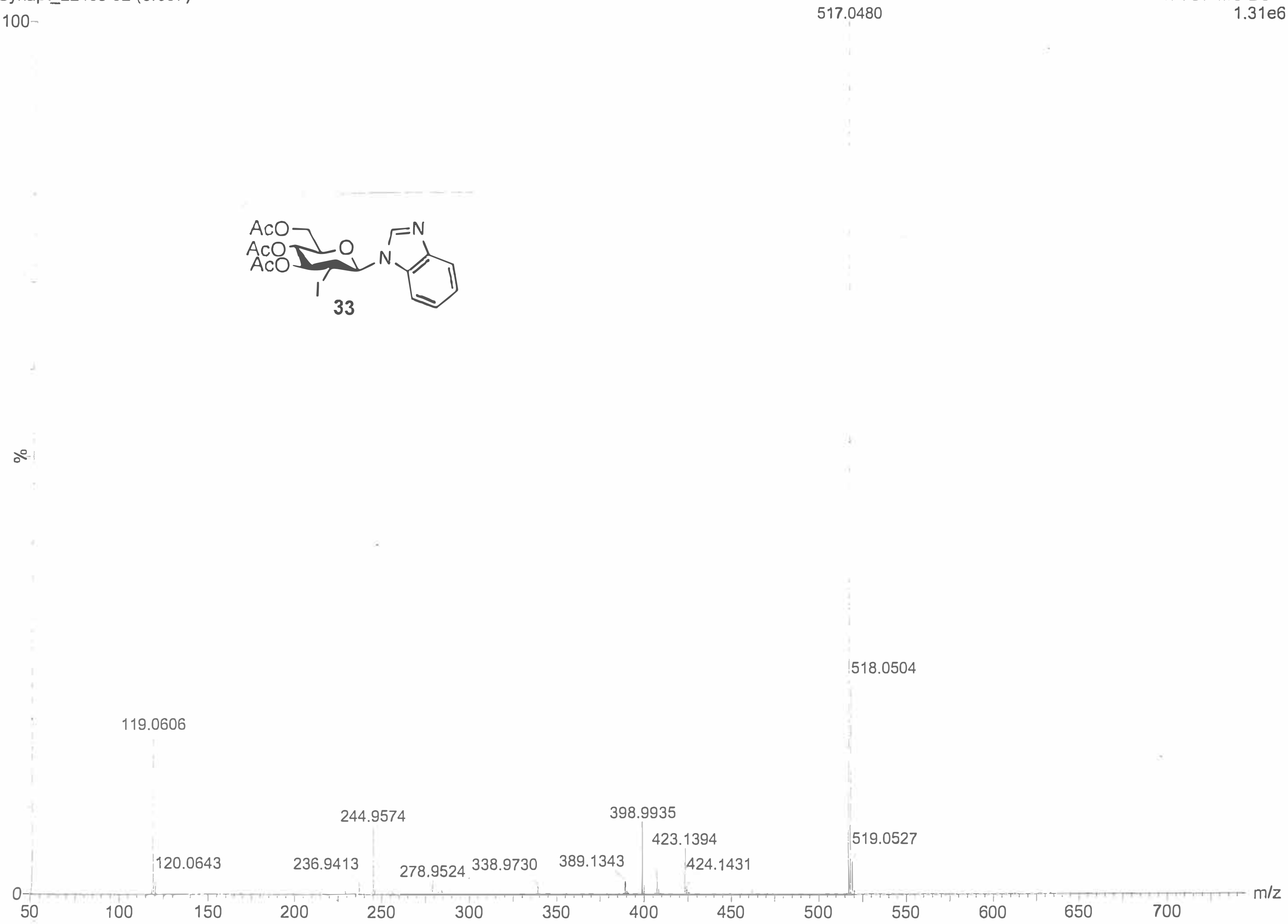

Single Mass Analysis

Tolerance = 5.0 PPM / DBE: min = -50.0, max = 100.0
Element prediction: Off
Number of isotope peaks used for i-FIT = 9

Monoisotopic Mass, Even Electron Ions
13 formula(e) evaluated with 1 results within limits (up to 10 best isotopic matches for each mass)
Elements Used:
C: 0-50 H: 0-80 N: 2-2 O: 7-7 I: 0-1

Minimum:				-50.0				
Maximum:		5.0	5.0	100.0				
Mass	Calc. Mass	mDa	PPM	DBE	i-FIT	Norm	Conf(%)	Formula
517.0480	517.0472	0.8	1.5	9.5	258.8	n/a	n/a	C19 H22 N2 O7 I

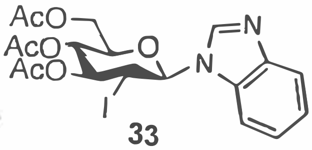

33

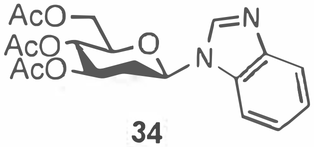

34

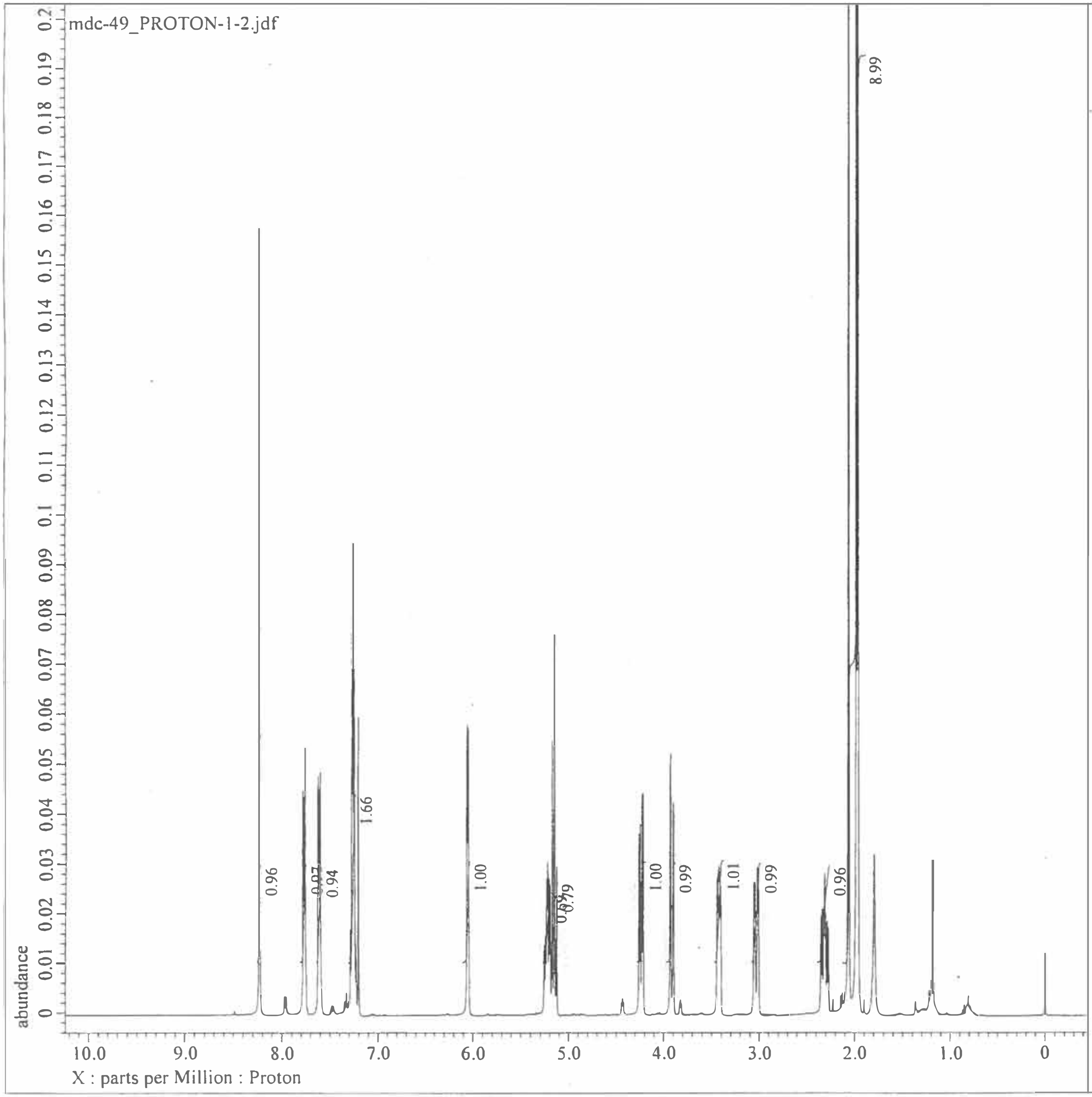

Filename	= mdc-49_PROTON-1-2.j
Author	= decastm
Experiment	= proton.jxp
Sample_Id	= mdc-49
Solvent	= CHLOROFORM-D
Actual_Start_Time	= 25-JUN-2019 12:03:2
Revision_Time	= 25-JUN-2019 13:31:4
Data_Format	= 1D COMPLEX
Dim_Size	= 26214
X_Domain	= Proton
Dim_Title	= Proton
Dim_Units	= [ppm]
Dimensions	= X
Site	= Farmingdale State C
Spectrometer	= JNM-ECZ400S/L1
Field_Strength	= 9.389766[T] (400[MH
X_Acq_Duration	= 4.37256192[s]
X_Domain	= Proton
X_Freq	= 399.78219838[MHz]
X_Offset	= 5[ppm]
X_Points	= 32768
X_Prescans	= 0
X_Resolution	= 0.22869888[Hz]
X_Sweep	= 7.4940048[kHz]
X_Sweep_Clipped	= 5.99520384[kHz]
Irr_Domain	= Proton
Irr_Freq	= 399.78219838[MHz]
Irr_Offset	= 5[ppm]
Tri_Domain	= Proton
Tri_Freq	= 399.78219838[MHz]
Tri_Offset	= 5[ppm]
Blanking	= 2[us]
Clipped	= FALSE
Scans	= 16
Total_Scans	= 16
Relaxation_Delay	= 4[s]
Recvr_Gain	= 42
Temp_Get	= 17.2[dC]
X_90_Width	= 5.85[us]
X_Acq_Time	= 4.37256192[s]
X_Angle	= 45[deg]
X_Atn	= 1.3[dB]
X_Pulse	= 2.925[us]
Irr_Mode	= Off
Tri_Mode	= Off
Dante_Loop	= 400
Dante_Presat	= FALSE
Decimation_Rate	= 0
Experiment_Path	= c:\Program Files\JE
Initial_Wait	= 1[s]
Phase	= {0, 90, 270, 180, 1
Presat_Time	= 4[s]
Presat_Time_Flag	= FALSE
Relaxation_Delay_Calc	= 0[s]
Relaxation_Delay_Temp	= 4[s]
Repetition_Time	= 8.37256192[s]

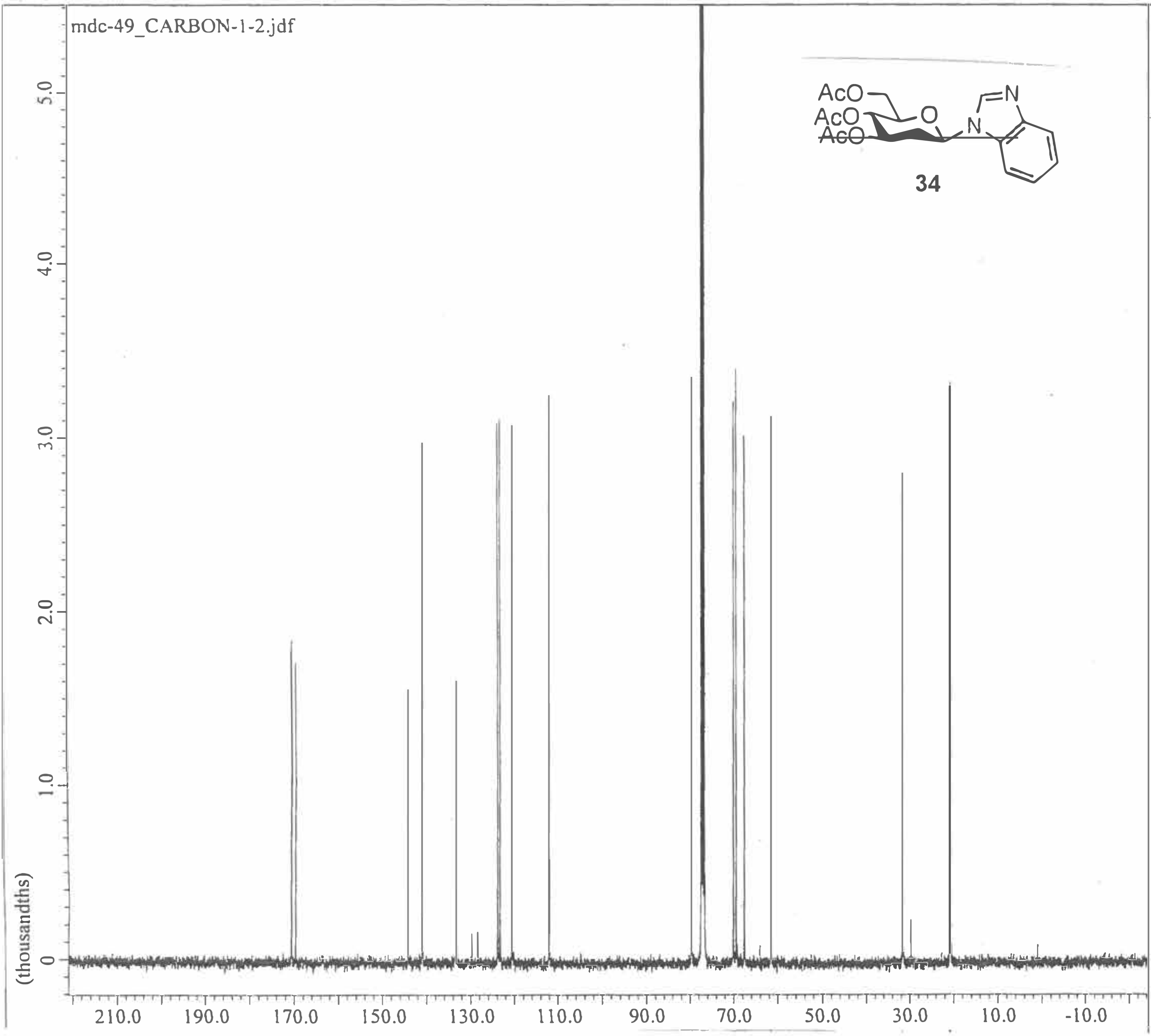

JEOL

```
Filename                      = mdc-49_CARBON-1-
Author                        = decastm
Experiment                    = carbon.jxp
Sample_Id                     = mdc-49
Solvent                       = CHLOROFORM-D
Actual_Start_Time             = 25-JUN-2019 12:3
Revision_Time                 = 25-JUN-2019 16:4

Data_Format                   = 1D COMPLEX
Dim_Size                      = 26214
X_Domain                      = Carbon13
Dim_Title                     = Carbon13
Dim_Units                     = [ppm]
Dimensions                    = X
Site                          = Farmingdale Stat
Spectrometer                  = JNM-ECZ400S/L1

Field_Strength                = 9.389766[T] (400
X_Acq_Duration                = 1.03809024[s]
X_Domain                      = Carbon13
X_Freq                        = 100.52530333[MHz
X_Offset                      = 100[ppm]
X_Points                      = 32768
X_Prescans                    = 4
X_Resolution                  = 0.96330739[Hz]
X_Sweep                       = 31.56565657[kHz]
X_Sweep_Clipped               = 25.25252525[kHz]
Irr_Domain                    = Proton
Irr_Freq                      = 399.78219838[MHz
Irr_Offset                    = 5[ppm]
Blanking                      = 5[us]
Clipped                       = FALSE
Scans                         = 5000
Total_Scans                   = 5000

Relaxation_Delay              = 2[s]
Recvr_Gain                    =
Temp_Get                      = 17[dC]
X_90_Width                    = 11.73[us]
X_Acq_Time                    = 1.03809024[s]
X_Angle                       = 30[deg]
X_Atn                         = 7.9[dB]
X_Pulse                       = 3.91[us]
Irr_Atn_Dec                   = 27[dB]
Irr_Atn_Dec_Calc              = 27[dB]
Irr_Atn_Dec_Default_Calc      = 27[dB]
Irr_Atn_Noe                   = 27[dB]
Irr_Dec_Bandwidth_Hz          = 4.7826087[kHz]
Irr_Dec_Bandwidth_Ppm         = 11.96303566[ppm]
Irr_Dec_Freq                  = 399.78219838[MHz
Irr_Dec_Merit_Factor          = 2.2
Irr_Decoupling                = TRUE
Irr_Noe                       = TRUE
Irr_Noise                     = WALTZ
Irr_Offset_Default            = 5[ppm]
Irr_Pwidth                    = 0.115[ms]
[illegible]
```

Michael De Castro mdc-49

Synapt_22440 32 (0.637)

SYNAPTG2-Si#NotSet
12:13:58
1: TOF MS ES+
6.51e5

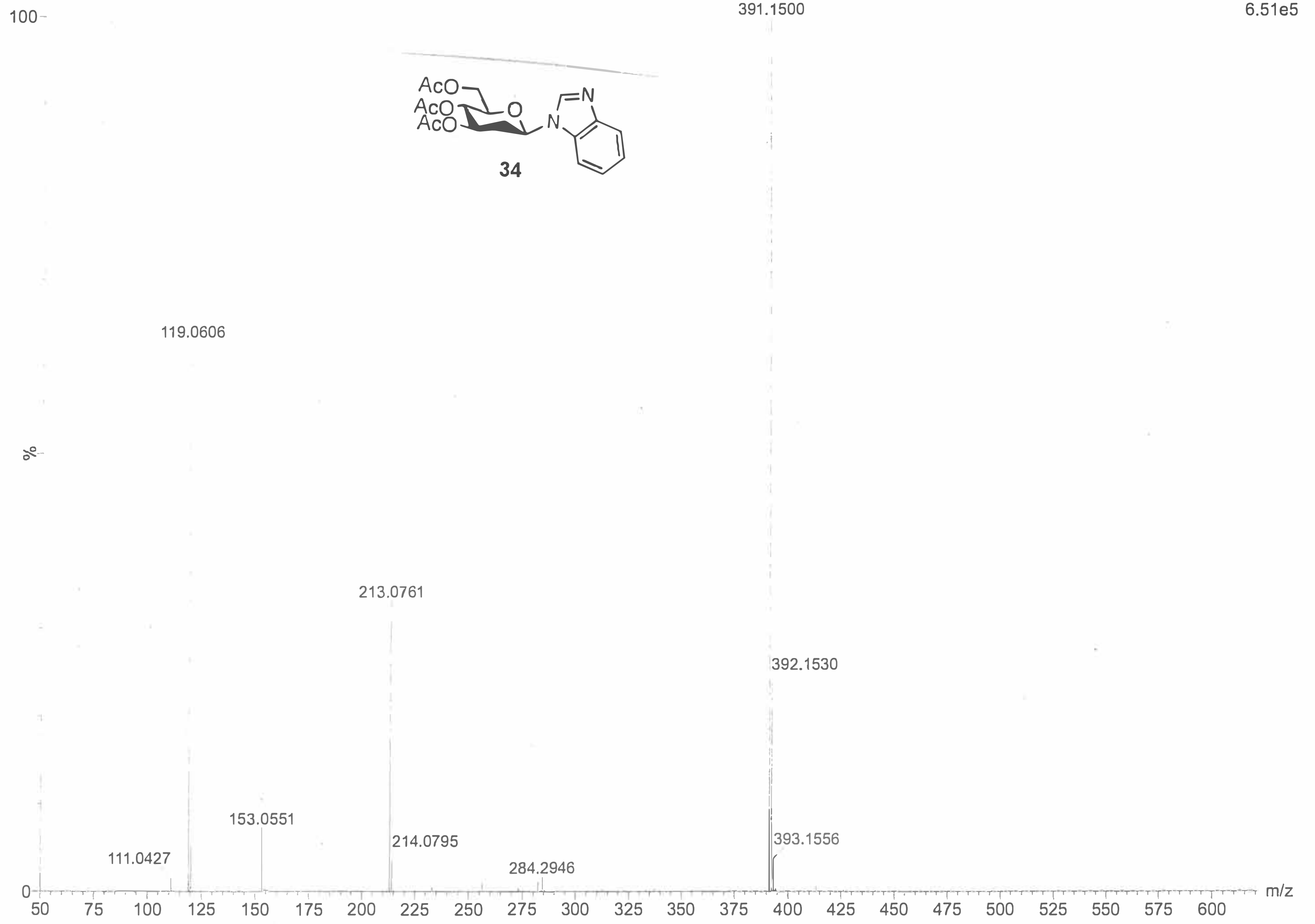

Elemental Composition Report

Single Mass Analysis
Tolerance = 5.0 PPM / DBE: min = -50.0, max = 100.0
Element prediction: Off
Number of isotope peaks used for i-FIT = 9

Monoisotopic Mass, Even Electron Ions
7 formula(e) evaluated with 1 results within limits (up to 10 best isotopic matches for each mass)
Elements Used:
C: 0-50 H: 0-80 N: 2-2 O: 7-7

Minimum:				-50.0				
Maximum:		5.0	5.0	100.0				
Mass	Calc. Mass	mDa	PPM	DBE	i-FIT	Norm	Conf(%)	Formula
391.1500	391.1505	-0.5	-1.3	9.5	267.3	n/a	n/a	C19 H23 N2 O7

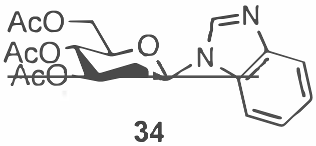

34
